# Extru-seq: a method for predicting genome-wide Cas9 off-target sites with advantages of both cell-based and in vitro approaches

**DOI:** 10.1186/s13059-022-02842-4

**Published:** 2023-01-10

**Authors:** Jeonghun Kwon, Minyoung Kim, Woochang Hwang, Anna Jo, Gue-Ho Hwang, Minhee Jung, Un Gi Kim, Gang Cui, Heonseok Kim, Joon-Ho Eom, Junho K. Hur, Junwon Lee, Youngho Kim, Jin-soo Kim, Sangsu Bae, Jungjoon K. Lee

**Affiliations:** 1 Toolgen, Seoul, Republic of Korea; 2 Department of Pre-Medicine, College of Medicine, Hanyang University, Seoul, Republic of Korea; 3 Hanyang Institute of Bioscience and Biotechnology, Hanyang University, Seoul, Republic of Korea; 4 Department of Chemistry, Hanyang University, Seoul, Republic of Korea; 5 Department of Ophthalmology, Yonsei University College of Medicine, Seoul, Republic of Korea; 6 Department of Medicine, Division of Oncology, Stanford University, Stanford, USA; 7 National Institute of Food and Drug Safety Evaluation, Cheongju, Republic of Korea; 8 Department of Genetics, College of Medicine, Hanyang University, Seoul, Republic of Korea; 9 Center for Genome Engineering, Institute for Basic Science (IBS), Seoul, Republic of Korea; 10 Department of Biochemistry and Molecular Biology, Seoul National University College of Medicine, Seoul, Republic of Korea

**Keywords:** CRISPR, Genome-wide, Off-target, Cell-based, In vitro

## Abstract

We present a novel genome-wide off-target prediction method named Extru-seq and compare it with cell-based (GUIDE-seq), in vitro (Digenome-seq), and in silico methods using promiscuous guide RNAs with large numbers of valid off-target sites. Extru-seq demonstrates a high validation rate and retention of information about the intracellular environment, both beneficial characteristics of cell-based methods. Extru-seq also shows a low miss rate and could easily be performed in clinically relevant cell types with little optimization, which are major positive features of the in vitro methods. In summary, Extru-seq shows beneficial features of cell-based and in vitro methods.

**Supplementary Information:**

The online version contains supplementary material available at 10.1186/s13059-022-02842-4.

## Background

Since 2005, Investigational New Drug (IND) applications have been filed for a variety of genome editors based on zinc finger nucleases, transcription activator-like effector nucleases (TALENs), and clustered regularly interspaced short palindromic repeats (CRISPR) nucleases [1]. Unlike other drugs based on chemicals or antibodies, which are associated with side effects that are typically reversible, the effects of genome editing drugs are permanent. Because such effects, which can frequently be generated at unwanted locations (i.e., off-target effects), raise important safety concerns, the genome-wide identification of off-target sites is of particular importance for genome editing drugs. To this end, several groups have developed a spectrum of experimental methods to predict possible genome-wide off-target effects, which involve different approaches (or strategies), including cell-based (e.g., GUIDE-seq [2], GUIDE-tag [3], DISCOVER-seq [4], BLISS [5], BLESS [6], integrase-defective lentiviral vector-mediated DNA break capture [7], HTGTS [8], ONE-seq [9], CReVIS-Seq [10], ITR-seq [11], and TAG-seq [12]), in vitro (e.g., Digenome-seq [13], DIG-seq [14], SITE-seq [15], CIRCLE-seq [16], and CHANGE-seq [17]), and in silico (e.g., Cas-OFFinder [18], CHOPCHOP [19], and CRISPOR [20]) methods.

However, currently available prediction tools have limitations. For example, cell-based methods occasionally miss bona fide off-target sites and may show diminished efficiencies in clinically more relevant cell types [4, 21]. On the other hand, both in vitro and in silico methods provide too many false-positive data points and do not reflect features of the intracellular environment, such as chromatin structure and epigenetic modifications [14]. In this regard, it would be beneficial to use multiple, complementing methods to determine off-target effects. In a recent study performed by Intellia, GUIDE-seq, SITE-seq, and Cas-OFFinder were used to identify potential off-target sites for the investigational therapy NTLA-2001 [22]. In another case, EDITAS Medicine used three different off-target prediction tools (GUIDE-seq, Digenome-seq, and Cas-OFFinder) for the candidate therapeutic EDIT-101 [23]. By adopting three different prediction methods, it is supposed that the possibility of missing valid off-target candidates would be minimized.

Despite the advantages, however, using multiple prediction methods is laborious and difficult for many groups. Furthermore, the use of additional methods may not result in the detection of more off-target candidates. For example, the seven valid off-target sites identified by SITE-seq for NTLA-2001 included all of the valid off-target sites found by GUIDE-seq (which found three valid off-target sites) and Cas-OFFinder (which found three valid off-target sites). In this case, the output from just one in vitro method that does not miss any valid off-target sites would be the same as the output from the three methods combined. In fact, the use of only one off-target prediction method has been thought to be sufficient for several other IND studies [24–26]. On the other hand, in the case of NTLA-2001, SITE-seq identified 475 potential off-target candidates, of which 468 turned out to be false positives. It would be laborious to validate all 475 candidates for each patient or for cells from each organ in clinical studies. In short, the development of a more efficient and accurate genome-wide off-target prediction tool that largely overcomes the limitations of cell-based, in vitro, and in silico methods remains important. Furthermore, the new method should undergo thorough standardized tests to compare its performance with that of previous methods.

In this study, we developed a novel cell-based in vitro method, named Extru-seq, to combine the beneficial features of both cell-based and in vitro methods. To mimic cell-based methods, pre-incubated Cas9-single guide RNA (sgRNA) ribonucleoprotein (RNP) complexes are physically introduced into the genomic DNA of live cells with preserved chromatin structure and epigenetic modifications, possibly resulting in a high validation rate. The cells are rapidly killed so that DNA repair processes are inhibited after Cas9 RNP-mediated DNA cleavage, allowing cleavage rates to accumulate and avoiding missed off-target sites, a characteristic of in vitro prediction tools.

We also performed a set of standardized tests to compare four different metrics [*p*-value, validation rate, miss rate, and area under receiver operating characteristic (ROC) curve] for Extru-seq with those of other methods [cell-based (GUIDE-seq), in vitro (Digenome-seq), and in silico (CAS-OFFinder) methods] using promiscuous guide RNAs with a large number of candidate off-target sites in human and mouse cells. Results from Extru-seq showed high *p*-values only when compared with results from cell-based GUIDE-seq. Extru-seq also resulted in a validation rate and an area under ROC curve that were comparable with that of GUIDE-seq and much higher than that of Digenome-seq and the in silico methods. These results indicate that Extru-seq has cell-based method-like features. On the other hand, Extru-seq seldom missed off-target sites; its miss rate (2.3%) was 12.6-fold less than that of GUIDE-seq (29%). Finally, Extru-seq was easily performed and required little optimization for use in primary cells in which GUIDE-seq could not be used, suggesting that Extru-seq is a versatile and convenient method that combines the positive features of cell-based and in vitro methods.

## Results

### Selection of experimental methods representing three different approaches for genome-wide off-target prediction

**Fig. 1:**
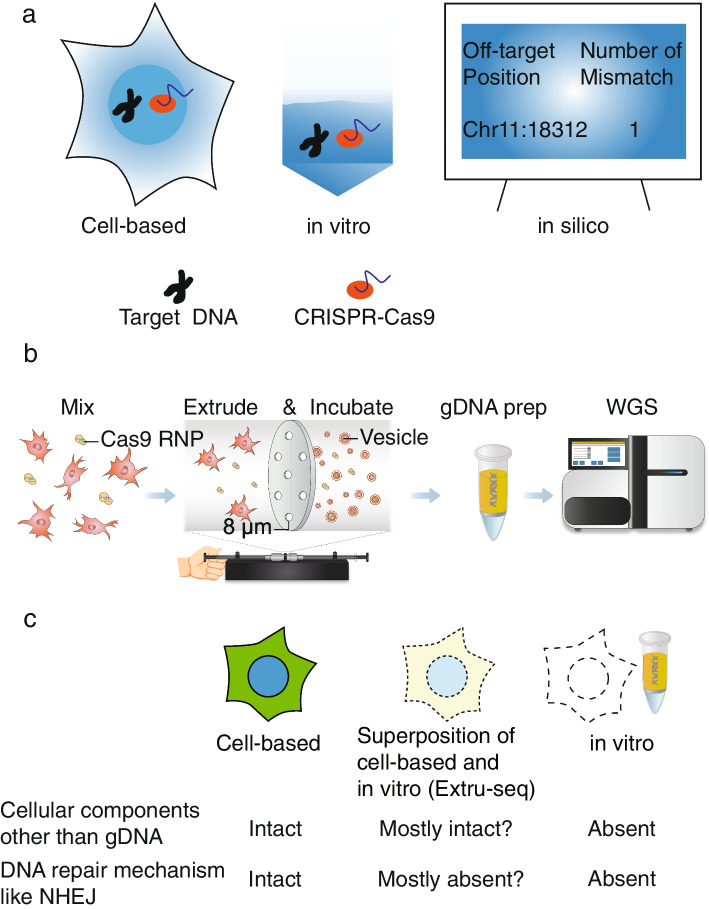
**a** Genome-wide off-target prediction methods can be classified into three different categories: cell-based, in vitro, and in silico. **b** Schematic of the Extru-seq method. **c** Hypothesis about the dual properties of Extru-seq, which shares characteristics with cell-based and in vitro methods

Genome-wide off-target prediction methods can be categorized by their general approach into three major groups: cell-based, in vitro, and in silico (Fig. 1a). Different combinations of methods have been used in IND studies of genome-editing therapeutics (Additional File 1: Fig. S1). We selected one method from each category to compare their performance. For cell-based and in silico approaches, GUIDE-seq and CAS-OFFinder were selected because they were the most frequently used methods in each category for Cas9 therapeutics, including EDIT101 and NTLA-2001. For an in vitro approach, we selected Digenome-seq because it was used in the EDIT101 study and was one of the most popular protocols with a large number of previous studies for comparison.

### Extru-seq: a novel cell-based in vitro method with dual properties

We aimed to design a new method that combines the positive attributes of cell-based and in vitro methods. To this end, we developed Extru-seq, which uses physical force to lyse cells and mix the genomic DNA with Cas9 and the sgRNA (Fig. 1b). In this method, live HEK293T or NIH-3T3 cells are mixed with a pre-incubated Cas9-sgRNA RNP complex. Using an extruder [27], the mixture is forced through filter paper with a pore size smaller than the cell diameter, rupturing the cell membrane and allowing the Cas9 RNPs access to the genomic DNA. Under the optimized conditions (8-μm pore size, 5000 nM Cas9 concentration, 10^7^ cells; Additional File 1: Fig. S2), the quality of the genomic DNA after an overnight incubation with the Cas9 RNPs at 37 °C was high enough for whole genome sequencing (WGS) library construction.

### Measurements of the level of non-homologous end joining (NHEJ) after the extrusion step

We hypothesized that DNA repair mechanisms would not exist in Extru-seq to re-ligate genomic DNA cut by Cas9. Indeed, when the cleavage rates at on-target sites were measured using quantitative PCR, an average rate of 70% was observed (Additional File 1: Fig. S3a to h), indicating that DNA repair mechanisms like NHEJ were absent, reflecting the in vitro nature of the protocol. [We further analyzed both cut and un-cut populations of the on-target sites to investigate the degree to which NHEJ occurs after the extrusion step. First, the un-cut population in the Extru-seq sample was analyzed via deep-sequencing, given that indel mutations would accumulate in the un-cut population if the NHEJ process were intact during the incubation period after the extrusion step. However, when the deep sequencing results for the Extru-seq samples treated with Cas9 RNP complexes were compared with those for the untreated control samples, the differences were not significant (Additional File 1: Fig. S3i), suggesting that the level of NHEJ after the extrusion step is not significant. Second, using the protocol from multiplex Digenome-seq [28], we performed multiplex Extru-seq to measure the change in cleavage rates at five different on-target sites in the presence or absence of 1 μM SCR7, a chemical DNA ligase IV (or NHEJ) inhibitor [29]. If NHEJ were occurring, then the presence of SCR7 should result in an increase in the cleavage rate, an effect that would also accumulate during the incubation step. However, the difference in the average cleavage rates at five on-target sites in the presence or absence of SCR7 was not significant (Additional File 1: Fig. S3j), further indicating that NHEJ does not significantly affect on-target cleavage rates.]

We further hypothesized that the cellular components other than genomic DNA would still be intact such that the cleavage pattern would resemble cell-based off-target prediction method. The hypothesis would later be tested by comparing the Extru-seq results with that of cell based and in vitro-based methods. Like Schrödinger’s cat, which is a metaphor for the wave-particle duality of light, Extru-seq is expected to show dual properties, with characteristics of both cell-based (cellular components other than genomic DNA intact) and in vitro methods (loss of DNA repair mechanism, Fig. 1c).

### Design and use of promiscuous guide sequences

The second aim of the study was to undertake standard tests that could effectively measure performance metrics for each method. Previous studies [2, 13] used guide sequences that were predicted to recognize only a low number of off-target sites in the genome to compare different methods. As a result, only a few validated off-target loci were found, making effective comparisons between different prediction methods, with a statistically meaningful number of loci, difficult. In more recent papers [4, 30], promiscuous guide sequences predicted to recognize a high number of off-target loci were used, providing a powerful test bed for genome-wide off-target prediction methods.

However, these promiscuous guide sequences were not used in this study. One of them, targeting *PCSK9*, involved a mouse guide sequence that is not complementary to sequences in human cells, whereas another, targeting *VEGFA*, lacked predicted off-target loci with a single mismatch (Additional File 1: Fig. S4). To overcome this limitation, we searched for two promiscuous guide sequences, respectively targeting *PCSK9* and *Albumin* in the mouse genome, that also have perfectly matched target sequences present in the human genome. Even though the guide sequences targeted loci other than *PCSK9* or *Albumin* in the human genome, for convenience we labeled them as human *PCSK9* and human *Albumin*. The number of candidate off-target sequences for these guide sequences in both genomes was then calculated using Cas-OFFinder. The selected guide sequences were associated with a high number of candidate off-target sequences in both genomes.

### Predicting genome-wide off-target sites with GUIDE-seq, Digenome-seq, the in silico method, and Extru-seq

**Fig. 2:**
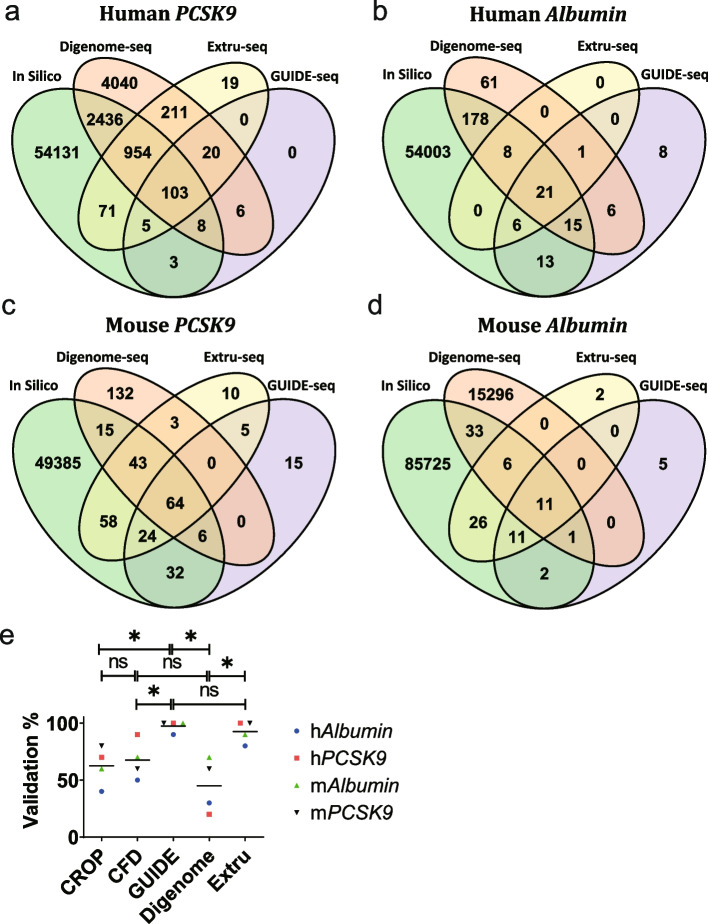
Venn diagrams showing the number of predicted off-target sites for sgRNAs targeting **a** human *PCSK9*, **b** human *Albumin*, **c** mouse *PCSK9*, and **d** mouse *Albumin*, determined by the indicated methods. **e** Validation rates of the top off-target sites predicted by an in silico method, GUIDE-seq, Digenome-seq, and Extru-seq in human and mouse cells for promiscuous sgRNAs targeting the *PCSK9* and *Albumin* genes. The horizontal lines represent the mean (*P < 0.05, ns, no significance in two-sided unpaired Mann-Whitney test)

Using the promiscuous sgRNA sequences targeting *PCSK9* and *Albumin*, GUIDE-seq (Additional File 1: Fig. S5), Digenome-seq (Additional File 1: Fig. S6), Extru-seq (Additional File 1: Fig. S7), and in silico predictions based on Cas-OFFinder were performed for human (HEK293T) and mouse (NIH-3T3) cell lines (Fig. 2a–d). The sequence read counts from GUIDE-seq and the DNA cleavage scores from Digenome-seq and Extru-seq could be used to rank each candidate off-target locus. For in silico predictions made by Cas-OFFinder, there is no score that could be used for such rankings. Therefore, we used two different scripts from machine-learning studies [31, 32] to calculate prediction scores [CRISPR Off-target Predictor (CROP) scores (heuristic scores that indicate if the candidate off-target sites would be edited) and Cutting Frequency Determination (CFD) scores (percent activity values provided in a matrix of penalties based on mismatches of each possible type at each position within the guide RNA sequence)] for each candidate off-target site for ranking purposes. The distribution of sequence read counts, DNA cleavage, and in silico predictive scores for each candidate off-target locus could be tabulated versus the number of mismatches with the guide sequence (Additional File 1: Fig. S8). It is expected that as the number of mismatches increases, the corresponding prediction score would decrease. Whereas GUIDE-seq and in silico predictions followed this trend, outliers with high DNA cleavage scores were found for cases with four, five, or six mismatches in the Digenome-seq results. When DNA cleavage scores for candidate off-target sites were calculated using the Extru-seq approach, high DNA cleavage scores for sgRNAs with more than four mismatches were not observed, in contrast to the results for Digenome-seq. This result may indicate that Extru-seq identified fewer false positives than Digenome-seq; we proceeded to confirm this idea via validation of the off-target candidates with the top scores.

### GUIDE-seq and Extru-seq show high validation rates

Validation of the predicted off-target loci was done using a human cell line and a mouse model. For the human cell line experiment, plasmids encoding the Cas9 protein and sgRNA were transfected into HEK293T cells. For the mouse experiment, sequences encoding the Cas9 protein and sgRNA were packaged into adeno-associated virus (AAV) serotype8 (AAV8). These AAVs were then delivered into C57BL/6 mice via systemic or subretinal injection. Because only subretinal injections resulted in high frequencies of on-target indel formation (Additional File 1: Fig. S9), retinal pigment epithelial cells from the model were used for validation experiments. When the top 10 candidates from each prediction method were examined using targeted deep sequencing, Extru-seq (92.5%) and GUIDE-seq (97.5%) showed significantly higher validation rates compared to Digenome-seq (45%) and the in silico methods [CROP (62.5%) and CFD (67.5%)] on average (Fig. 2e, Additional File 1: Fig. S10).

### Further comparisons between Extru-seq, GUIDE-seq, Digenome-seq, and DIG-seq

Digenome-seq uses purified genomic DNA that has lost elements like chromatin proteins. To overcome this problem, a previous study developed an improved version of Digenome-seq, which was named DIG-seq. DIG-seq, which uses cell-free chromatin DNA rather than histone-free DNA, predicted fewer false positives than Digenome-seq. Because the mild detergent used to lyse cells in the DIG-seq approach could affect the chromatin state of cellular DNA, which could in turn affect the Cas9 cleavage mechanism, we expected that Extru-seq, which uses physical force to lyse cells, would show more characteristics of cell-based methods compared to DIG-seq.

**Fig. 3:**
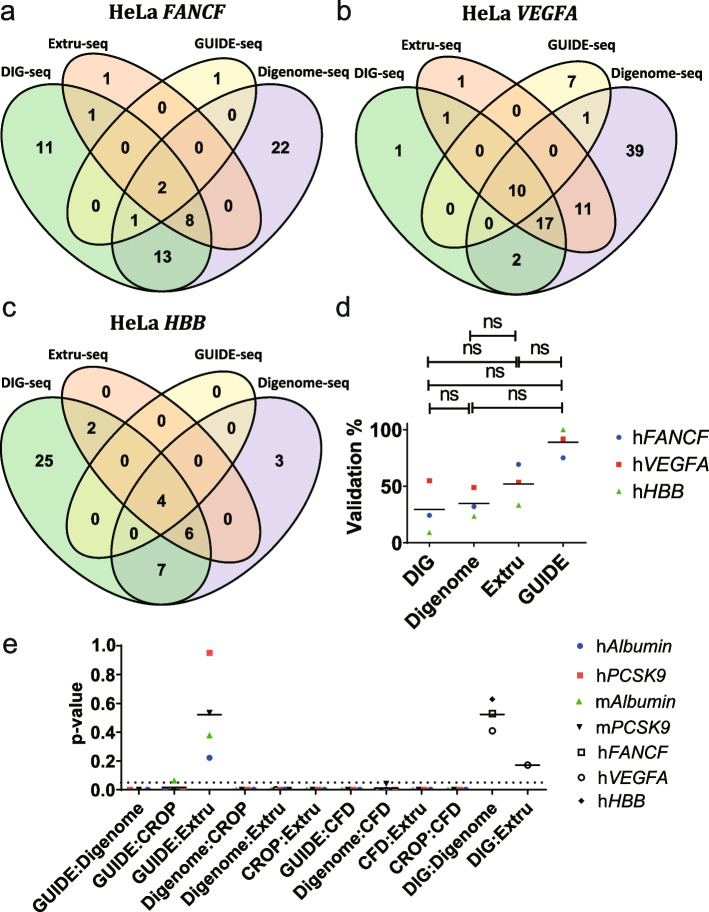
Venn diagrams showing the number of predicted off-target sites for sgRNAs targeting **a** human *FANCF*, **b** human *VEGFA*, and **c** human *HBB*, determined by the indicated methods. **d** Validation rates of the off-target sites predicted by DIG-seq, Digenome-seq, Extru-seq, and GUIDE-seq for sgRNAs targeting the *FANCF*, *VEGFA*, and *HBB* genes in HeLa cells. The horizontal lines represent the mean (ns, no significance in two-sided unpaired Mann-Whitney test). **e** *p*-values obtained by the normalized rank sum test for each pair of off-target prediction methods for sgRNAs targeting *PCSK9* and *Albumin* in human and mouse cells in addition to *FANCF*, *VEGFA*, and *HBB* in HeLa cells. The horizontal lines represent the mean. (*n* ≥ 16 were selected to be analyzed.) Dotted line represents *p* = 0.05

To compare Extru-seq with other in vitro methods further, we performed GUIDE-seq (Additional File 1: Fig. S11) and Extru-seq (Additional File 1: Fig. S12) in HeLa cells with guide sequences targeting *FANCF*, *VEGFA*, and *HBB*, which were used in the original comparison between DIG-seq and Digenome-seq [14]. Venn diagram analysis showed that Digenome-seq and DIG-seq predicted many different off-target loci, whereas most of the off-target loci predicted by Extru-seq were identified by at least one of the other techniques (Fig. 3a–c). When the candidate loci were examined to see if they could be validated, Extru-seq showed a higher validation rate than DIG-seq and Digenome-seq (Fig. 3d, Additional File 1: Fig. S13).

### The rank distributions of the off-target sites predicted by GUIDE-seq and Extru-seq are similar

Another question is whether predictions resulting from Extru-seq and the cell-based, in vitro, and in silico methods are consistent with each other. The top 10 candidate off-target loci were tabulated for each prediction method (Additional File 2: Table S1) and the ranks of these loci in the results from each of the other methods were compared. The number of loci predicted to be in the top 10 by the pair of method was counted and the shared rate calculated, designated as the shared percentage in the top 10. Low similarities (overall average shared percentage in the top 10 = 22%) were observed in most cases. The highest similarities were consistently found between GUIDE-seq and Extru-seq pairwise comparisons (average shared percentage in the top 10 for the GUIDE-seq and Extru-seq pair = 43%).

Comparisons of rank could also be made with all off-target sites including those that were not validated. Venn diagrams show that there are statistically meaningful numbers of candidate off-target sites in the intersections to be analyzed. Scores/read counts were min-max normalized and the Wilcoxon rank sum test was performed to see the equality of the medians for the loci scores in the intersections of results from two methods (Fig. 3e). Because a sample size of at least 16 is required for using asymptotic nonparametric Wilcoxon rank tests [33, 34], intersections with fewer than 16 samples were not included in this analysis (Additional File 1: Fig. S14). The scores of loci from the intersection of two populations are distributed differently if the test shows a low *p*-value. None of the distributions were similar except that of the GUIDE-seq:Extru-seq (average *p*-value = 0.52) and DIG-seq:Digenome-seq (average p-value = 0.52) pairs, which consistently showed high *p*-values with *n* ≥ 3. The disagreements between the results from GUIDE-seq or Extru-seq and the results from Digenome-seq or in silico predictions could be attributed to the low validation rates of Digenome-seq (due to the high number of false positives) and in silico predictions (due to the discrepancy between machine learning-based predictive scores and real-world experimental values). In addition, because the DIG-seq:Digenome-seq pair consistently showed high *p*-values, in contrast to the Digenome-seq:Extru-seq pair, which showed low *p*-values, the results from DIG-seq are more similar to results from in vitro prediction methods (here represented by Digenome-seq), whereas the results from Extru-seq are more similar to results from cell-based prediction methods (here represented by GUIDE-seq) and different from the results from the other in vitro prediction methods like Digenome-seq. In this regard, Extru-seq distinguished itself from DIG-seq in completely losing similarity with the in vitro Digenome-seq method. This result is somewhat surprising, because the experimental procedures for Digenome-seq, DIG-seq, and Extru-seq are all based on WGS, whereas that of GUIDE-seq is based on PCR. It appears that the conditions under which genomic DNA is treated with Cas9 are more important than the rest of the analysis procedures. Moreover, the *p*-value (0.17) of the DIG-seq:Extru-seq pair may indicate that results from DIG-seq are more similar to those from cell-based methods than those from Digenome-seq. However, because only one *p*-value was obtained for the DIG-seq:Extru-seq pair due to the low number of samples in the intersections, this conjecture remains to be proven.

### Extru-seq shows a lower miss rate than GUIDE-seq

**Fig. 4:**
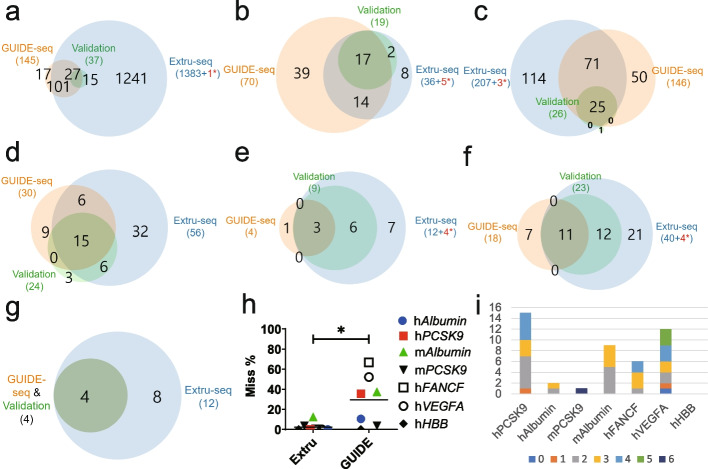
Venn diagrams showing the number of predicted off-target sites for sgRNAs targeting **a** human *PCSK9*, **b** human *Albumin*, **c** mouse *PCSK9*, **d** mouse *Albumin*, **e** human *FANCF*, **f** human *VEGFA*, and **g** human *HBB*, determined by the indicated methods. Validation indicates targets validated by targeted deep sequencing. Red* represents the number of off-target sites that were confirmed manually (Additional File 3: Table S2). **h** Miss rates of Extru-seq and GUIDE-seq determined by considering all of the off-target sites that underwent validation experiments for sgRNAs targeting human *PCSK9*, human *Albumin*, mouse *PCSK9*, mouse *Albumin*, human *FANCF*, human *VEGFA*, and human *HBB* in HeLa cells. The horizontal lines represent the mean (*P < 0.05 in two-sided unpaired Mann-Whitney test). **i** Off-target sites missed by GUIDE-seq and the distribution of the number of mismatches (indicated in parentheses). Deep blue (0), orange (1), gray (2), yellow (3), sky blue (4), green (5), and navy (6)

Cell-based methods, including GUIDE-seq, are known to occasionally miss bona fide off-target candidates. We calculated the miss rate (or the false-negative rate, defined as the number of false negatives/(the number of false negatives + true positives)) using Venn diagrams showing the overlap between Extru-seq and GUIDE-seq predictions and validated targets in samples analyzed by deep-sequencing (Fig. 4a–g, and Additional File 1: Fig. S15). The miss rate of Extru-seq (2.3%) was 12.6-fold lower than that of GUIDE-seq (29%) on average (Fig. 4h). This result indicates that the sensitivity of Extru-seq is significantly higher than that of the cell-based GUIDE-seq method, such that it seldom misses real off-target sites. (Note: Off-target sites detected by Extru-seq that were manually validated are tabulated in Additional File 3: Table S2. It appears that these off-target sites were detected experimentally but missed by the analysis algorithm.) Close inspection revealed that GUIDE-seq overlooked valid off-target sites that contained one to six mismatches (Fig. 4i). As such, an IND study that solely depended on GUIDE-seq [26] would risk overlooking valid off-target candidates. In the case of CTX001, an in silico method was used to complement GUIDE-seq [35]. However, only genomic sites with either three or fewer mismatches, or two or fewer mismatches and a single DNA or RNA bulge, were identified computationally, such that valid off-target sites with more than three mismatches would be overlooked.

### Extru-seq shows the highest area under ROC curves than other methods

**Fig. 5:**
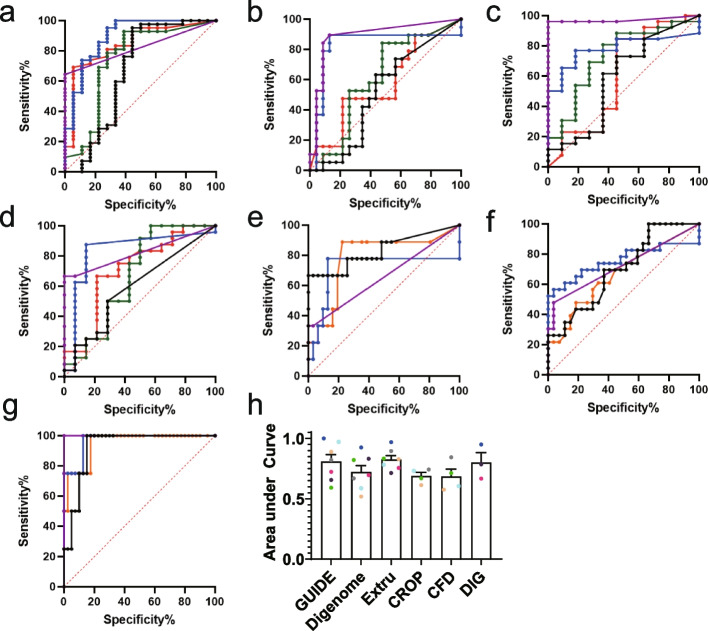
**a**–**d** ROC curves for GUIDE-seq (purple), Digenome-seq (black), Extru-seq (blue), CROP (green), and CFD (red) for the **a** human *PCSK9*, **b** human *Albumin*, **c** mouse *PCSK9*, and **d** mouse *Albumin* sites. **e**–**g** ROC curves for GUIDE-seq (purple), Digenome-seq (black), Extru-seq (blue), and DIG-seq (orange) for the **e** human *FANCF*, **f** human *VEGFA*, and **g** human *HBB* sites. **h** Area the under ROC curves. The bars represent the mean. Error bars indicate standard deviation

One powerful tool for assessing prediction models is the ROC curve, which shows sensitivity and specificity in the *y*-axis and *x*-axis, respectively. We constructed ROC curves using sequence read counts (GUIDE-seq), DNA cleavage scores (Digenome-seq, DIG-seq, and Extru-seq), CFD scores (CFD), or CROP scores (CROP) as the metric to predict validation results as a binary classification (Fig. 5). When the area under the ROC curves were calculated, Extru-seq showed the highest value (0.83) compared to other methods [GUIDE-seq (0.81), DIG-seq (0.80), Digenome-seq (0.72), CROP (0.69), and CFD (0.68) as shown in Fig. 5h]. It is considered that the closer the area under the curve is to one, the better the model in predicting validation results. Therefore, the highest value of area under the ROC curve of Extru-seq suggests the high performance of the DNA cleavage score of Extru-seq as the binary classifier of the validation results. In addition, the use of different thresholds or cut-off values could affect the number of off-target sites predicted by each method. High area under the ROC curve suggest that the chance of finding a meaningful threshold for Extru-seq is higher than that for the other methods.

### Extru-seq can be performed on primary cells with little optimization

The GUIDE-seq method requires a high rate of insertions of double-stranded oligodeoxynucleotides (dsODNs) into double-strand break (DSB) sites, which can be difficult to achieve experimentally for some cell types and conditions. For example, we could not obtain high dsODN insertion rates in primary mesenchymal stem cells (MSCs) derived from bone marrow. In contrast, Extru-seq does not require dsODN insertion. Given this advantage, we performed Extru-seq using MSCs and the promiscuous sgRNAs targeting human *PCSK9* and *Albumin* described above. Venn diagrams (Additional File 1: Fig. S16) show that there are discrepancies between the Extru-seq results from MSCs and HEK293T cells. For the *PCSK9* and *Albumin* sgRNAs, respectively, 30% and 70% of the top 10 predicted off-target loci (with the top 10 DNA cleavage scores) from the experiment involving MSCs were also among the top 10 in the experiment with HEK293T cells (Additional File 1: Fig. S17). These results indicate that the genome-wide off-target loci predicted by Extru-seq differ by cell type. When the intersections of the Venn diagrams were analyzed using the normalized rank sum test (Additional File 1: Fig. S18), a high *p*-value was observed for the *Albumin* results whereas a low *p*-value was observed for the *PCSK9* results. It appears that the difference in cell type may or may not change the off-target rank distribution.

## Discussion

Cell-based methods like GUIDE-seq are known to miss more valid off-target candidates than in vitro and in silico methods. We found that the miss rate of Extru-seq (2.33%) was 12.6-fold less than that of GUIDE-seq (29.5%). In addition, like other in vitro methods, Extru-seq can be universally applied to various cell types of different origins, because there is no need for insertion of dsODNs into DSB sites in living cells, as is required for GUIDE-seq. Because Extru-seq overcame key limitations of both cell-based methods (high miss rates and a need for optimization for different cell types) and in vitro methods (low validation rates and a loss of cell-type specific information, as evidenced by the low *p*-values obtained when results from in vitro and cell-based methods are compared), we suggest that Extru-seq is a strong candidate as a balanced method for obtaining a comprehensive list of off-target sites in various cell types and patient-specific clinical safety tests. Finally, the strong performance of the DNA cleavage score of Extru-seq as the binary classifier of the validation results was supported by the highest value of area under ROC curves for Extru-seq when compared to the other methods.

Most of the cell-based methods use “surrogate” cell lines to predict genome-wide off-target sites for human clinical samples. However, a difference might exist in the chromatin and epigenetic status of dividing cell lines in vitro and that of most non-dividing cells in vivo, as was the case when we compared Extru-seq results from HEK293T cells and MSCs. Therefore, it is suggested that Extru-seq should be performed with clinically more relevant cells instead of surrogate cell lines. Recently, two cell-based methods (DISCOVER-seq [4] and GUIDE-tag [3]) were performed directly in mouse models such that cell type-specific predictions could be made in vivo. However, for preclinical IND studies for human therapeutics, performing these methods directly in human organs is unlikely. Rather, Extru-seq could be performed directly with primary human cells that had been isolated from specific patients or organs. Because the current Extru-seq protocol involves WGS, performing large number of Extru-seq would be costly. This problem could be avoided if the PCR-based amplification protocol from SITE-seq [15] was applied to Extru-seq. In addition, the Digenome-seq analysis algorithm missed a few bona fide off-target sites that were confirmed manually. Optimization of the algorithm for Extru-seq may increase the sensitivity of the overall analysis. The size of the extruder could also be minimized to reduce the cost and increase the throughput of Extru-seq. In addition, it is difficult to detect Cas9-mediated large deletions, chromosomal depletions, and translocations using Extru-seq. To address this problem, recently developed tools such as CAST-seq [36] could be used in combination with Extru-seq.

## Conclusions

In this study, we developed Extru-seq, a novel cell-based in vitro genome-wide off-target prediction method, and compared its performance with that of three different genome-wide off-target prediction methods (GUIDE-seq, Digenome-seq, and an in silico prediction method). Using promiscuous guide sequences in human cells and mouse models, we showed that each method predicted different sets of off-target sites. When we examined whether the top 10 candidates from each method could be validated, Extru-seq showed validation rate and area under ROC curve (92.5% and 0.83) comparable with that of GUIDE-seq (97.5% and 0.81), values that were both higher than the values for Digenome-seq (45% and 0.72) or the in silico methods [(62.5% and 0.69) for CROP and (67.5% and 0.68) for CFD]. In addition, we performed normalized rank sum tests with the populations in the intersections of Venn diagrams of results from the different methods (the first time, to our knowledge, that this analysis has been performed), which led to the conclusion that the score distributions of each method are dissimilar except for the those from the GUIDE-seq:Extru-seq and DIG-seq:Digenome-seq pairs (*n* ≥ 3). The results indicate that Extru-seq shows a strong resemblance to the cell-based GUIDE-seq method, whereas DIG-seq shows a strong similarity with the in vitro Digenome-seq method.

## Methods

### Design of promiscuous sgRNAs

Candidate target sequences containing an NGG protospacer adjacent motif in the *PCSK9* and *Albumin* genes in the mouse (mm10) genome were extracted using Cas-Designer [37]. The extracted sequences were aligned to the human genome (hg19) and only the aligned sequences with no mismatches were selected. The selected candidates were analyzed with Cas-OFFinder [18] and those with a diverse set of related sequences that contained different numbers of mismatches (ranging from 0 to 5 per site) and that were the most broadly distributed throughout the human and mouse genomes were chosen as targets.

### Construction of plasmids for sgRNA and Cas9 expression

The *Streptococcus pyogenes* Cas9 sequence [38] and the designed promiscuous sgRNA sequences that target *Albumin* and *PCSK9* were cloned into the AAV plasmid backbone used in a previous study [39] to create Cas9 (pAAV-Cas9) and sgRNA (pAAV-Albumin and pAAV-PCSK9) expression vectors. Cas9 expression is under the control of the CMV promoter and sgRNA expression is under the control of the U6 promoter. Guide sequences targeting *FANCF*, *VEGFA*, and *HBB* genes [14] were cloned into pRG2 vector (Addgene #104174).

### GUIDE-seq

Human HEK293T cells and mouse NIH-3T3 cells were maintained in Dulbecco’s modified Eagle medium (DMEM) with 10% fetal bovine serum (FBS) (ATCC, HEK293T, CRL-11268; NIH-3T3, CRL-1658) and 1% penicillin-streptomycin at 37°C in the presence of 5% CO_2_. HEK293T and NIH-3T3 cells were subcultured every 72 h to maintain 80% confluency. For GUIDE-seq, 2x10^5^ HEK293T cells were transfected with plasmids expressing sgRNA (500 ng, pAAV-Albumin or pAAV-PCSK9) and Cas9 (500 ng, p3s-Cas9HC; Addgene plasmid #43945) and 5 pmol dsODN using Lipofectamine 2000. 2 × 10^5^ NIH-3T3 cells were transfected with plasmids expressing sgRNA (250 ng, pAAV-Albumin or pAAV-PCSK) and Cas9 (500 ng, p3s-Cas9HC; Addgene plasmid #43945) and 100 pmol dsODN using an Amaxa P3 electroporation kit (V4XP-3032; program EN-158). Transfected cells were transferred to a 24-well plate containing DMEM (1 mL/well) that had been pre-incubated at 37°C. After 72 h, genomic DNA was isolated using a QIAamp DNA Mini Kit (Qiagen).

1000 ng of purified DNA was fragmented using a Covaris system (Duty Factor: 10%, PIP: 50, Cycles per burst: 200, Time: 50 s, Temperature: 20 °C) and purified using Ampure XP beads (A63881). Sequencing libraries were generated from the DNA using an NEBNext® Ultra™ II DNA Library Prep Kit for Illumina (E7546L) per the manufacturer’s protocol. Next, the regions of the library containing dsODN sequences were amplified using dsODN-specific primers and sequenced using Miseq (Illumina, TruSeq HT Kit). The remaining procedures were as described previously [2]. For data analysis, GUIDE-seq (1.0.2; https://pypi.org/project/guide-seq/) was used, which is compatible with Python 3.

### Construction of plasmids for sgRNA transcription and in vitro transcription reactions

To improve the yield and accuracy of sgRNA transcription, we modified a previously described method [40]. Briefly, sgRNA templates were generated by annealing two complementary oligonucleotides followed by PCR amplification. BamHI, BsaI, and KpnI restriction sites were attached to the ends of sgRNA templates with a second PCR. Tailed sgRNA templates were inserted into the pUC19 plasmid digested with BamHI and KpnI. sgRNA-encoding plasmids were linearized with BsaI, which resulted in proper sgRNA end sequences. Linearized plasmids were incubated with 7.5U/μl T7 RNA polymerase (NEB, M0251L) in reaction buffer (NEB, B9012S) containing 14 mM MgCl_2_ (NEB, B0510A), 10mM DTT (Sigma, 43816), 0.02U/μl yeast inorganic pyrophosphatase (NEB, M2403L), 1U/μl murine RNase inhibitor (NEB, M0314L), 4mM ATP (NEB, N0451AA), 4mM GTP (NEB, N0452AA), 4mM UTP (NEB, N0453AA), and 4mM CTP (NEB, N0454AA) for 8 h at 37 °C. Yeast inorganic phosphatase was included to enhance sgRNA synthesis. After the reaction, the mixture was mixed and incubated with DNase I to remove the DNA template; transcribed sgRNAs were then purified using a PCR purification kit (Favorgen, #FAGCK001-1).

### Digenome-seq

Genomic DNA from HEK293T and NIH-3T3 cells was purified with a DNeasy Blood & Tissue Kit (Qiagen). Both types of genomic DNA (10 μg) were incubated with Cas9 protein (10 μg) and sgRNAs targeting *Albumin* and *PCSK9* (10 μg each) in a 1-mL reaction volume containing NEB3 buffer [100 mM NaCl, 50 mM Tris-HCl, 10 mM MgCl_2_, 100 μg/mL bovine serum albumin (BSA), at pH 7.9] for 8 h at 37°C. Digested genomic DNA was then treated with RNase A (50 μg/mL, Qiagen) for 10 min to degrade sgRNAs and purified with a DNeasy Blood & Tissue Kit (Qiagen) again.

Genomic DNA (1 μg) was fragmented to the 300-bp range using a Covaris system (Life Technologies) and blunt-ended using End Repair Mix (Thermo Fischer). Fragmented DNA was ligated with adapters to produce libraries, which were then subjected to WGS using a HiSeq X Ten Sequencer (Illumina) at Macrogen. WGS was performed at a sequencing depth of 30–40×. DNA cleavage sites were identified using the Digenome 1.0 program [41].

### In silico prediction of off-target sites

Genome-wide candidate off-target sites with fewer than seven nucleotide mismatches with the chosen sgRNAs were obtained using Cas-OFFinder (hg19). CROP scores (heuristic scores that indicate if the candidate off-target sites would be edited) were computed using the CROP prediction model and optimized parameters (https://github.com/vaprilyanto/crop) based on a previous paper [31]. CFD scores (percent activity values provided in a matrix of penalties based on mismatches of each possible type at each position within the guide RNA sequence) were calculated using “crisprScore” R package [32]. For both calculations, GX19 (GACATGCATATGTATGTGTG for *Albumin* and GAGGTGGGAAACTGAGGCTT for *PCSK9*) sgRNA sequences and X20 target sequences were used.

### Extru-seq

In preparation for Extru-seq, the transcribed sgRNAs were refolded in 1X NEBuffer 3.1 reaction buffer (100 mM NaCl, 50 mM Tris-HCl, 10 mM MgCl_2_, 100 μg/mL BSA, at pH 7.9). sgRNAs were heated to 98 °C for 2 min, after which the temperature was lowered at a rate of 0.1 °C/s until 20 °C was reached. To reduce reaction inhibition from a high concentration of glycerol, Cas9 buffer (10 mM Tris-HCl, 0.15 M NaCl, 50% glycerol, at pH 7.4) was exchanged with elution buffer (100 mM NaCl, 50 mM Tris-HCl, 10 mM MgCl_2_, at pH 8.0). Buffer exchange was conducted through a 10K Amicon® Ultra-15 Centrifugal Filter (Millipore).

HEK293T and NIH-3T3 cells were harvested with 0.25% trypsin-EDTA and human bone marrow MSCs (BM-MSCs) were harvested with 0.05% trypsin-EDTA. Harvested cells were resuspended in Dulbecco’s phosphate-buffered saline (PBS). Buffer-exchanged Cas9 (800 mg) and refolded sgRNA (530 μg) were preincubated for 10 min at room temperature to form RNP complexes. (For multiplex Extru-seq, buffer-exchanged Cas9 (800 mg) and five different refolded sgRNAs (106 μg each) were used). 1 × 10^7^ cells were mixed with 5000 nM RNP complexes in 1 mL 1X NEBuffer 3.1 reaction buffer (100 mM NaCl, 50 mM Tris-HCl, 10 mM MgCl_2_, 100 μg/mL BSA, at pH 7.9). To perform Extru-seq in the presence of SCR7, SCR7 pyrazine (Sigma, SML1546) was added (1 μM). After gentle pipetting, suspended cells were extruded 11 times through an 8-μm pore-sized polycarbonate membrane filter (Whatman) using a mini-extruder (Avanti Polar Lipids). The extruded sample was then incubated at 37°C for 16 h. Genomic DNA was purified from the extruded sample using a FavorPrep Blood Genomic DNA Extraction Mini Kit (Favorgen, #FAGCK001-2) after RNase A (2 mg/mL) was added to remove sgRNA and RNA. WGS was carried out at a sequencing depth of 30–40×. DNA cleavage sites were identified using the Digenome-seq standalone program (http://www.rgenome.net/digenome-js/standalone). Analysis filtering options were as follows: minimum depth, 10, minimum score, 0.05, and minimum ratio, 0.01; other options were the default. [As the developer of a new tool, we checked all the sites identified by Extru-seq with the Integrative Genomics Viewer (IGV). Some of the loci look as if they are false-positive candidates (that is, non-cleavage sites according to IGV (Additional File 4: Table S3)). These false positives were also observed in Digenome-seq. The relevant bam files are available at the NCBI Bioproject (https://www.ncbi.nlm.nih.gov/bioproject/) under accession number PRJNA796642.]

### Assignment of off-target results of Digenome-seq and Extru-seq to CAS-OFFinder results

Unlike GUIDE-seq and CAS-OFFinder, the standalone Digenome-seq program does not have a sgRNA:off-target alignment function that provides information about the number of mismatches and type of bulge (DNA or RNA) between the guide and off-target site. [The web version of the Digenome-seq analysis tool (http://www.rgenome.net/digenome-js/#!) has an optional alignment function with an alignment score that does not provide any information about the number of mismatches or type of bulge.] Instead, we used CAS-OFFinder to identify off-target sites with up to seven mismatches and two bulges relative to the target sequence. The positions of the off-target candidates identified by Digenome-seq and Extru-seq were then compared with those identified by CAS-OFFinder so that the information about mismatches and bulge type from CAS-OFFinder could be assigned to the loci identified by Digenome-seq and Extru-seq.

### Validation of candidate off-target sites using a human cell line

Human HEK293T cells were maintained in DMEM supplemented with 10% FBS (ATCC, CRL-11268) and 1% penicillin-streptomycin at 37°C in the presence of 5% CO_2_. To determine indel frequencies at candidate off-target sites, 2×10^5^ HEK293T cells were transfected with plasmids expressing sgRNA (500 ng, pAAV-Albumin, pAAV-PCSK9, pRG2-HBB, pRG2-FANCF, or pRG2-VEGFA) and Cas9 (500 ng, pAAV-Cas9 or p3s-Cas9HC; Addgene plasmid #43945) using Lipofectamine 2000 (vendor, amount). The cells were incubated at 37°C for 3 days, after which genomic DNA was prepared using a FavorPrep Blood Genomic DNA Extraction Mini Kit (Favorgen, #FAGCK001-2). The deep sequencing data are available at the NCBI Bioproject (https://www.ncbi.nlm.nih.gov/bioproject/) under accession number PRJNA796642. We used the following criteria used by EDITAS Medicine [23] to determine whether the target was validated or false (Additional File 5: Table S4). First, the indel of the sample must be higher than 0.1% for the sample to be validated. Second, the treated/control ratio must be higher than 2.

### AAV production

AAV8 carrying the desired cloned sequences (pAAV-PCSK9, pAAV-Albumin, and pAAV-Cas9) were produced by VigeneBioscience at large scale [10^13^ genome copies (GC)/mL]. The resulting AAVs were aliquoted and stored at −70 °C until use.

### Animal studies

All animal experiments were approved by the Institutional Animal Care and Use Committee (IACUC) of Yonsei University College of Medicine (IACUC number 2019-0215). C57BL/6 mice were maintained under a 12:12 h light-dark cycle.

### AAV injection

Two forms of AAV8, respectively carrying pAAV-Cas9 and one of the two pAAV-sgRNAs (pAAV-PCSK9 or pAAV-Albumin), were delivered into C57BL/6 mice by systemic (intravenous) and subretinal injection. Both types of injections were performed at a 1:1 GC (pAAV-Cas9:pAAV-sgRNA) ratio. Each dose consisted of 2.5 × 10^11^ GC/animal for intravenous injections and 1.5 × 10^10^ GC/eye for subretinal injections.

For systemic injections, 7- to 9-week-old male mice received a 200-μl tail vein injection with a dose of 2.5 × 10^11^ GC of AAV8 diluted in PBS.

For subretinal injections, 7- to 9-week-old male mice were selected. Under general anesthesia, one pupil per mouse was dilated with an eye drop containing tropicamide and phenylephrine. The body temperature of the mice was maintained at 37°C with a heating pad during the experiment. A small incision was made with a 1/2 30G needle 1 mm from the limbus of the cornea. A Hamilton syringe with a 33G blunt needle, loaded with 2 μl of solution containing the AAV8 mixture, was inserted through the incision until the point at which resistance was felt (subretinal space). To prevent unnecessary tissue damage, we carefully and gently injected the volume, waited for 20–30 s to allow it to spread evenly, and then slowly removed the syringe. Antibiotic ointment was then applied to the surface of the eyeball. Four mice were used for each injection method and each sgRNA.

### DNA preparation from harvested organs and tissues

Organs and tissues were harvested 2 weeks and 3 months after the injection. Animals were euthanized by cardiac puncture under isoflurane anesthesia at the experimental endpoint. The organs—including the eye, liver, spleen, lung, kidney, muscle, brain, and testis—were dissected, snap-frozen in liquid nitrogen, and stored at −70°C until further analyses.

In the case of the subretinal injections, the neural retina and retinal pigment epithelium (RPE) were separated and prepared. The cornea, iris, lens, and vitreous were removed from the enucleated eyeball. The remaining eye tissues were incubated in hyaluronidase solution at 37 °C, 5% CO_2_ for 45 min, and then incubated in cold PBS for 30 min to inactivate the hyaluronidase activity. Next, the eye tissue was transferred to fresh PBS and the neural retina was gently separated from the retina/RPE/choroid/sclera complex. The remaining RPE/choroid/sclera complexes were incubated in trypsin solution at 37 °C, 5% CO_2_ for 45 min, and gently shaken until the RPE sheets were fully detached. All separated RPE sheets and RPE cells were collected. The genomic DNA was extracted using a DNeasy Blood & Tissue Kit (Qiagen, Cat No. 69506) according to the manufacturer’s instructions.

### Targeted deep sequencing

Genomic DNA from mouse RPE cells was amplified with a REPLI-g Single Cell Kit (Qiagen) according to the manufacturer’s protocol.

Target sites and potential off-target sites were analyzed by targeted deep sequencing. Deep sequencing libraries were generated by PCR. TruSeq HT Dual Index primers were used to label each sample. Pooled libraries were subjected to paired-end sequencing using MiSeq (Illumina).

### Statistical analysis

Scores/sequence read counts were min-max normalized. In each population, the maximum value was normalized to 1 and the minimum value was normalized to 0. Wilcoxon Rank-Sum Test was performed on the samples in each intersection of Venn diagram to test the equality of score medians from two different groups. Results from the two-sided unpaired Mann-Whitney test calculated by Prism (version 9.4.1) are shown.

Authors’ contributions

J.K.L., S.B., J-S.K., Y.K., J.L., J.K.H., and J.E. supervised the research. J.K., M.K., A.J., G.H.H., M.J., and G.C. performed the experiments. W.H., U.K., and H.K. analyzed the data. The authors read and approved the final manuscript.

## Supplementary Information

**Additional file 1: Fig. S1.** Genome editing drugs and off-target prediction methods used in IND studies. **Fig. S2.** Optimization of Extru-seq conditions. **Fig. S3.** Extru-seq WGS data analyzed using IGV to reveal cleavage patterns. **Fig. S4.** Genome-wide off-target loci containing zero to six mismatches relative to the target. **Fig. S5.** Part of the GUIDE-seq results obtained from HEK293T cells. **Fig. S6.** Manhattan plot of Digenome-seq results obtained from HEK293T cells. **Fig. S7.** Manhattan plot of Extru-seq results obtained from HEK293T cells. **Fig. S8.** Box and whisker plots showing results from different off-target prediction methods for promiscuous sgRNAs targeting *PSK9* and *Albumin*. **Fig. S9.** Indel ratios calculated following analysis of genomic DNA obtained from organs from C57BL/6 mice injected with two AAV8 vectors, respectively expressing Cas9 and sgRNA targeting either *PCSK9* or *Albumin*. **Fig. S10.** Validation results from targeted deep sequencing of the top 10 predicted off-target sites. **Fig. S11.** GUIDE-seq results obtained from HeLa cells. **Fig. S12.** Manhattan plot of Extru-seq results obtained from HeLa cells. **Fig. S13.** Validation results from targeted deep sequencing of the top 10 predicted off-target sites. **Fig. S14.** Number of samples found in the intersections of Venn diagrams showing the overlap between off-target sites predicted by different methods. **Fig. S15.** Off-target sites, predicted by Extru-seq or GUIDE-seq and validated by deep sequencing. **Fig. S16.** Venn diagrams showing the number of predicted off-target sites. **Fig. S17.** The top 10 potential off-target loci predicted by Extru-seq for MSCs and HEK293T cells. **Fig. S18.** p-values obtained by the normalized rank sum test for each pair of off-target prediction methods [42–45].

**Additional file 2: Table S1.** Percentage of shared loci among the top 10 candidate off-target loci tabulated for each prediction method.

**Additional file 3: Table S2.** Manually validated off-target sites from Extru-seq WGS data visualized using IGV.

**Additional file 4: Table S3.** Manually excluded false positive off-target sites from Digenome-seq and Extru-seq WGS data visualized using IGV.

**Additional file 5: Table S4.** Targeted deep sequencing data for off-target validation.

**Additional file 6.** Review history.

## Data Availability

We have submitted the deep sequencing at the NCBI Bioproject (https://www.ncbi.nlm.nih.gov/bioproject/) under accession number PRJNA796642 [46].
