# Supplementary material for: Extru-seq: a method for predicting genome-wide Cas9 off-target sites with advantages of both cell-based and in vitro approaches
Source: Genome Biol. 2023 Jan 10;24:4. doi: 10.1186/s13059-022-02842-4 (PMC9832775; doi:10.1186/s13059-022-02842-4)
Supplement: Supplementary file 1 — Additional file 1: Fig. S1. Genome editing drugs and off-target prediction methods used in IND studies. Fig. S2. Optimization of Extru-seq conditions. Fig. S3. Extru-seq WGS data analyzed using IGV to reveal cleavage patterns. Fig. S4. Genome-wide off-target loci containing zero to six mismatches relative to the target. Fig. S5. Part of the GUIDE-seq results obtained from HEK293T cells. Fig. S6. Manhattan plot of Digenome-seq results obtained from HEK293T cells. Fig. S7. Manhattan plot of Extru-seq results obtained from HEK293T cells. Fig. S8. Box and whisker plots showing results from different off-target prediction methods for promiscuous sgRNAs targeting PSK9 and Albumin. Fig. S9. Indel ratios calculated following analysis of genomic DNA obtained from organs from C57BL/6 mice injected with two AAV8 vectors, respectively expressing Cas9 and sgRNA targeting either PCSK9 or Albumin. Fig. S10. Validation results from targeted deep sequencing of the top 10 predicted off-target sites. Fig. S11. GUIDE-seq results obtained from HeLa cells. Fig. S12. Manhattan plot of Extru-seq results obtained from HeLa cells. Fig. S13. Validation results from targeted deep sequencing of the top 10 predicted off-target sites. Fig. S14. Number of samples found in the intersections of Venn diagrams showing the overlap between off-target sites predicted by different methods. Fig. S15. Off-target sites, predicted by Extru-seq or GUIDE-seq and validated by deep sequencing. Fig. S16. Venn diagrams showing the number of predicted off-target sites. Fig. S17. The top 10 potential off-target loci predicted by Extru-seq for MSCs and HEK293T cells. Fig. S18. p-values obtained by the normalized rank sum test for each pair of off-target prediction methods [42–45]. [file 13059_2022_2842_MOESM1_ESM.docx]

Supplementary Information

**Extru-seq: a method for predicting genome-wide Cas9 off-target sites with advantages of both cell-based and in vitro approaches**

**Jeonghun Kwon^1,^** ^†^**, Minyoung Kim^1,^**^†^ **, Woochang Hwang^2,3,^**^†^ **, Anna Jo^1^, Gue-Ho Hwang^4^, Minhee Jung^1^, Un Gi Kim^1^, Gang Cui^5^, Heonseok Kim^6^, Joon-Ho Eom^7^, Junho K. Hur^3,8^, Junwon Lee^5^, Youngho Kim^1^, Jin-soo Kim^9^, Sangsu Bae^10^, Jungjoon K. Lee^1,*^**

^†^These authors contributed equally to this work. *Correspondence: jj.lee@toolgen.com

| **Name** | **Genome editor** | **Target gene** | **Company** | **Clinical phase** | **Disease** | **Off-target prediction methods used** | **Number of methods used** | **Reference** |
| --- | --- | --- | --- | --- | --- | --- | --- | --- |
| Universal CAR T-Cells | TALEN | *TRAC*/*CD52* | Cellectis | Phase 2 | Cancer | *in silico* (TAL Effector-Nucleotide Targeter (TALE-NT) 2.0) | 1 | [24] |
| PBCAR0191 | I-CreI | *TRAC* | Precision Bioscience | Phase 1/2 | Cancer | *in silico* (COSMID) | 1 | [25] |
| SB-728 | Zinc Finger | *CCR5* | Sangamo | Phase 1/2 | HIV | Cell-based (integration deficient lentivirus end-capture-based integration site analysis), *in vitro* (SELEX-based oligo capture) | 2 | [42] |
| CTX110, CTX120 | CRISPR-Cas9 | *TRAC* | CRISPR Therapeutics | Phase 1/2 | Cancer | NA | NA | [43] |
| NY-ESO-1 directed Cell | CRISPR-Cas9 | *TRAC*, *PCDC1*, *TRBC* | UPenn | Phase 1 | Cancer | Cell-based (GUIDE-seq) | 1 | [26] |
| LBP-EC01 | Cas3 | Bacterial genomic DNA-specific sequence | Locus Bioscience | Phase 1b | Urinary tract infection | NA | NA | [44] |
| EDIT 101 | CRISPR-Cas9 | *CEP290* | EDITAS Medicine | Phase 1/2 | LCA10 | Cell-based (GUIDE-seq), *in vitro* (Digenome-seq), *in silico* (Cas-OFFinder) | 3 | [23] |
| SB Therapeutics | Zinc Finger | *Albumin* | Sangamo | Phase 1/2 | MPS I, MPS II | Cell-based (Unbiased off-target assessment via AAV integration site analysis), *in vitro* (SELEX-based oligo capture) | 2 | [45] |
| CTX001 | CRISPR-Cas9 | *BCL11a* | CRISPR Therapeutics | Phase 1/2 | Sickle cell disease | Cell-based (GUIDE-seq), *in silico* (NA) | 2 | [35] |
| NTLA-2001 | CRISPR-Cas9 | *TTR* | Intellia | Phase 1 | Hereditary transthyretin amyloidosis with polyneuropathy | Cell-based (GUIDE-seq), *in vitro* (SITE-seq), *in silico* (Cas-OFFinder) | 3 | [22] |

Fig. S1. Genome editing drugs and off-target prediction methods used in IND studies. TALEN: Transcription activator-like effector nuclease; NA: Not available; LCA10, Leber congenital amaurosis 10; MPS, Mucopolysaccharidosis.

(a)


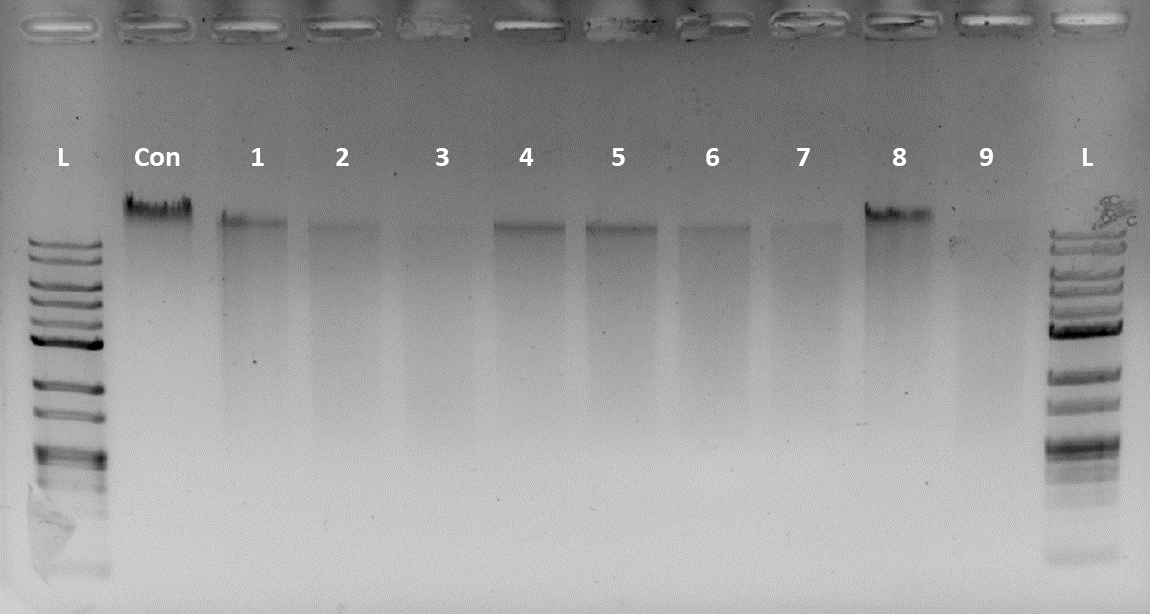


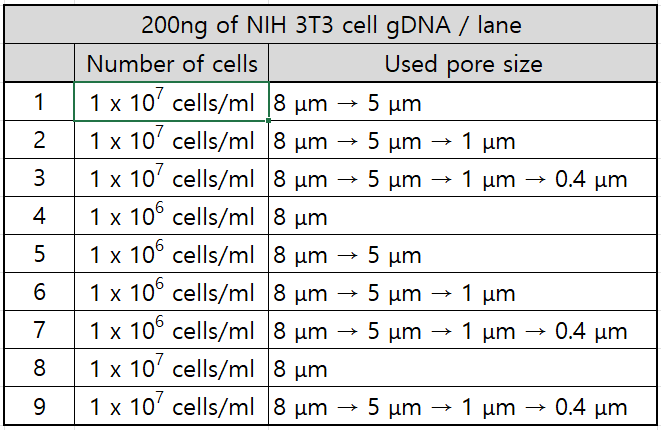


(b)

(c)

(d)

(e)

Fig. S2. Optimization of Extru-seq conditions. (a) The quality of the genomic DNA after an overnight incubation with Cas9 RNPs at 37°C analyzed via gel electrophoresis. Various numbers of NIH-3T3 cells and pore sizes were tested. ‘Con’ indicates control genomic DNA with quality that is high enough for WGS analysis. ‘L’ indicates ladder DNA. (b-e) Cleavage rates measured by quantative PCR (qPCR) for on- and off-target (c-e) sites recognized by the sgRNA targeting the human *PCSK9* site. Rates at the (c) off2, (d) off4, and (e) off7 off-target sites. The horizontal lines represent the mean. Error bars indicate standard deviation (n=3).

(a)


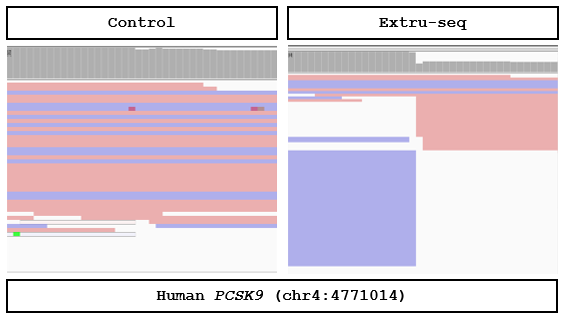


(b)


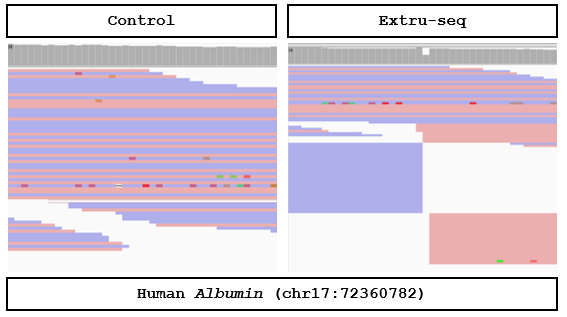


(c)


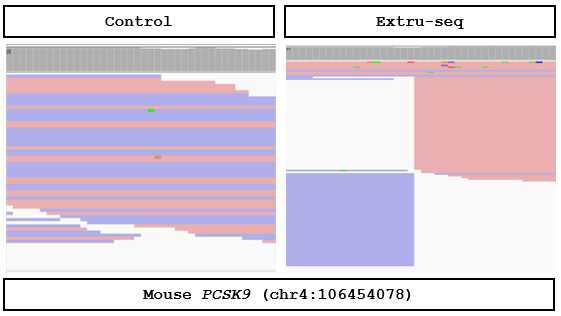


(d)


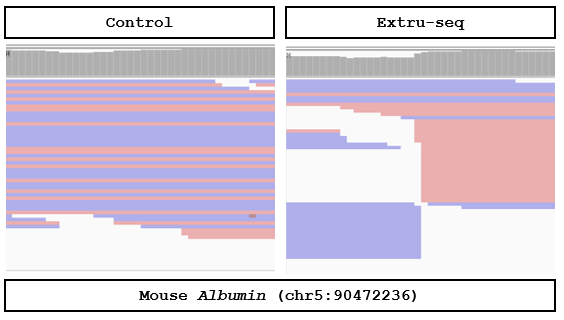


(e)


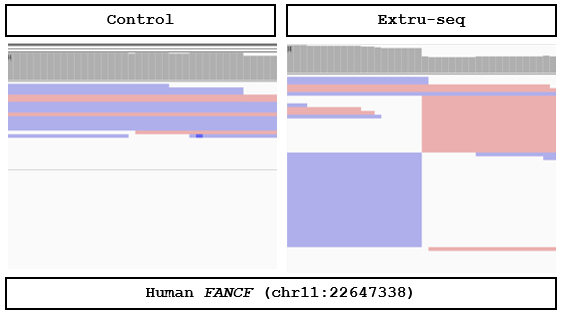


(f)


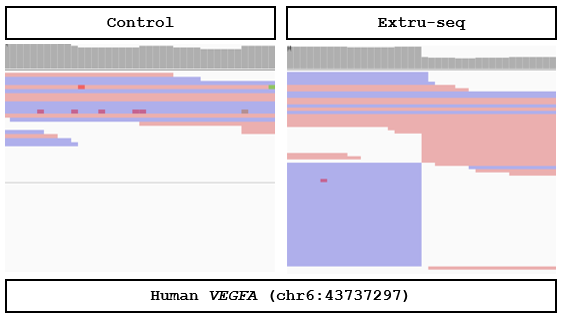


(g)


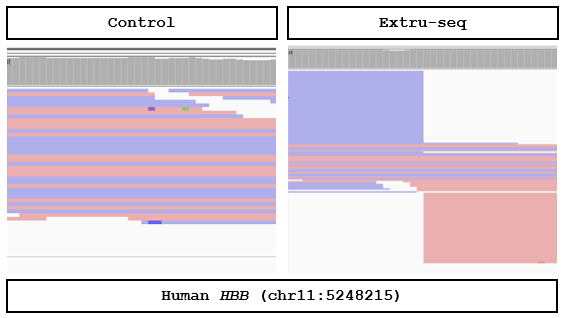


(h)


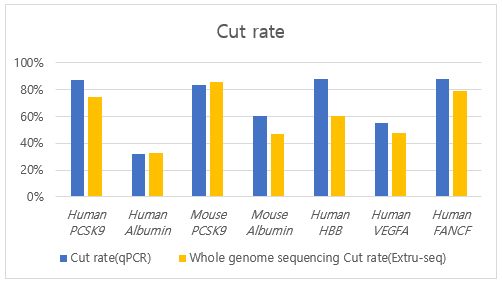


(i)


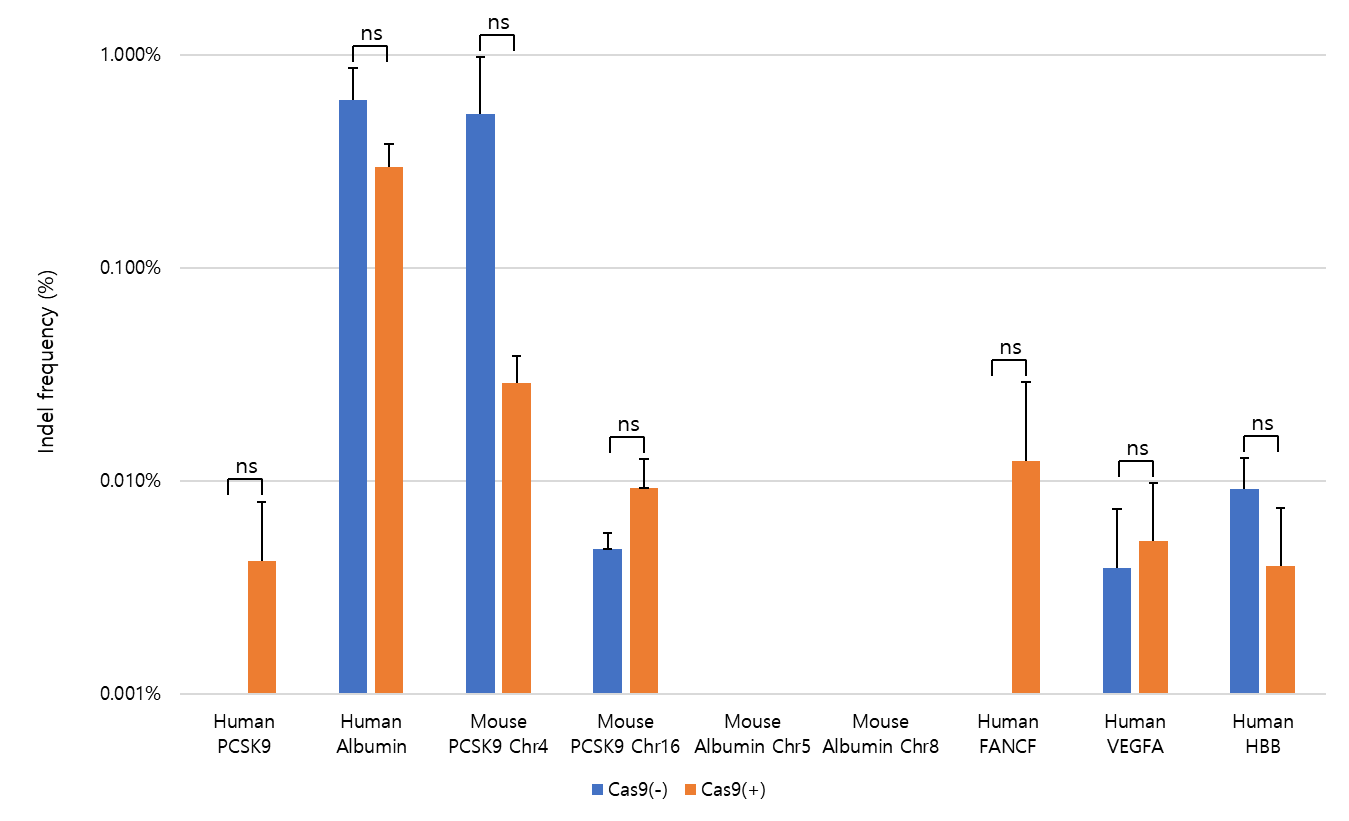


(j)

Fig. S3. Extru-seq WGS data analyzed using IGV to reveal cleavage patterns, for sgRNAs targeting (a) human *PCSK9*, (b) human *Albumin*, (c) mouse *PCSK9*, (d) mouse *Albumin*, (e) human *FANCF*, (f) human *VEGFA*, and (g) human *HBB*. (h) Cleavage rates at the seven on-target sites obtained via qPCR (blue) and manual calculations based on IGV analysis of WGS data (yellow). (i) Indel frequencies measured using targeted deep sequencing data from the Extru-seq samples shown in (a) through (g), with (orange) and without (blue) Cas9 treatment; two-sided unpaired student t-test. Error bars indicate standard deviation. (n = 3). (j) Cleavage rates, % measured using qPCR, at five on-target sites. Results were obtained using multiplex Extru-seq in the absence (left) or presence (right) of 1 μM SCR7. The horizontal lines represent the mean (n=5); two-sided unpaired student t-test.

(a)

| **Target gene** | **Target** | **M0** | **M1** | **M2** | **M3** | **M4** | **M5** | **M6** |
| --- | --- | --- | --- | --- | --- | --- | --- | --- |
| *VEGFA* | GACCCCCTCCACCCCGCCTC | 1 | 0 | 2 | 35 | 446 | 3898 | 17450 |
| *PCSK9* (Previous study) | AGCAGCAGCGGCGGCAACAG | 0 | 1 | 27 | 188 | 821 | 3471 | 16235 |
| *Albumin* (This study) | ACATGCATATGTATGTGTG | 1 | 6 | 42 | 281 | 1740 | 8940 | 43234 |
| *PCSK9* (This study) | AGGTGGGAAACTGAGGCTT | 1 | 5 | 48 | 259 | 1610 | 8985 | 46803 |

(b)

| **Target gene** | **Target** | **M0** | **M1** | **M2** | **M3** | **M4** | **M5** | **M6** |
| --- | --- | --- | --- | --- | --- | --- | --- | --- |
| *VEGFA* | GACCCCCTCCACCCCGCCTC | 1 | 0 | 1 | 23 | 268 | 2048 | 11579 |
| *PCSK9* (Previous study) | AGCAGCAGCGGCGGCAACAG | 1 | 5 | 41 | 354 | 1072 | 3343 | 21891 |
| *Albumin* (This study) | ACATGCATATGTATGTGTG | 4 | 13 | 94 | 684 | 3486 | 16144 | 65390 |
| *PCSK9* (This study) | AGGTGGGAAACTGAGGCTT | 2 | 5 | 22 | 136 | 986 | 6504 | 41972 |

Fig. S4. Genome-wide off-target loci containing zero to six mismatches relative to the target. Off-target sites were predicted by Cas-OFFinder using promiscuous guide sequences targeting *VEGFA* [2], *PCSK9* [30], *Albumin* (this study), and *PCSK9* (this study) with reference genomes (a) hg19 and (b) mm10.

(a)


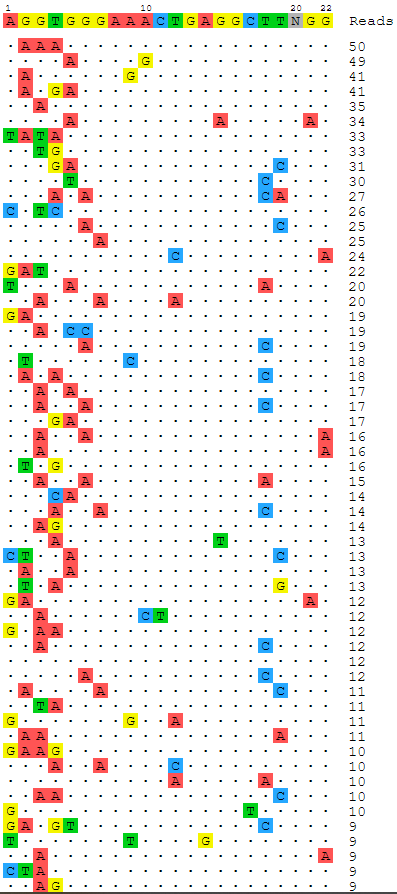


(b)


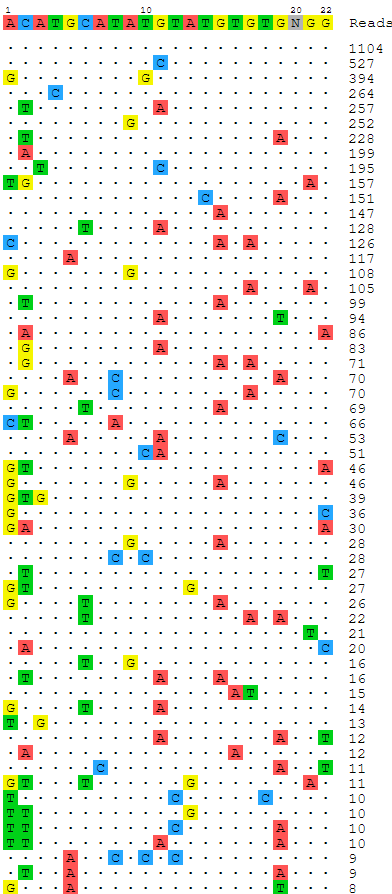


(c)


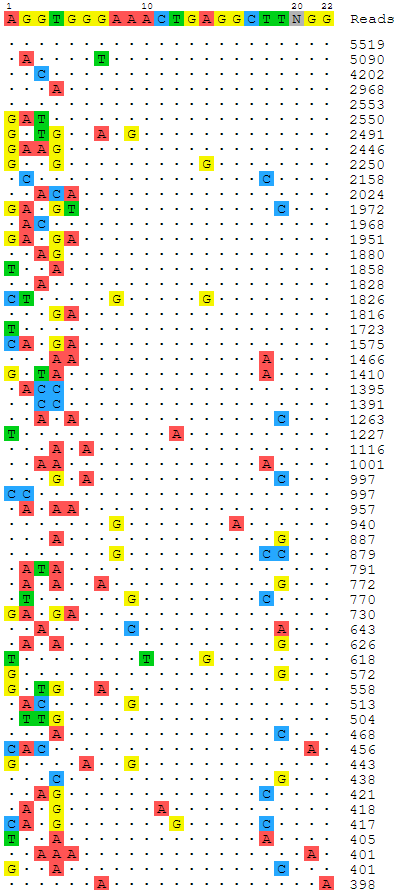


(d)


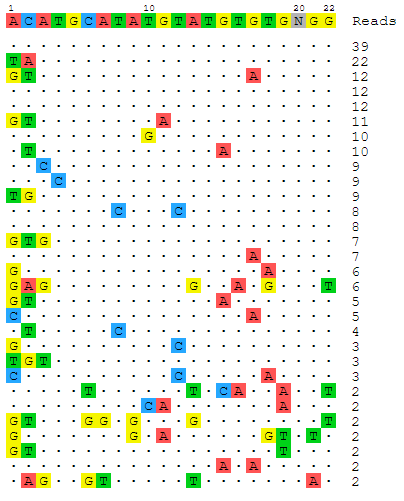


Fig. S5. Part of the GUIDE-seq results obtained from HEK293T cells using sgRNAs targeting (a) *PCSK9* or (b) *Albumin* or from NIH-3T3 cells using sgRNAs targeting (c) *PCSK9* or (d) *Albumin*. Off-target loci with lower ranks were omitted from this graphical representation by the GUIDE-seq analysis program. The omitted loci were included in other subsequent analysis.

(a)


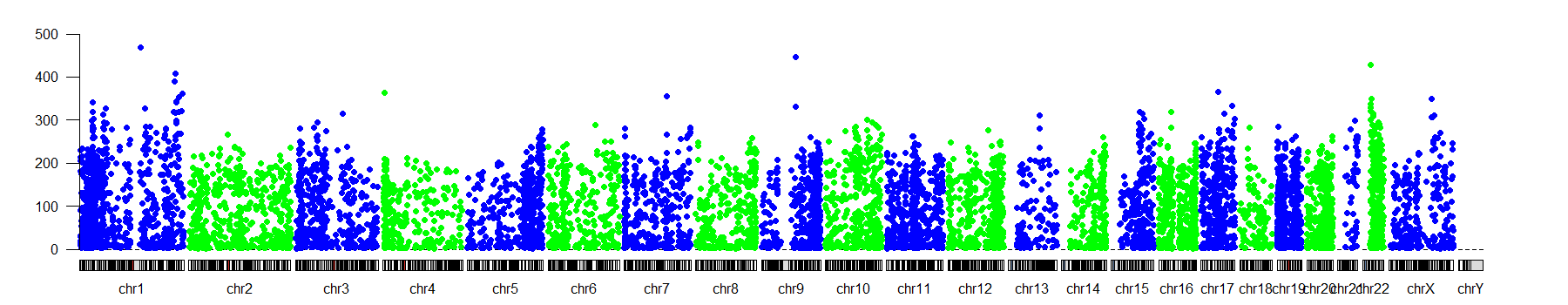


(b)


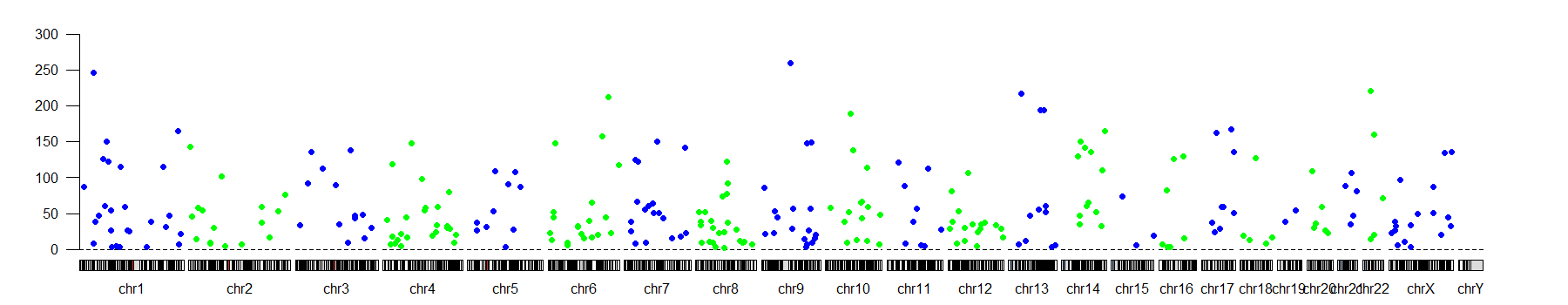


(c)


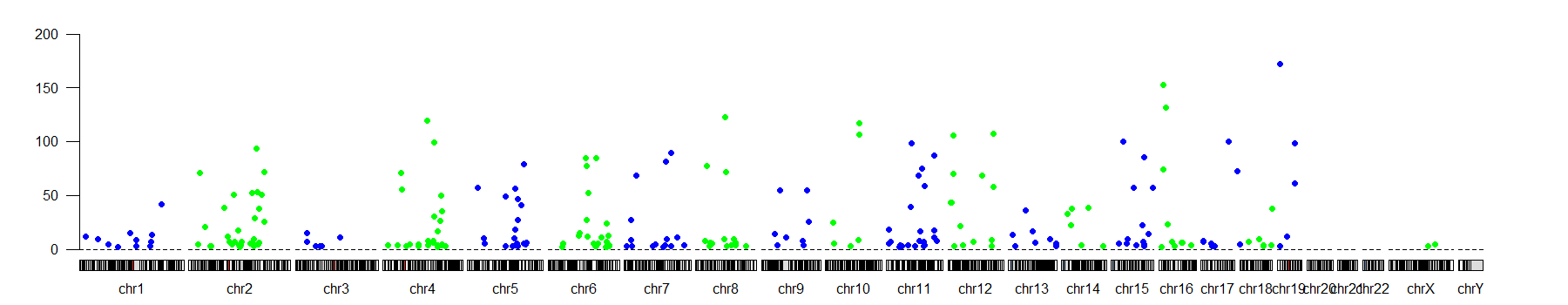


(d)


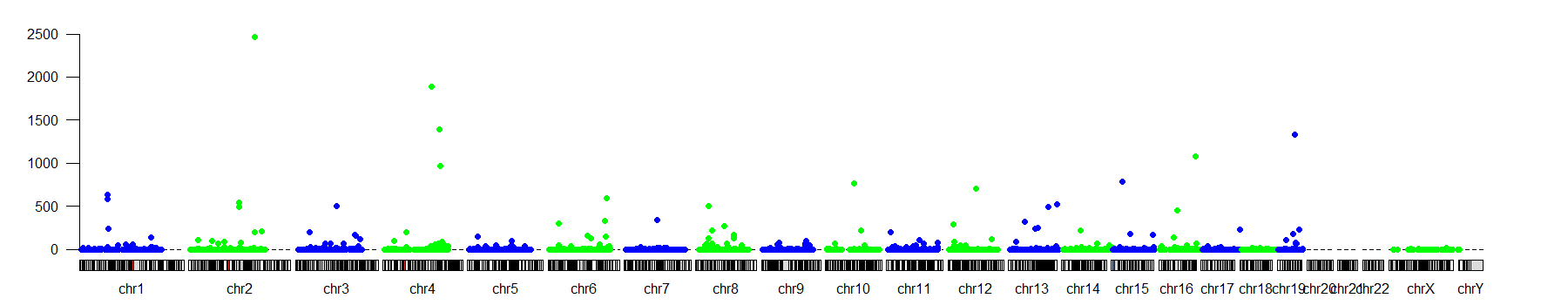


Fig. S6. Manhattan plot of Digenome-seq results obtained from HEK293T cells using sgRNAs targeting (a) *PCSK9* or (b) *Albumin* or from NIH-3T3 cells using sgRNAs targeting (c) *PCSK9* or (d) *Albumin*. The y-axis represents the DNA cleavage score.(a)


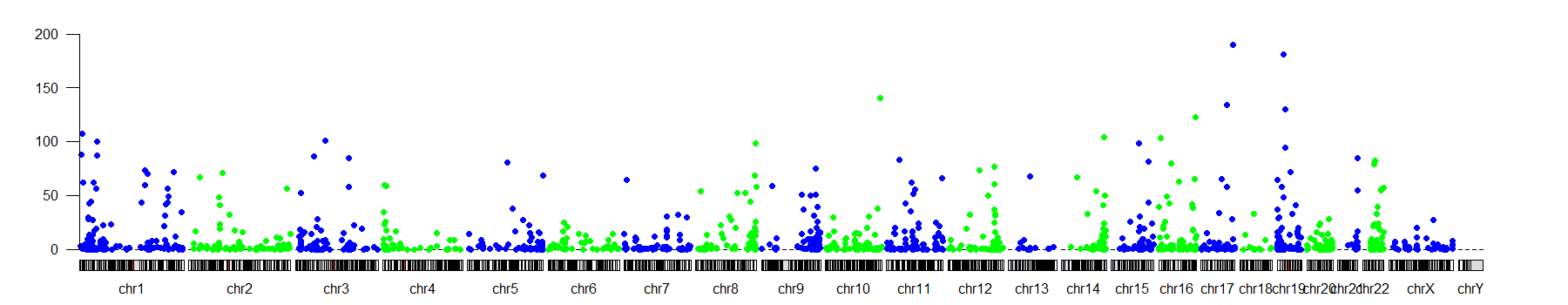


(b)


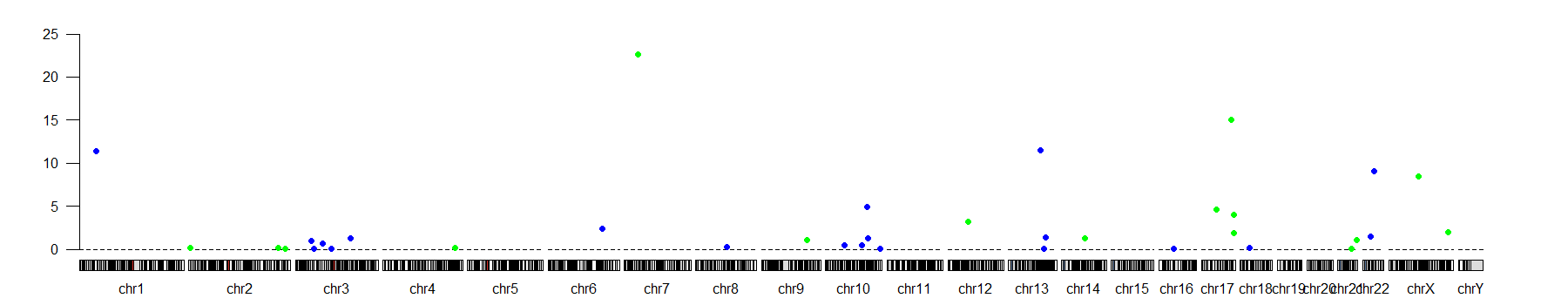


(c)


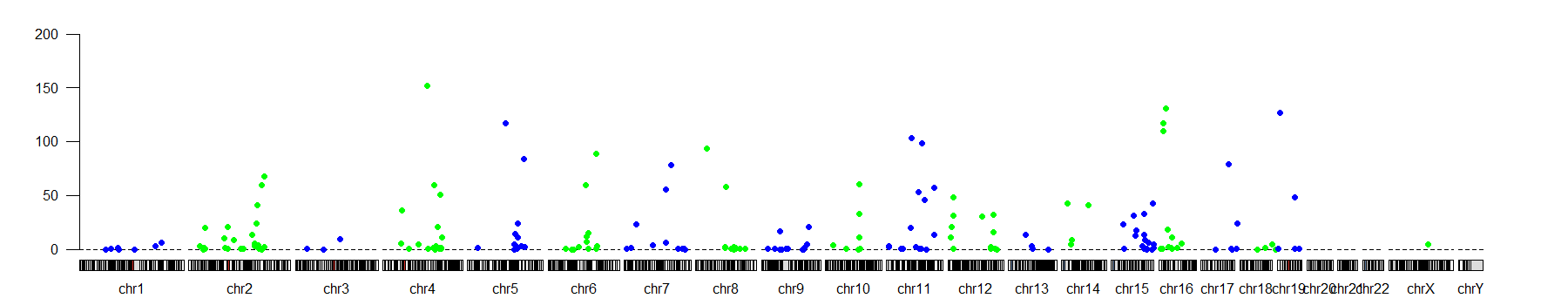


(d)


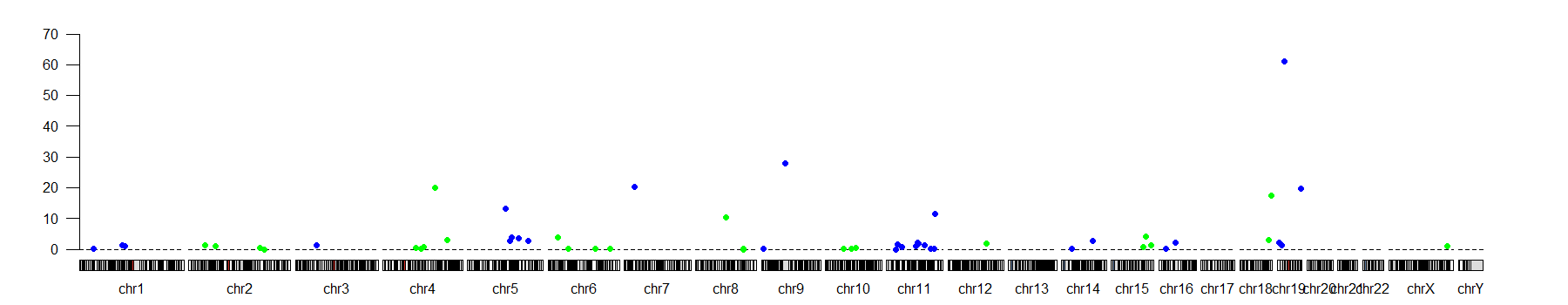


Fig. S7. Manhattan plot of Extru-seq results obtained from HEK293T cells using sgRNAs targeting (a) *PCSK9* or (b) *Albumin* or from NIH-3T3 cells using sgRNAs targeting (c) *PCSK9* or (d) *Albumin*. The y-axis represents the DNA cleavage score.

(a)

|  | Human | Mouse |
| --- | --- | --- |
| *PCSK9* | 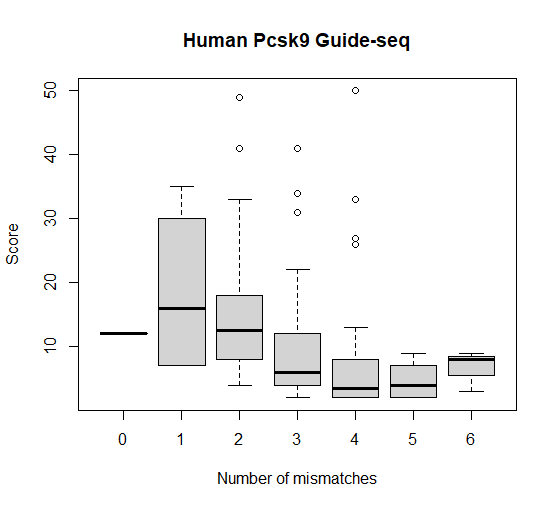 | 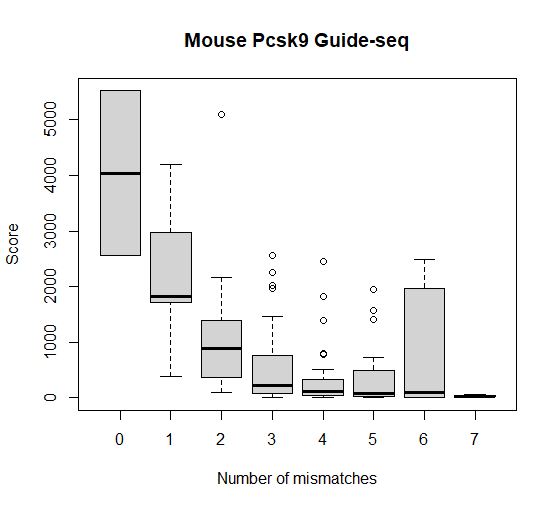 |
| *Albumin* | 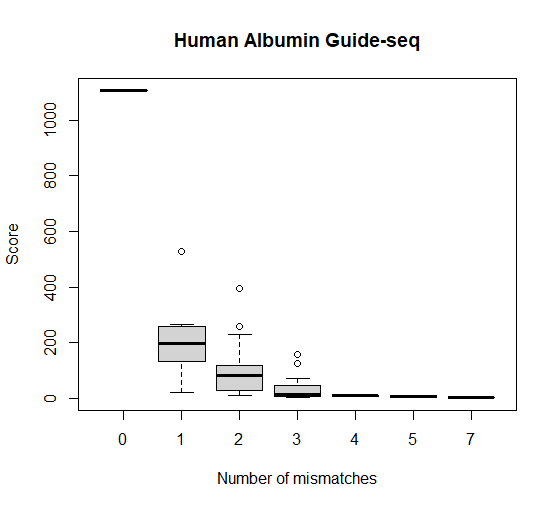 | 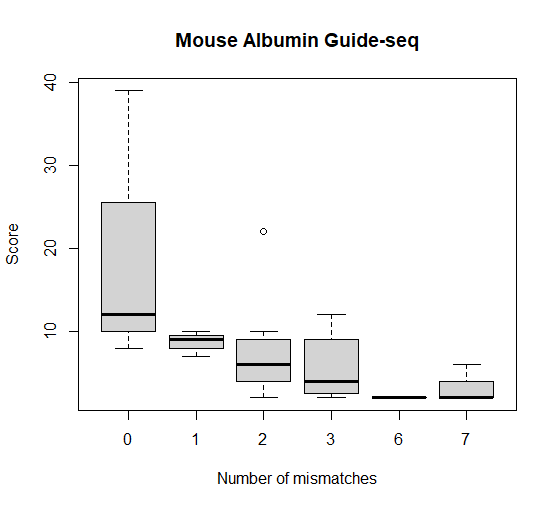 |

(b)

|  | Human | Mouse |
| --- | --- | --- |
| *PCSK9* | 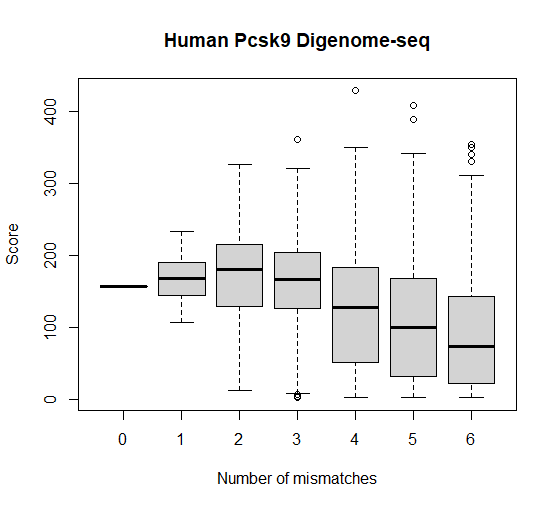 | 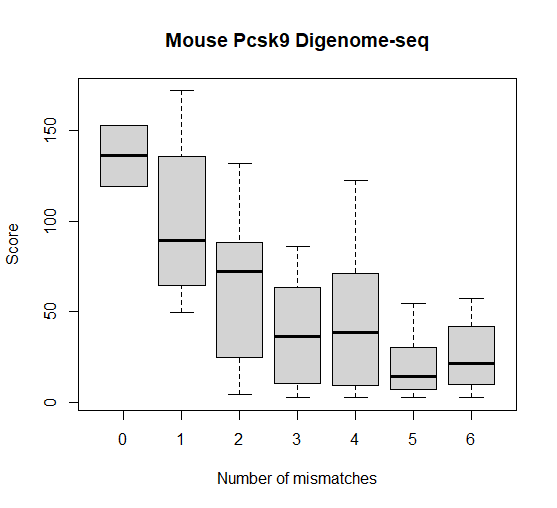 |
| *Albumin* | 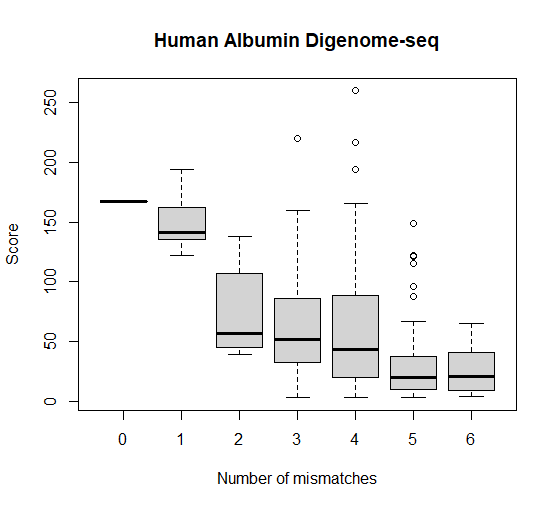 | 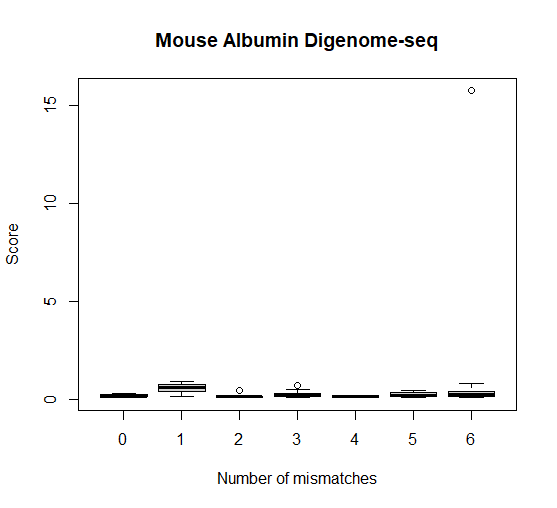 |

(c)

|  | Human | Mouse |
| --- | --- | --- |
| *PCSK9* | 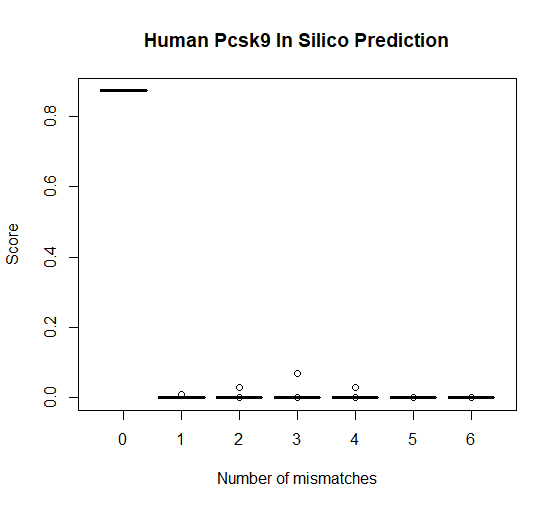 | 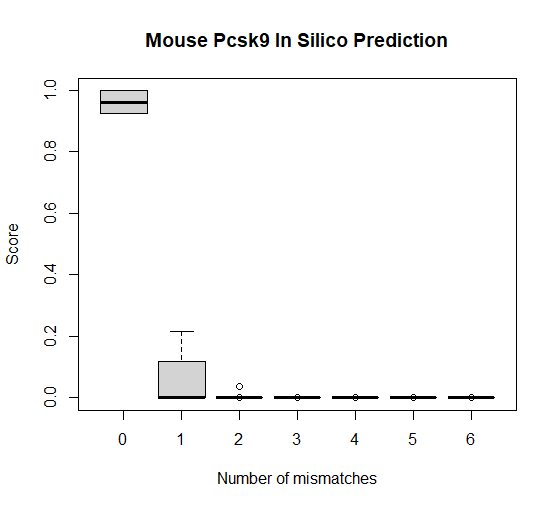 |
| *Albumin* | 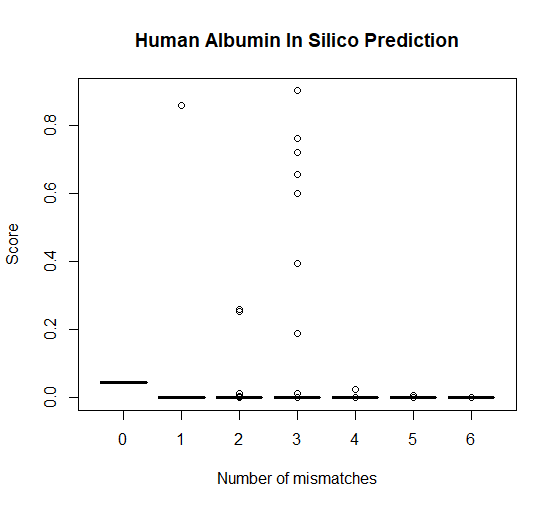 | 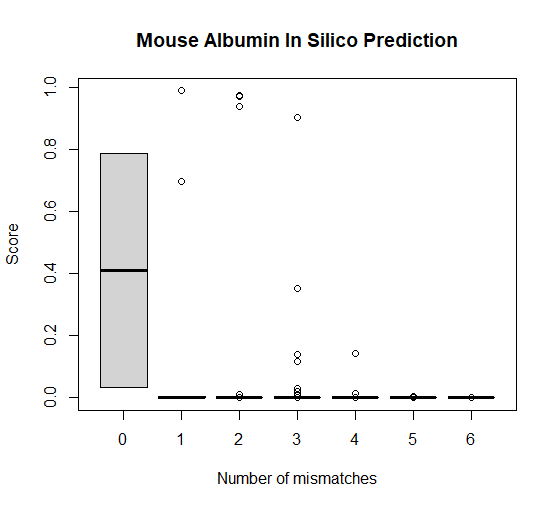 |

(d)

|  | Human | Mouse |
| --- | --- | --- |
| *PCSK9* | 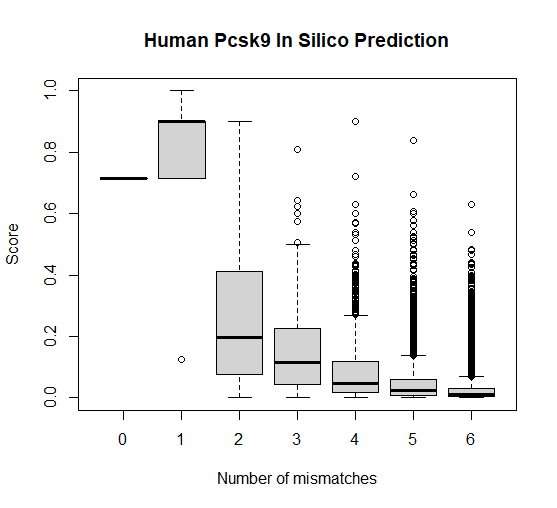 | 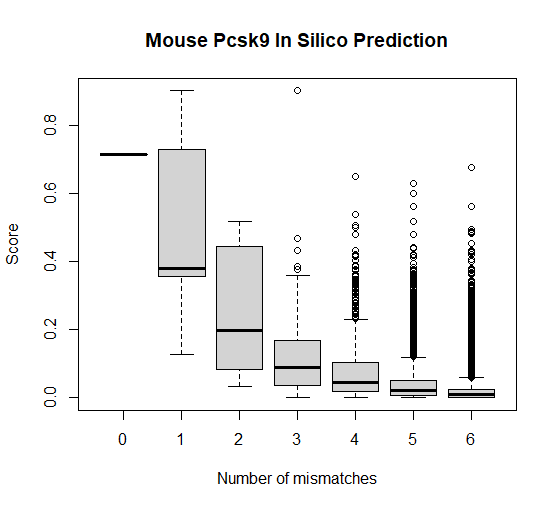 |
| *Albumin* | 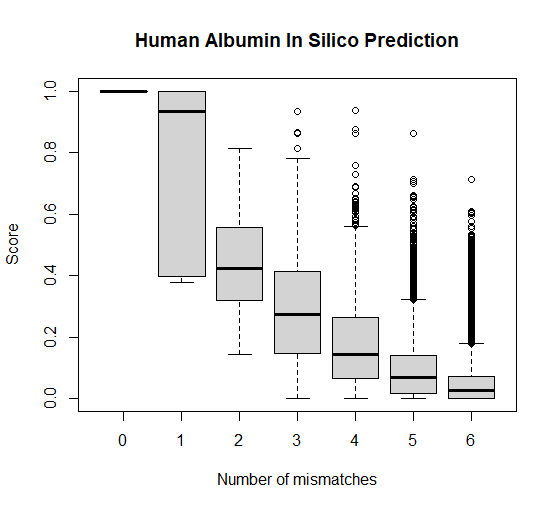 | 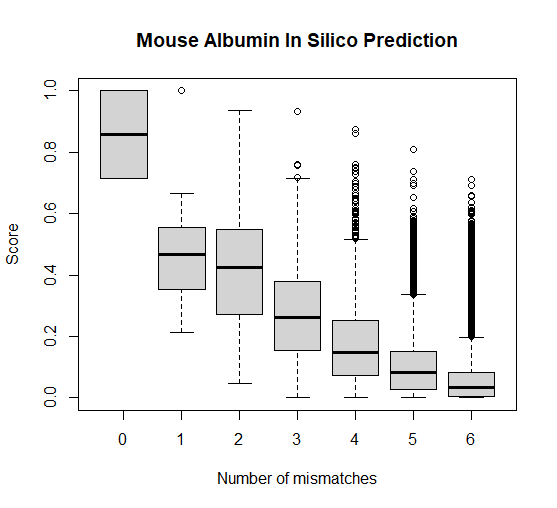 |

(e)

|  | Human | Mouse |
| --- | --- | --- |
| *PCSK9* | 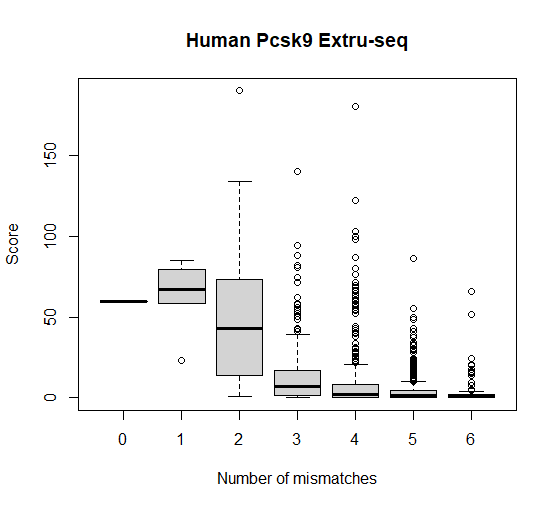 | 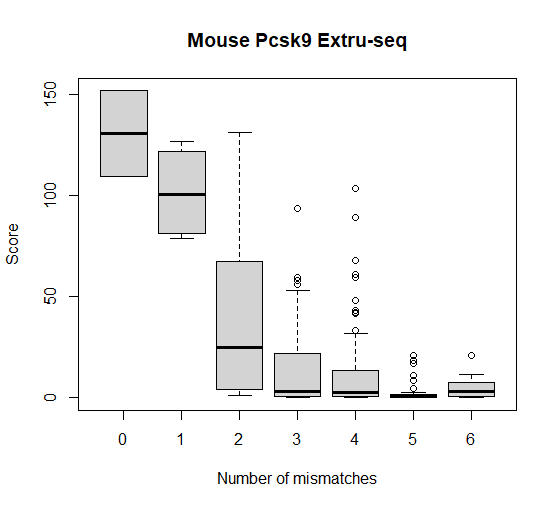 |
| *Albumin* | 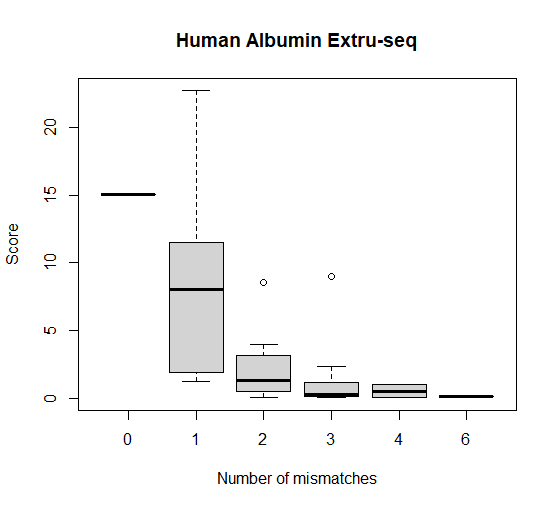 | 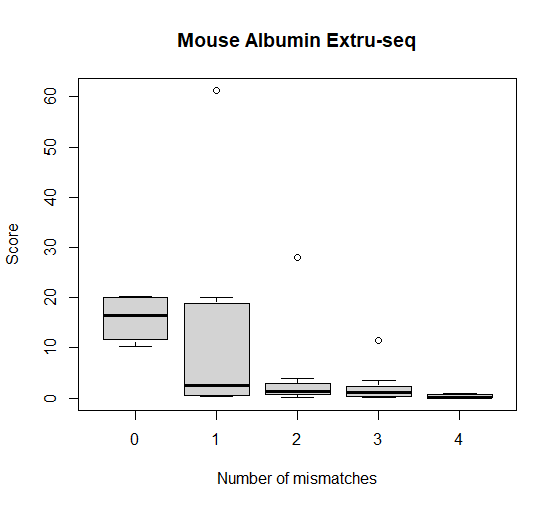 |

Fig. S8. Box and whisker plots showing results from different off-target prediction methods for promiscuous sgRNAs targeting *PSK9* and *Albumin*. Scores are plotted vs. the number of mismatches in the predicted off-target loci for (a) GUIDE-seq (score, sequence read counts), (b) Digenome-seq (DNA cleavage score), (c) *in silico* prediction (CROP score), (d) *in silico* prediction (CFD score), and (e) Extru-seq (DNA cleavage score). Human HEK293T cells and mouse NIH-3T3 cells were used in the experiments. Outliers in the box and whisker plots are represented by empty circles.

|  | i.v. injection | Subretinal injection |
| --- | --- | --- |
| *PCSK9* |  |  |
| *Albumin* |  |  |

Fig. S9. Indel ratios calculated following analysis of genomic DNA obtained from organs from C57BL/6 mice injected with two AAV8 vectors, respectively expressing Cas9 and sgRNA targeting either *PCSK9* or *Albumin*. Error bars indicate s.e.m. (n = 3). NR: Neural Retina, RPE: Retinal Pigment Epithelial cells.

(a)

(b)

(c)

(d)

Fig. S10. Validation results from targeted deep sequencing of the top 10 predicted off-target sites for sgRNAs targeting (a) human *PCSK9*, (b) human *Albumin*, (c) mouse *Pcsk9*, and (d) mouse *Albumin*. For validation, the frequency of indels at the off-target site must be higher than 0.1% and the equation (Indel frequency at the off-target locus)/(Indel frequency in the control with no Cas9 treatment) > 2 should be satisfied^9^. A red * indicates that the target was confirmed manually (Additional File 3: Table S2).

(a)


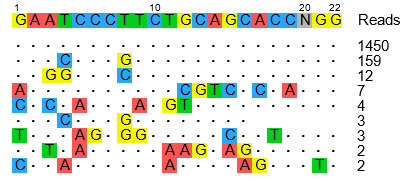


(b)


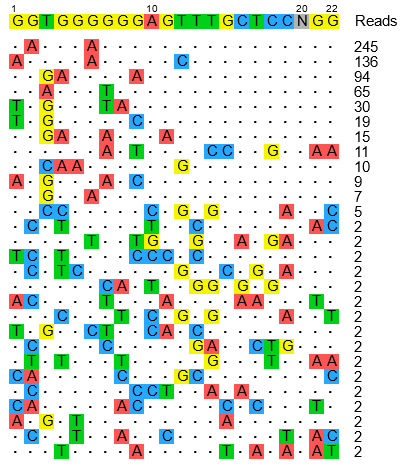


(c)


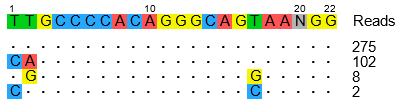


Fig. S11. GUIDE-seq results obtained from HeLa cells using sgRNAs targeting (a) *FANCF*, (b) *VEGFA*, or (c) *HBB.*

(a)


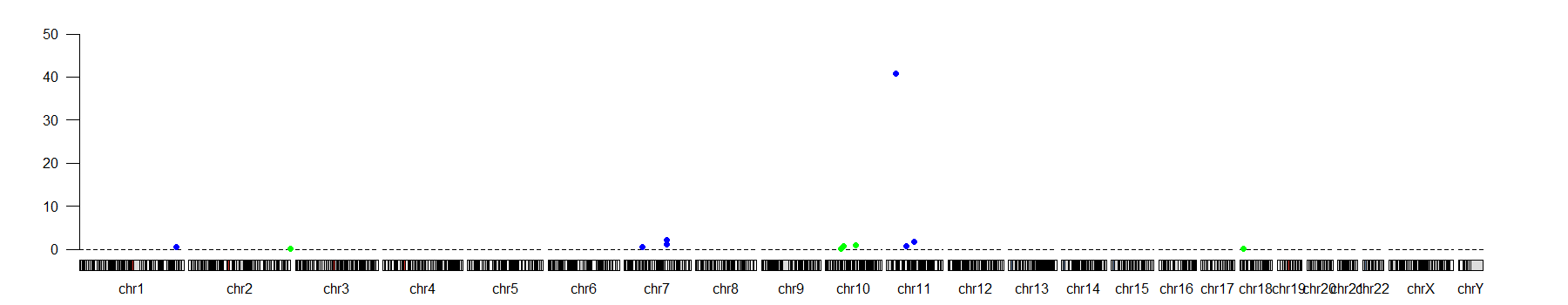


(b)


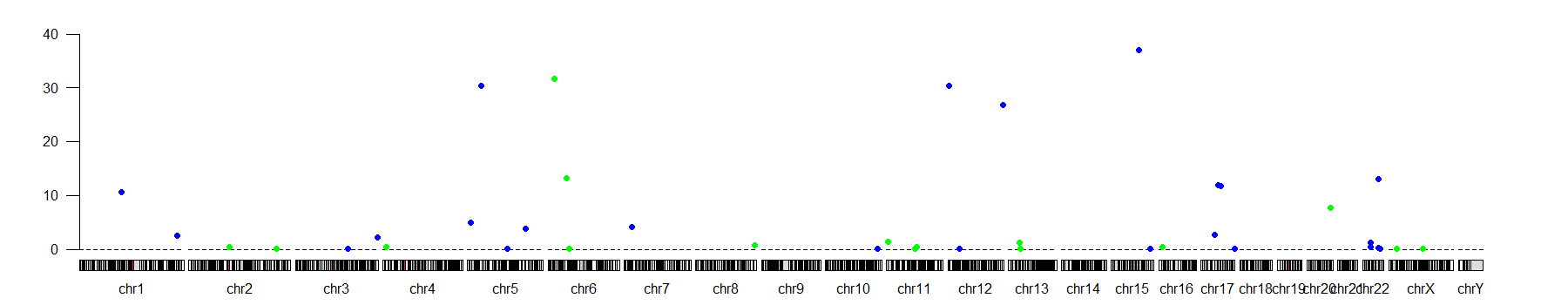


(c)


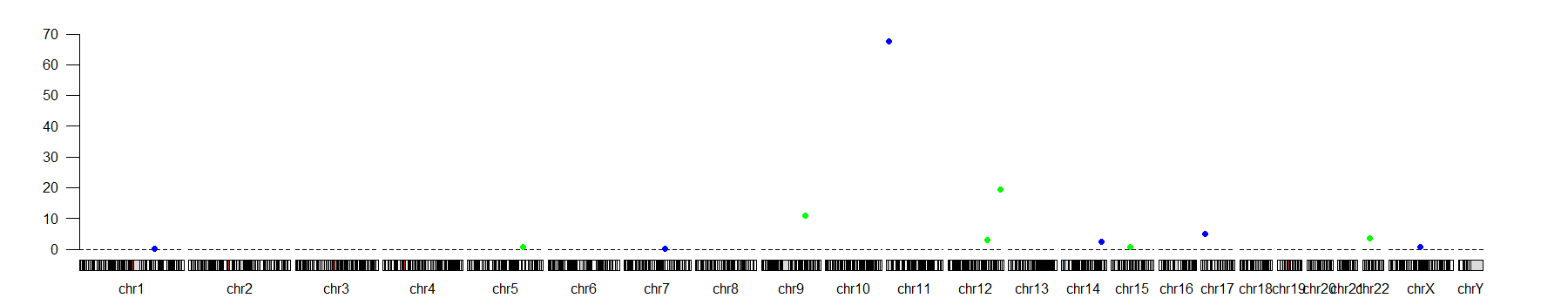


Fig. S12. Manhattan plot of Extru-seq results obtained from HeLa cells using sgRNAs targeting (a) *FANCF*, (b) *VEGFA* or (c) *HBB*. The y-axis represents the DNA cleavage score.

(a)

| Targeted gene | ***FANCF*** | DIG-seq | Digenome-seq | Extru-seq | GUIDE-seq |
| --- | --- | --- | --- | --- | --- |
| Number of candidate off-target sites found | | 36 | 46 | 12+4* | 4 |
| Validation | Validated | 8 | 8 | 5+4* | 3 |
|  | FALSE | 25 | 17 | 4 | 1 |
|  | Undetermined | 3 (repeat) | 21 | 3 (repeat) | 0 |
|  | Validation ratio | 24.24% | 32.00% | 55.56%  (69.23%*) | 75.00% |
|  | Validation ratio for the 10 sites with the highest scores | 40.00% | 60.00% | 55.56% | 75.00% |

(b)

| Gene | ***VEGFA*** | DIG-seq | Digenome-seq | Extru-seq | GUIDE-seq |
| --- | --- | --- | --- | --- | --- |
| Number of candidate off-target sites found | | 31 | 80 | 40+4* | 18 |
| Validation | Validated | 17 | 23 | 19+4* | 11 |
|  | FALSE | 14 | 24 | 20 | 1 |
|  | Undetermined | 0 | 33 | 1 | 6 |
|  | Validation ratio | 54.84% | 48.94% | 48.72%  (53.49%*) | 91.67% |
|  | Validation ratio for the 10 sites with the highest scores | 70.00% | 60.00% | 100.00% | 90.00% |

(c)

| Targeted gene | ***HBB*** | DIG-seq | Digenome-seq (HAP1) | Extru-seq | GUIDE-seq |
| --- | --- | --- | --- | --- | --- |
| Number of candidate off-target sites found | | 44 | 20 | 12 | 4 |
| Validation | Validated | 4 | 4 | 4 | 4 |
|  | FALSE | 40 | 13 | 8 | 0 |
|  | Undetermined | 0 | 3 | 0 | 0 |
|  | Validation ratio | 9.09% | 23.53% | 33.33% | 100.00% |
|  | Validation ratio for the 10 sites with the highest scores | 30.00% | 40% | 40.00% | 100.00% |

Fig. S13. Validation results from targeted deep sequencing of the top 10 predicted off-target sites for sgRNAs targeting (a) human *FANCF*, (b) human *VEGFA*, and (c) human *HBB*. For validation, the frequency of indels at the off-target site needs to be higher than 0.1% and the expression (Indel frequency at the off-target locus)/(Indel frequency in the control) > 2 should be satisfied^9^. A red * indicates that the target was confirmed manually (Additional File 1: Table S2).

(a)

|  | h*PCSK9* | h*Albumin* | m*PCSK9* | m*Albumin* |
| --- | --- | --- | --- | --- |
| GUIDE-seq : Digenome-seq | 137 | 43 | 70 | 12 |
| GUIDE-seq : *in silico* | 119 | 55 | 126 | 25 |
| GUIDE-seq : Extru-seq | 128 | 28 | 93 | 22 |
| Digenome-seq : *in silico* | 3311 | 222 | 128 | 54 |
| Digenome-seq : Extru-seq | 1288 | 30 | 110 | 17 |
| *in silico* : Extru-seq | 1133 | 35 | 189 | 54 |

(b)

|  | h*HBB* | h*VEGFA* | h*FANCF* |
| --- | --- | --- | --- |
| DIG-seq : Digenome-seq | 17 | 29 | 24 |
| DIG-seq : Extru-seq | 12 | 28 | 11 |
| DIG-seq : GUIDE-seq | 4 | 10 | 3 |
| Digenome-seq : Extru-seq | 10 | 38 | 10 |
| Digenome-seq : GUIDE-seq | 4 | 11 | 3 |
| GUIDE-seq : Extru-seq | 4 | 10 | 2 |

Fig. S14. Number of samples found in the intersections of Venn diagrams showing the overlap between off-target sites predicted by different methods for sgRNAs targeting (a) human *PCSK9*, human *Albuimin*, mouse *PCSK9*, and mouse *Albumin* and (b) human *FANCF*, human *VEGFA*, and human *HBB*. Cases in which n ≥ 16 (the minimum number of samples required for asymptotic nonparametric Wilcoxon rank tests) are colored red.

(a)

(b)

(c)

(d)

(e)

(f)

(g)

Fig. S15. Off-target sites, predicted by Extru-seq or GUIDE-seq and validated by deep sequencing, for sgRNAs targeting (a) human *PCSK9*, (b) human *Albuimin*, (c) mouse *PCSK9*, (d) mouse *Albumin*, (e) human *FANCF*, (f) human *VEGFA*, and (g) human *HBB*. For validation, the frequency of indels at the off-target site must be higher than 0.1% and the expression (Indel frequency at the off-target locus)/(Indel frequency in the control) > 2 should be satisfied^9^. + means that the target was predicted by the indicated method, whereas a blank box means that the target was missed by the indicated method. The miss rate was calculated as the ratio of the number of blank boxes to the total number of boxes in the column. A red * indicates that the target was confirmed manually (Additional File 3: Table S2).

(a)


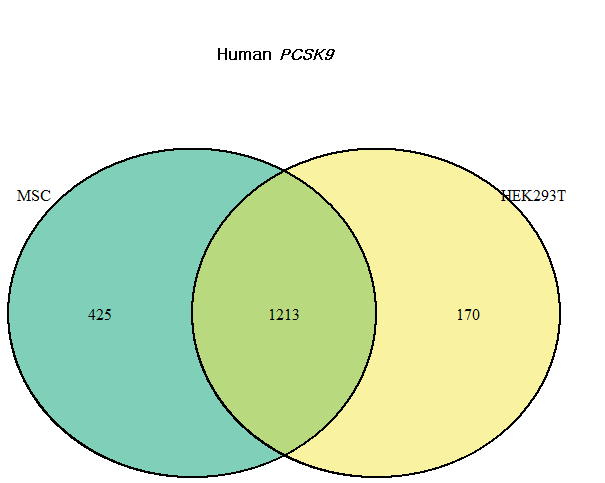


(b)


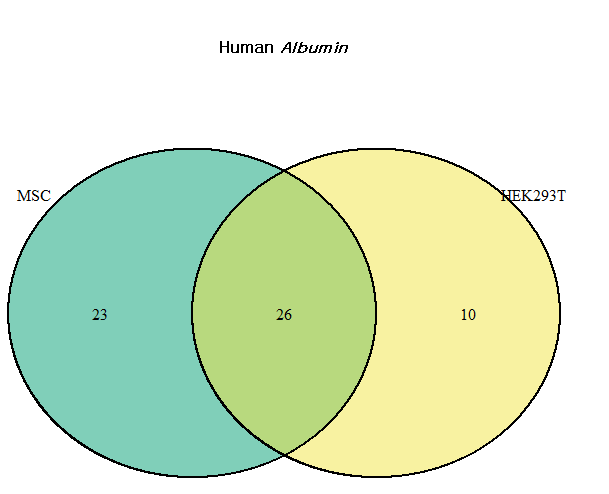


Fig. S16. Venn diagrams showing the number of predicted off-target sites for sgRNAs targeting (a) human *PCSK9* and (b) human *Albumin* in MSCs and HEK293T cells, determined by Extru-seq.

(a)

(b)

Fig. S17. The top 10 potential off-target loci predicted by Extru-seq for MSCs and HEK293T cells, as well as the rankings of the loci by other prediction methods, for sgRNAs targeting (a) human *PCSK9* and (b) human *Albumin*.

Fig. S18. p-values obtained by the normalized rank sum test for each pair of off-target prediction methods for promiscuous sgRNAs targeting *PCSK9* and *Albumin* in MSCs and HEK293T cells.
