# Supplementary material for: Extru-seq: a method for predicting genome-wide Cas9 off-target sites with advantages of both cell-based and in vitro approaches
Source: Genome Biol. 2023 Jan 10;24:4. doi: 10.1186/s13059-022-02842-4 (PMC9832775; doi:10.1186/s13059-022-02842-4)
Supplement: Supplementary file 3 — Additional file 3: Table S2. Manually validated off-target sites from Extru-seq WGS data visualized using IGV. [file 13059_2022_2842_MOESM3_ESM.pptx]

## Slide 1
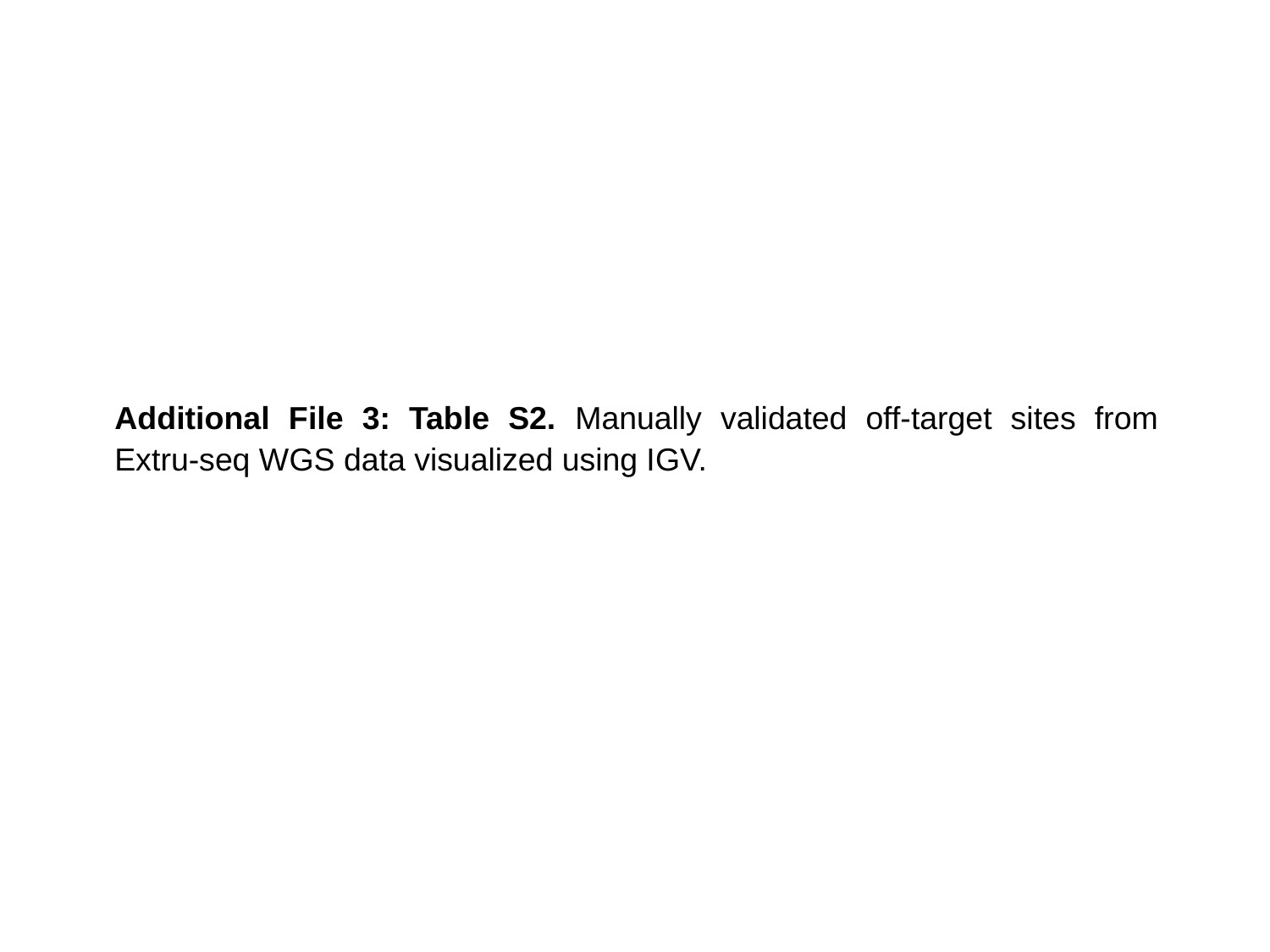

Additional File 3: Table S2. Manually validated off-target sites from Extru-seq WGS data visualized using IGV.

## Slide 2
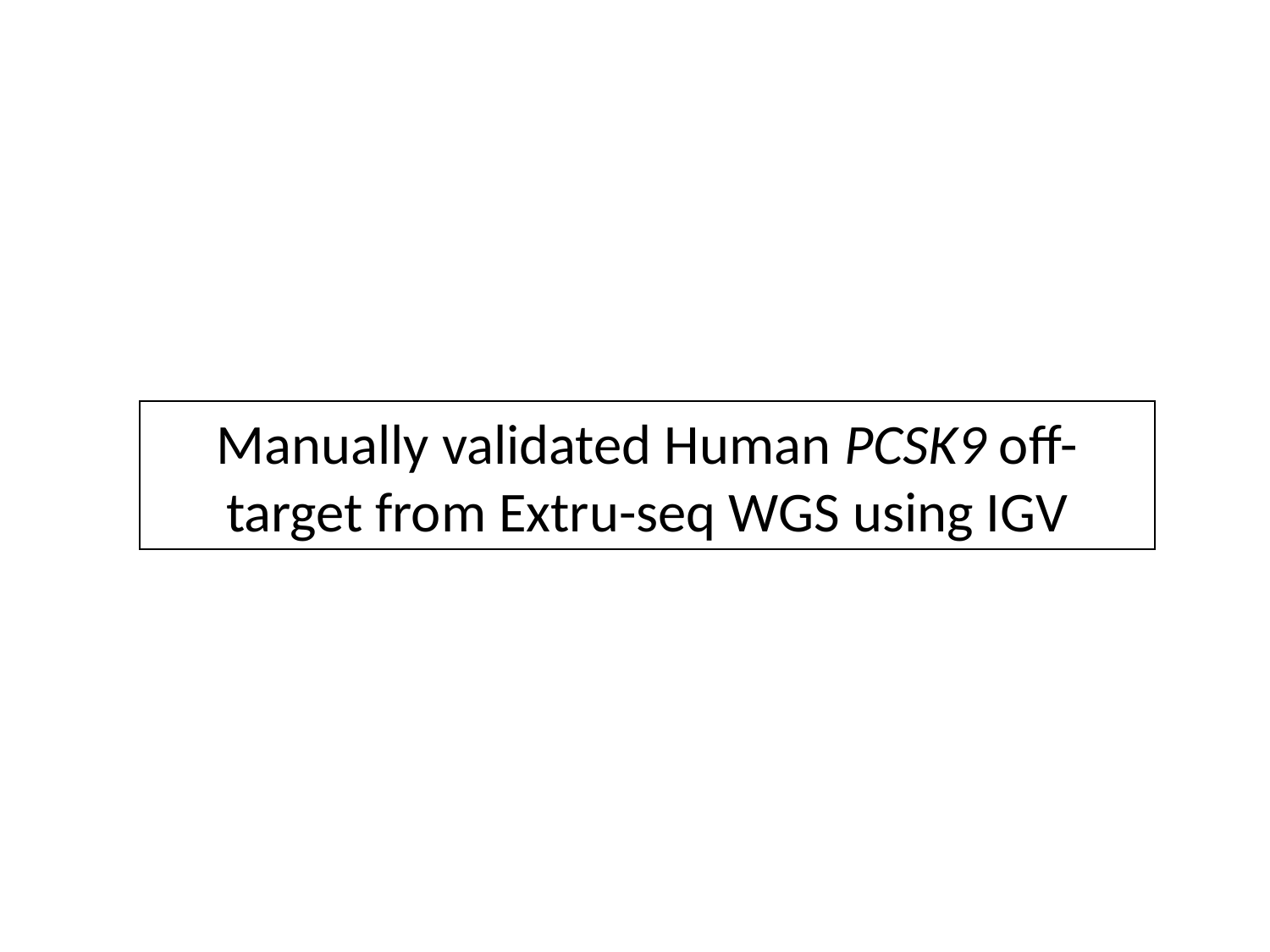

Manually validated Human PCSK9 off-target from Extru-seq WGS using IGV

## Slide 3
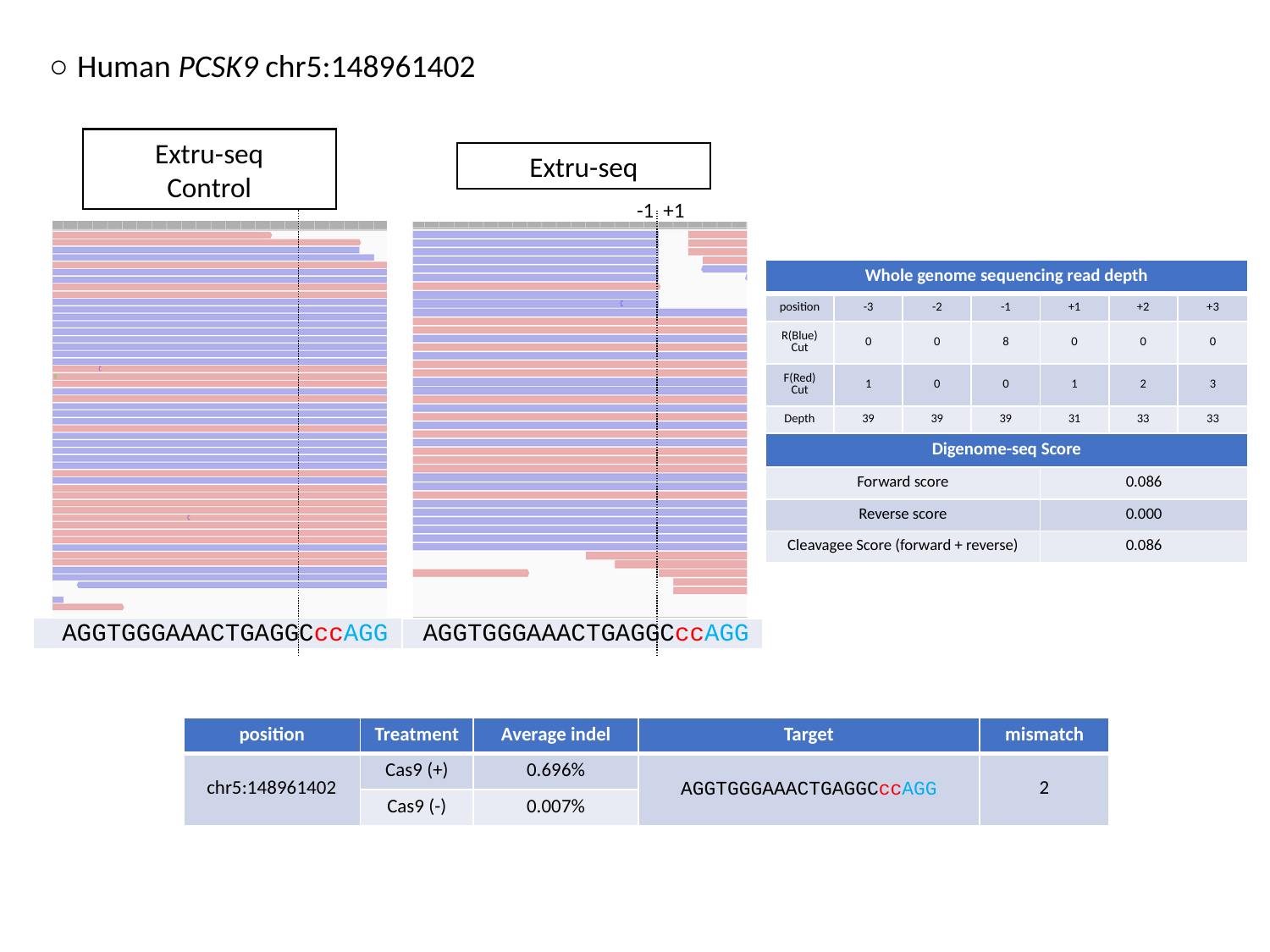

○ Human PCSK9 chr5:148961402
Extru-seq
Control
Extru-seq
-1
+1
| Whole genome sequencing read depth | | | | | | |
| --- | --- | --- | --- | --- | --- | --- |
| position | -3 | -2 | -1 | +1 | +2 | +3 |
| R(Blue) Cut | 0 | 0 | 8 | 0 | 0 | 0 |
| F(Red) Cut | 1 | 0 | 0 | 1 | 2 | 3 |
| Depth | 39 | 39 | 39 | 31 | 33 | 33 |
| Digenome-seq Score | | | | | | |
| Forward score | | | | 0.086 | | |
| Reverse score | | | | 0.000 | | |
| Cleavagee Score (forward + reverse) | | | | 0.086 | | |
| AGGTGGGAAACTGAGGCccAGG |
| --- |
| AGGTGGGAAACTGAGGCccAGG |
| --- |
| position | Treatment | Average indel | Target | mismatch |
| --- | --- | --- | --- | --- |
| chr5:148961402 | Cas9 (+) | 0.696% | AGGTGGGAAACTGAGGCccAGG | 2 |
| | Cas9 (-) | 0.007% | | |

## Slide 4
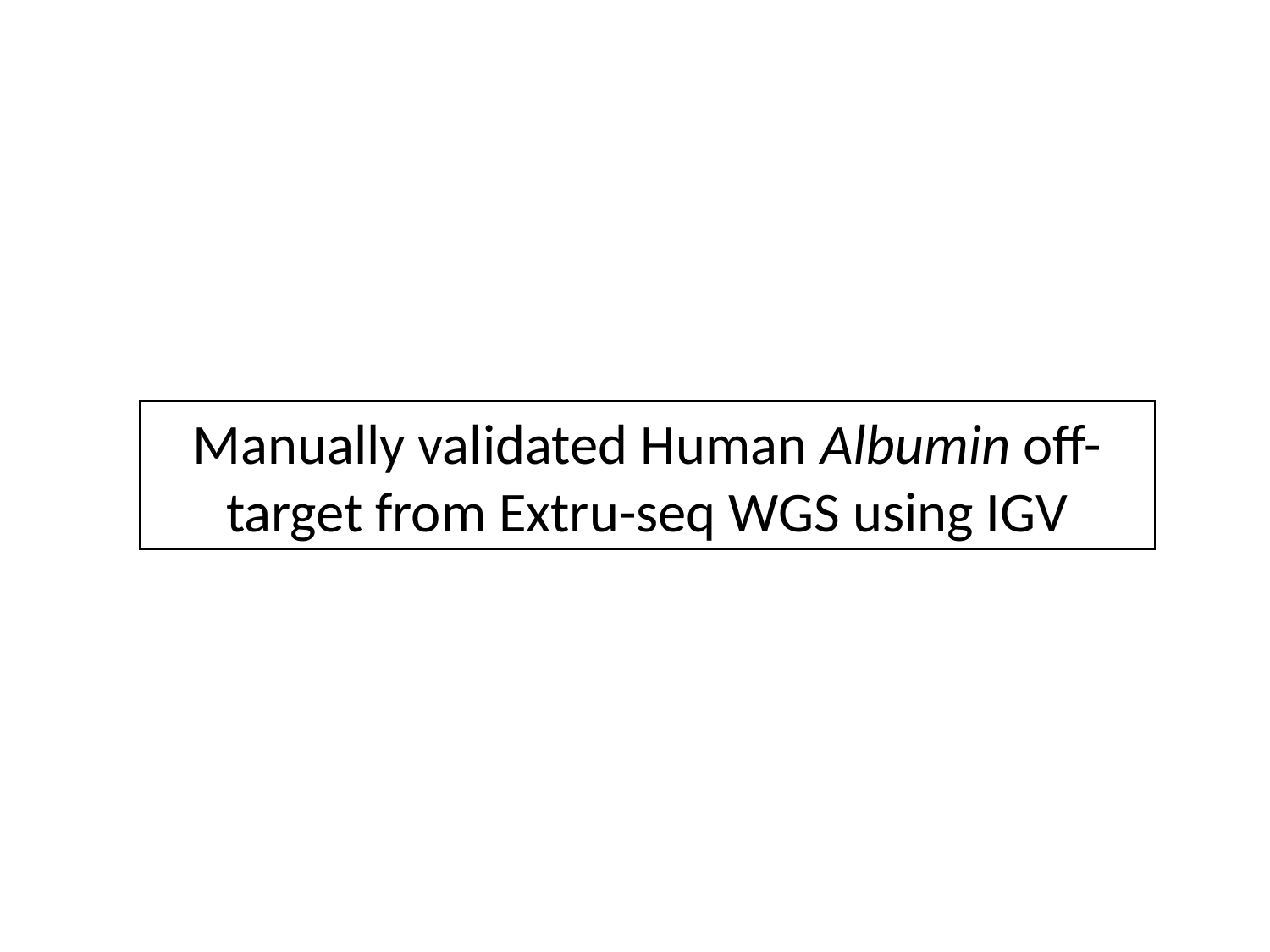

Manually validated Human Albumin off-target from Extru-seq WGS using IGV

## Slide 5
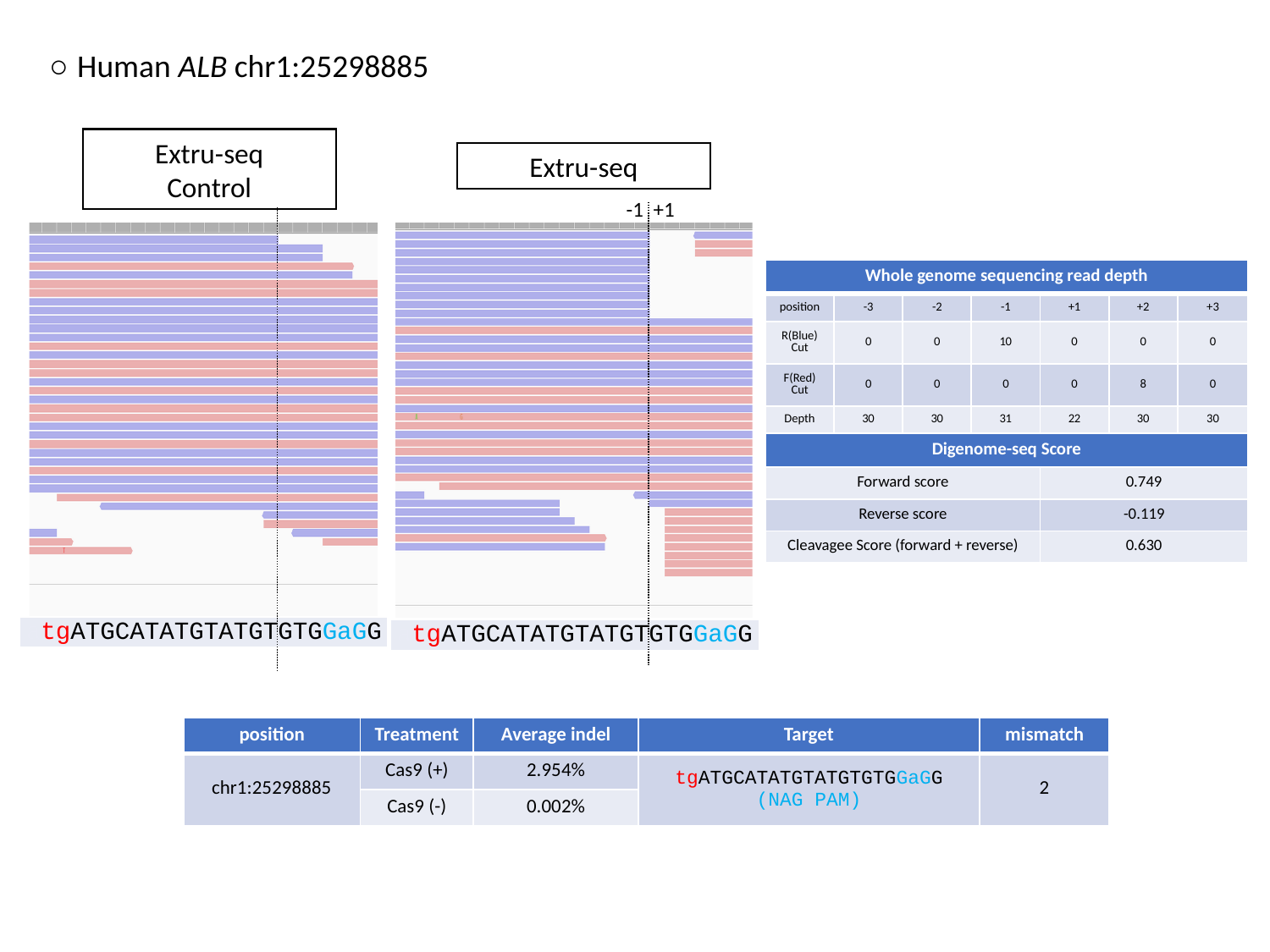

○ Human ALB chr1:25298885
Extru-seq
Control
Extru-seq
-1
+1
| Whole genome sequencing read depth | | | | | | |
| --- | --- | --- | --- | --- | --- | --- |
| position | -3 | -2 | -1 | +1 | +2 | +3 |
| R(Blue) Cut | 0 | 0 | 10 | 0 | 0 | 0 |
| F(Red) Cut | 0 | 0 | 0 | 0 | 8 | 0 |
| Depth | 30 | 30 | 31 | 22 | 30 | 30 |
| Digenome-seq Score | | | | | | |
| Forward score | | | | 0.749 | | |
| Reverse score | | | | -0.119 | | |
| Cleavagee Score (forward + reverse) | | | | 0.630 | | |
| atgATGCATATGTATGTGTGGaGG |
| --- |
| atgATGCATATGTATGTGTGGaGG |
| --- |
| position | Treatment | Average indel | Target | mismatch |
| --- | --- | --- | --- | --- |
| chr1:25298885 | Cas9 (+) | 2.954% | tgATGCATATGTATGTGTGGaGG (NAG PAM) | 2 |
| | Cas9 (-) | 0.002% | | |

## Slide 6
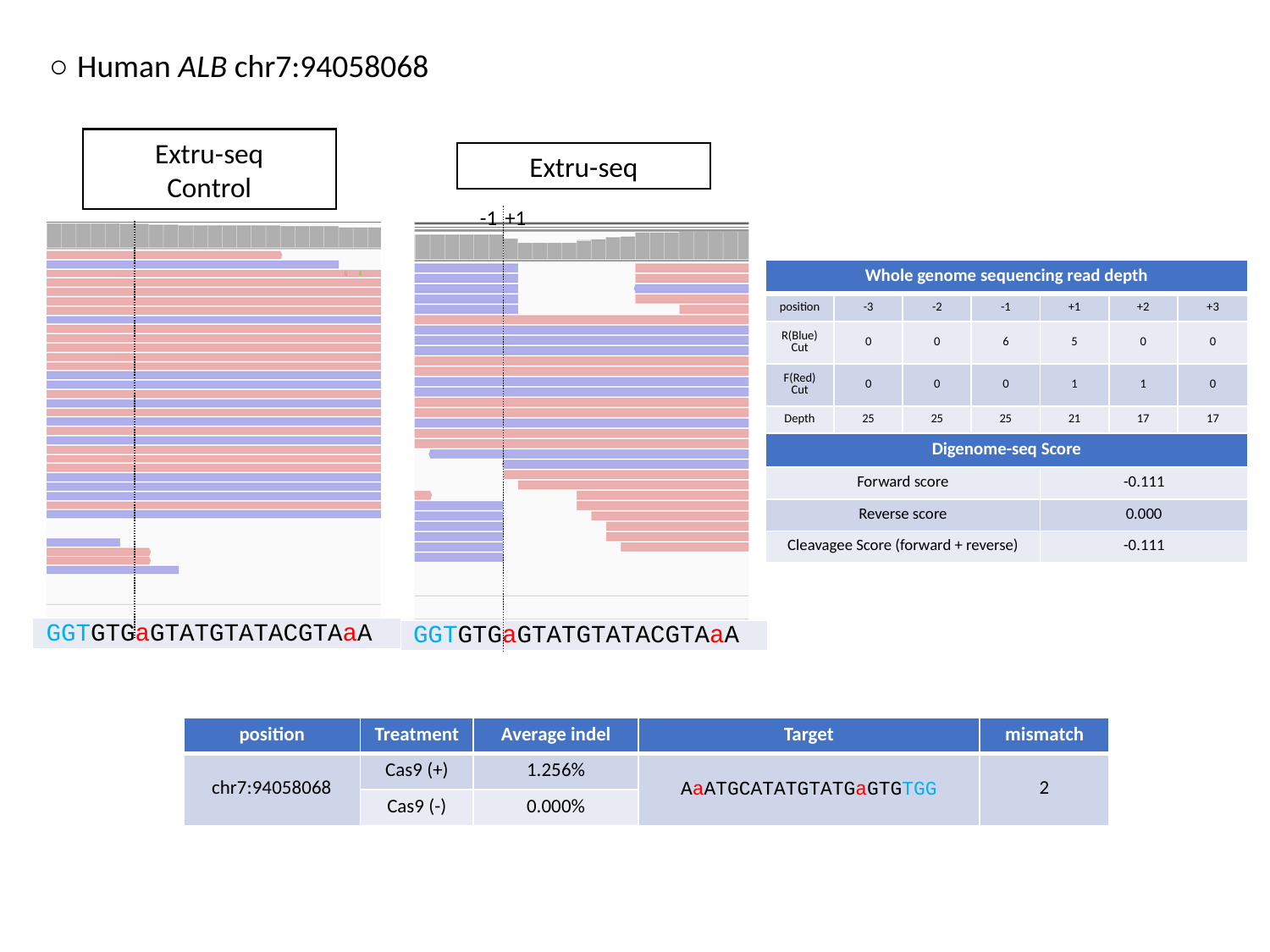

○ Human ALB chr7:94058068
Extru-seq
Control
Extru-seq
+1
-1
| Whole genome sequencing read depth | | | | | | |
| --- | --- | --- | --- | --- | --- | --- |
| position | -3 | -2 | -1 | +1 | +2 | +3 |
| R(Blue) Cut | 0 | 0 | 6 | 5 | 0 | 0 |
| F(Red) Cut | 0 | 0 | 0 | 1 | 1 | 0 |
| Depth | 25 | 25 | 25 | 21 | 17 | 17 |
| Digenome-seq Score | | | | | | |
| Forward score | | | | -0.111 | | |
| Reverse score | | | | 0.000 | | |
| Cleavagee Score (forward + reverse) | | | | -0.111 | | |
| GGTGTGaGTATGTATACGTAaAa |
| --- |
| GGTGTGaGTATGTATACGTAaAa |
| --- |
| position | Treatment | Average indel | Target | mismatch |
| --- | --- | --- | --- | --- |
| chr7:94058068 | Cas9 (+) | 1.256% | AaATGCATATGTATGaGTGTGG | 2 |
| | Cas9 (-) | 0.000% | | |

## Slide 7
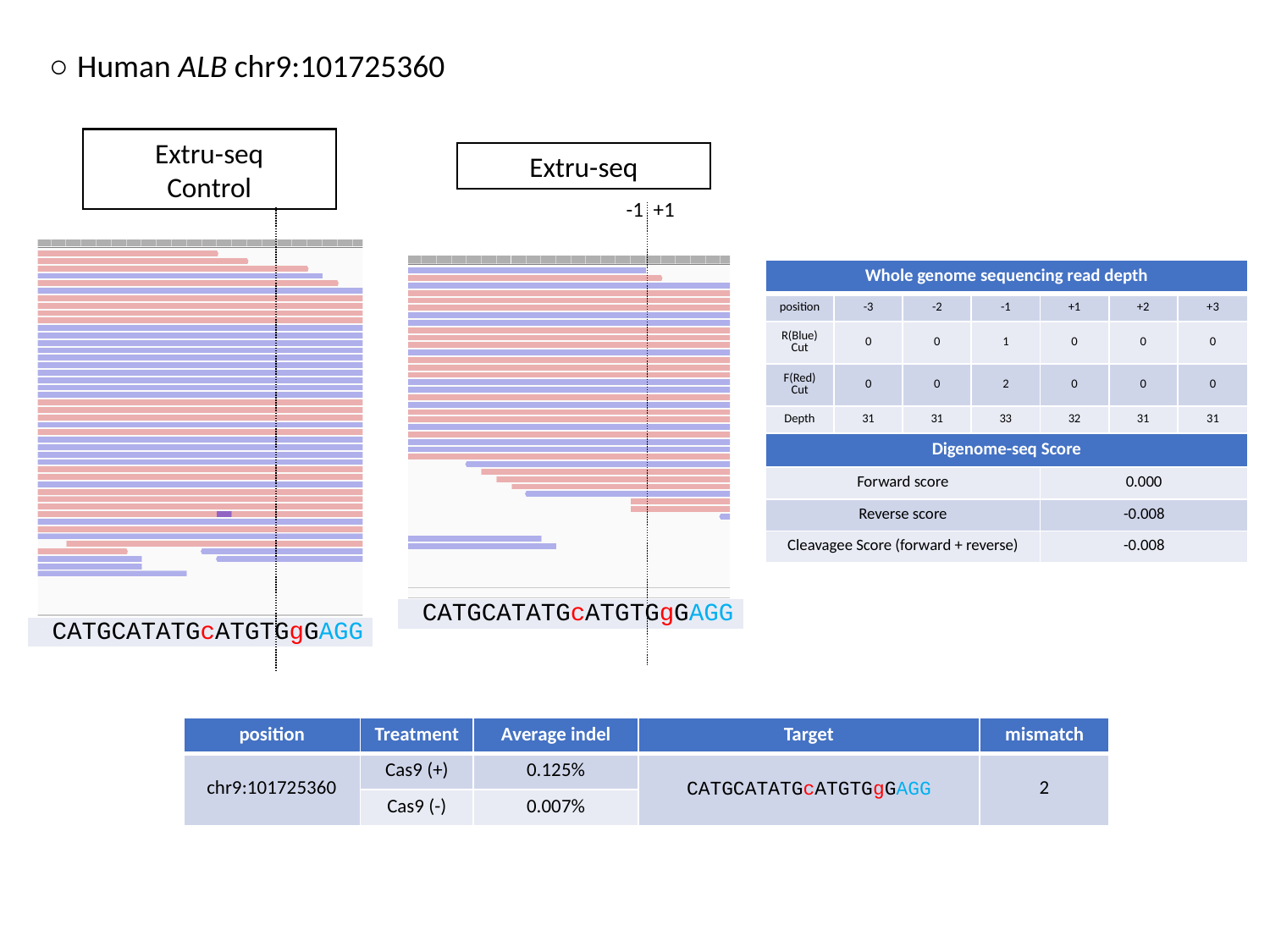

○ Human ALB chr9:101725360
Extru-seq
Control
Extru-seq
-1
+1
| Whole genome sequencing read depth | | | | | | |
| --- | --- | --- | --- | --- | --- | --- |
| position | -3 | -2 | -1 | +1 | +2 | +3 |
| R(Blue) Cut | 0 | 0 | 1 | 0 | 0 | 0 |
| F(Red) Cut | 0 | 0 | 2 | 0 | 0 | 0 |
| Depth | 31 | 31 | 33 | 32 | 31 | 31 |
| Digenome-seq Score | | | | | | |
| Forward score | | | | 0.000 | | |
| Reverse score | | | | -0.008 | | |
| Cleavagee Score (forward + reverse) | | | | -0.008 | | |
| tCATGCATATGcATGTGgGAGG |
| --- |
| tCATGCATATGcATGTGgGAGG |
| --- |
| position | Treatment | Average indel | Target | mismatch |
| --- | --- | --- | --- | --- |
| chr9:101725360 | Cas9 (+) | 0.125% | CATGCATATGcATGTGgGAGG | 2 |
| | Cas9 (-) | 0.007% | | |

## Slide 8
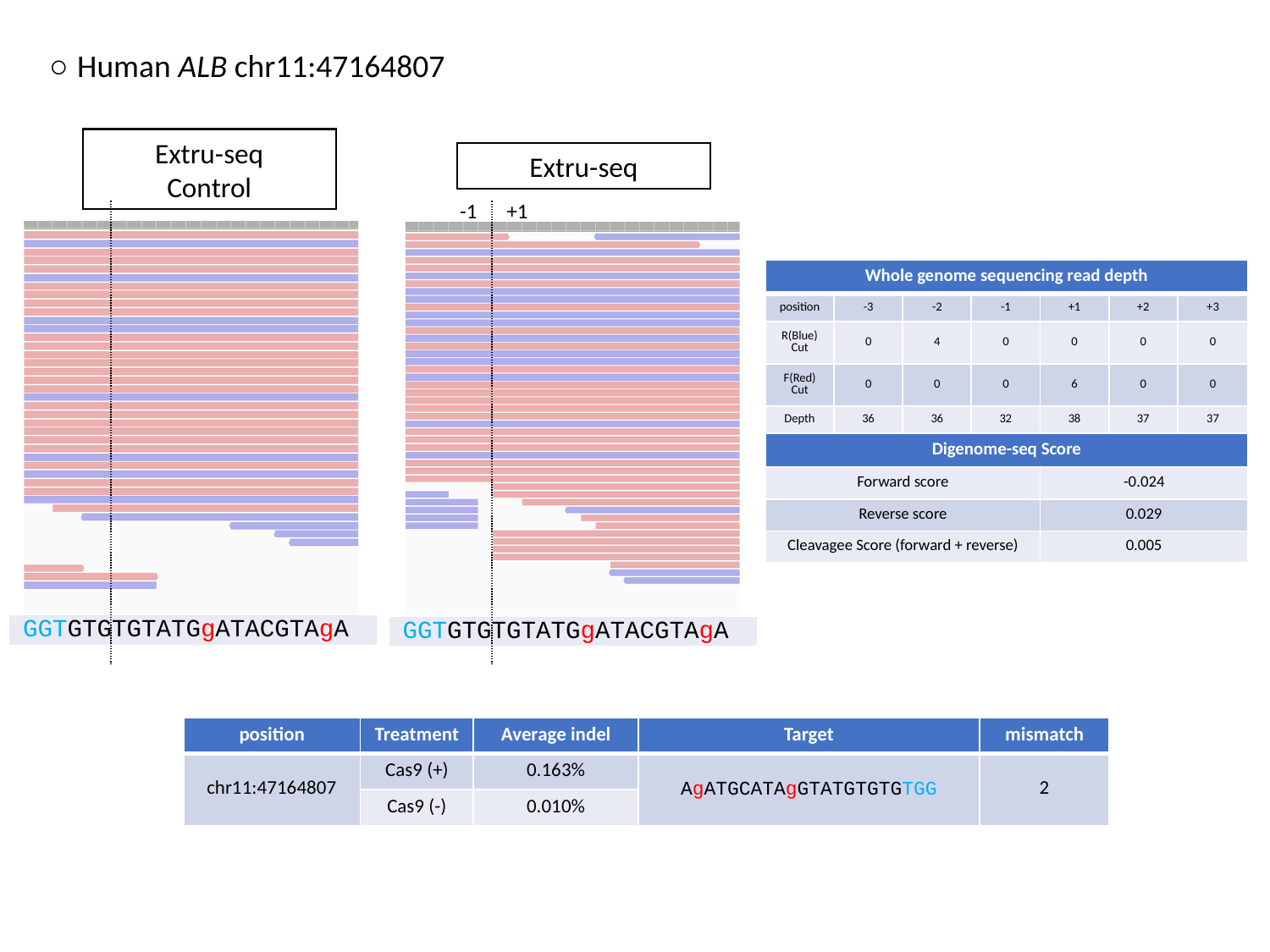

○ Human ALB chr11:47164807
Extru-seq
Control
Extru-seq
-1
+1
| Whole genome sequencing read depth | | | | | | |
| --- | --- | --- | --- | --- | --- | --- |
| position | -3 | -2 | -1 | +1 | +2 | +3 |
| R(Blue) Cut | 0 | 4 | 0 | 0 | 0 | 0 |
| F(Red) Cut | 0 | 0 | 0 | 6 | 0 | 0 |
| Depth | 36 | 36 | 32 | 38 | 37 | 37 |
| Digenome-seq Score | | | | | | |
| Forward score | | | | -0.024 | | |
| Reverse score | | | | 0.029 | | |
| Cleavagee Score (forward + reverse) | | | | 0.005 | | |
| GGTGTGTGTATGgATACGTAgAc |
| --- |
| GGTGTGTGTATGgATACGTAgAc |
| --- |
| position | Treatment | Average indel | Target | mismatch |
| --- | --- | --- | --- | --- |
| chr11:47164807 | Cas9 (+) | 0.163% | AgATGCATAgGTATGTGTGTGG | 2 |
| | Cas9 (-) | 0.010% | | |

## Slide 9
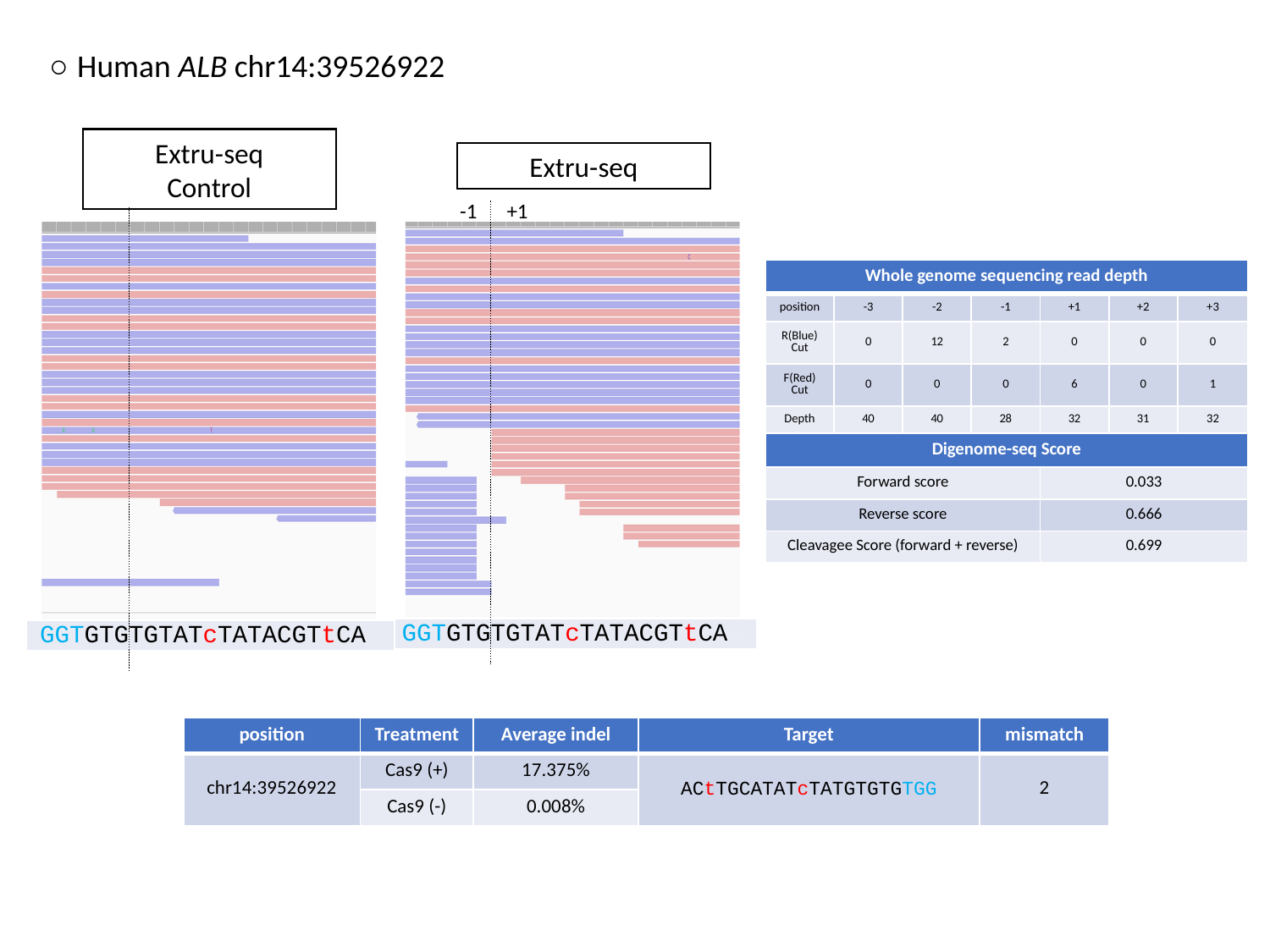

○ Human ALB chr14:39526922
Extru-seq
Control
Extru-seq
-1
+1
| Whole genome sequencing read depth | | | | | | |
| --- | --- | --- | --- | --- | --- | --- |
| position | -3 | -2 | -1 | +1 | +2 | +3 |
| R(Blue) Cut | 0 | 12 | 2 | 0 | 0 | 0 |
| F(Red) Cut | 0 | 0 | 0 | 6 | 0 | 1 |
| Depth | 40 | 40 | 28 | 32 | 31 | 32 |
| Digenome-seq Score | | | | | | |
| Forward score | | | | 0.033 | | |
| Reverse score | | | | 0.666 | | |
| Cleavagee Score (forward + reverse) | | | | 0.699 | | |
| GGTGTGTGTATcTATACGTtCAg |
| --- |
| GGTGTGTGTATcTATACGTtCAg |
| --- |
| position | Treatment | Average indel | Target | mismatch |
| --- | --- | --- | --- | --- |
| chr14:39526922 | Cas9 (+) | 17.375% | ACtTGCATATcTATGTGTGTGG | 2 |
| | Cas9 (-) | 0.008% | | |

## Slide 10
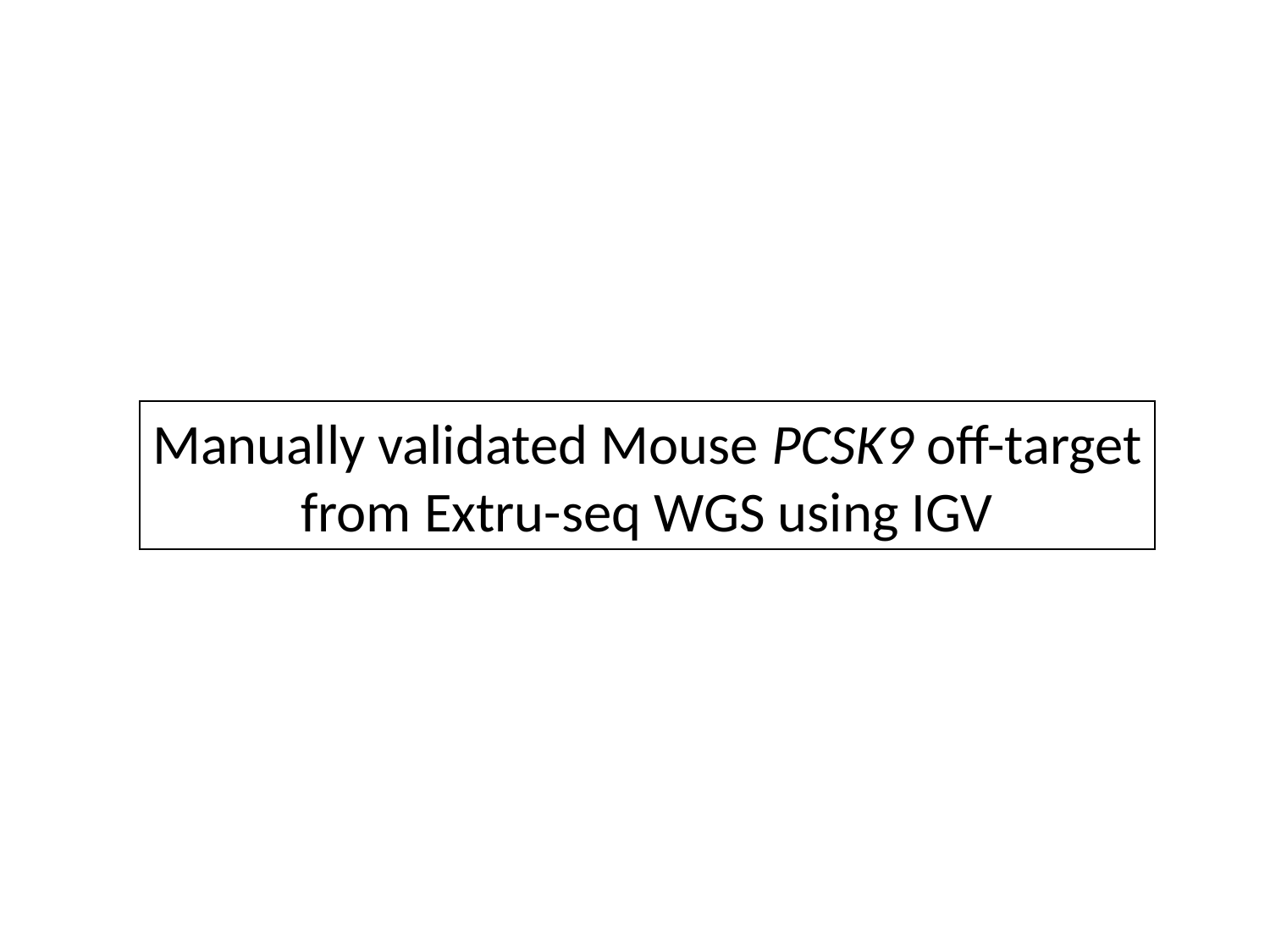

Manually validated Mouse PCSK9 off-target from Extru-seq WGS using IGV

## Slide 11
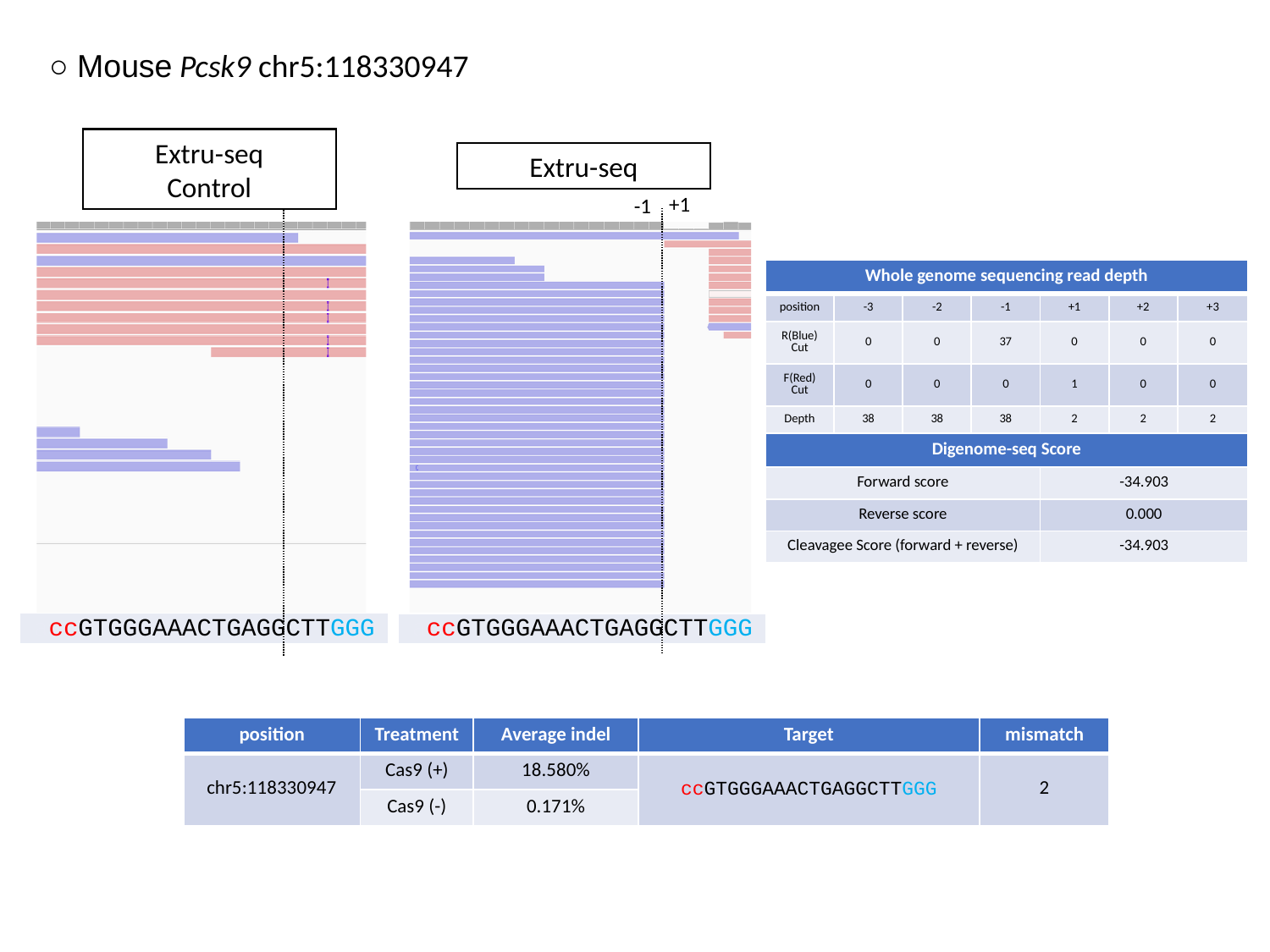

○ Mouse Pcsk9 chr5:118330947
Extru-seq
Control
Extru-seq
+1
-1
| Whole genome sequencing read depth | | | | | | |
| --- | --- | --- | --- | --- | --- | --- |
| position | -3 | -2 | -1 | +1 | +2 | +3 |
| R(Blue) Cut | 0 | 0 | 37 | 0 | 0 | 0 |
| F(Red) Cut | 0 | 0 | 0 | 1 | 0 | 0 |
| Depth | 38 | 38 | 38 | 2 | 2 | 2 |
| Digenome-seq Score | | | | | | |
| Forward score | | | | -34.903 | | |
| Reverse score | | | | 0.000 | | |
| Cleavagee Score (forward + reverse) | | | | -34.903 | | |
| accGTGGGAAACTGAGGCTTGGG |
| --- |
| accGTGGGAAACTGAGGCTTGGG |
| --- |
| position | Treatment | Average indel | Target | mismatch |
| --- | --- | --- | --- | --- |
| chr5:118330947 | Cas9 (+) | 18.580% | ccGTGGGAAACTGAGGCTTGGG | 2 |
| | Cas9 (-) | 0.171% | | |

## Slide 12
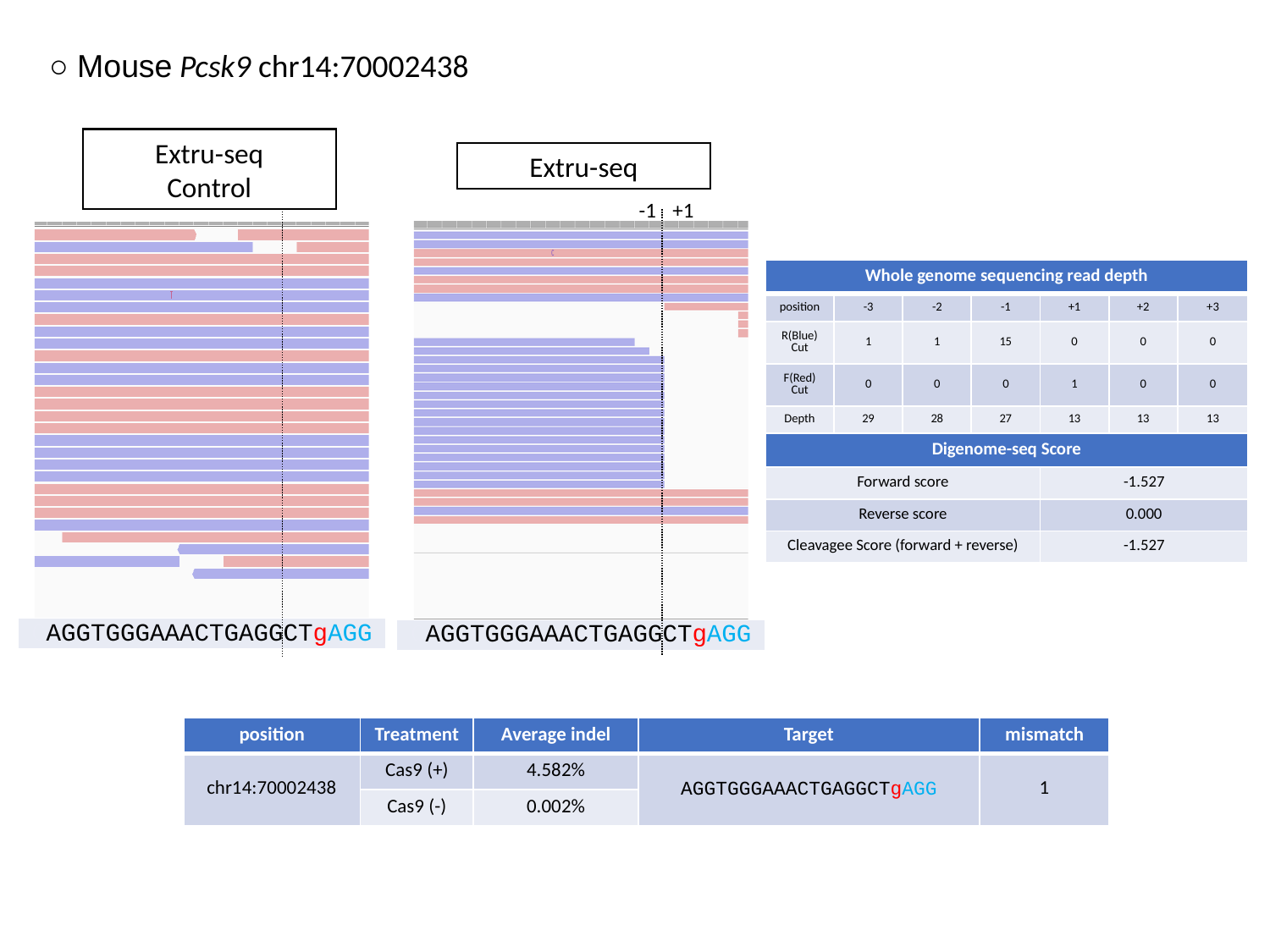

○ Mouse Pcsk9 chr14:70002438
Extru-seq
Control
Extru-seq
-1
+1
| Whole genome sequencing read depth | | | | | | |
| --- | --- | --- | --- | --- | --- | --- |
| position | -3 | -2 | -1 | +1 | +2 | +3 |
| R(Blue) Cut | 1 | 1 | 15 | 0 | 0 | 0 |
| F(Red) Cut | 0 | 0 | 0 | 1 | 0 | 0 |
| Depth | 29 | 28 | 27 | 13 | 13 | 13 |
| Digenome-seq Score | | | | | | |
| Forward score | | | | -1.527 | | |
| Reverse score | | | | 0.000 | | |
| Cleavagee Score (forward + reverse) | | | | -1.527 | | |
| CAGGTGGGAAACTGAGGCTgAGG |
| --- |
| CAGGTGGGAAACTGAGGCTgAGG |
| --- |
| position | Treatment | Average indel | Target | mismatch |
| --- | --- | --- | --- | --- |
| chr14:70002438 | Cas9 (+) | 4.582% | AGGTGGGAAACTGAGGCTgAGG | 1 |
| | Cas9 (-) | 0.002% | | |

## Slide 13
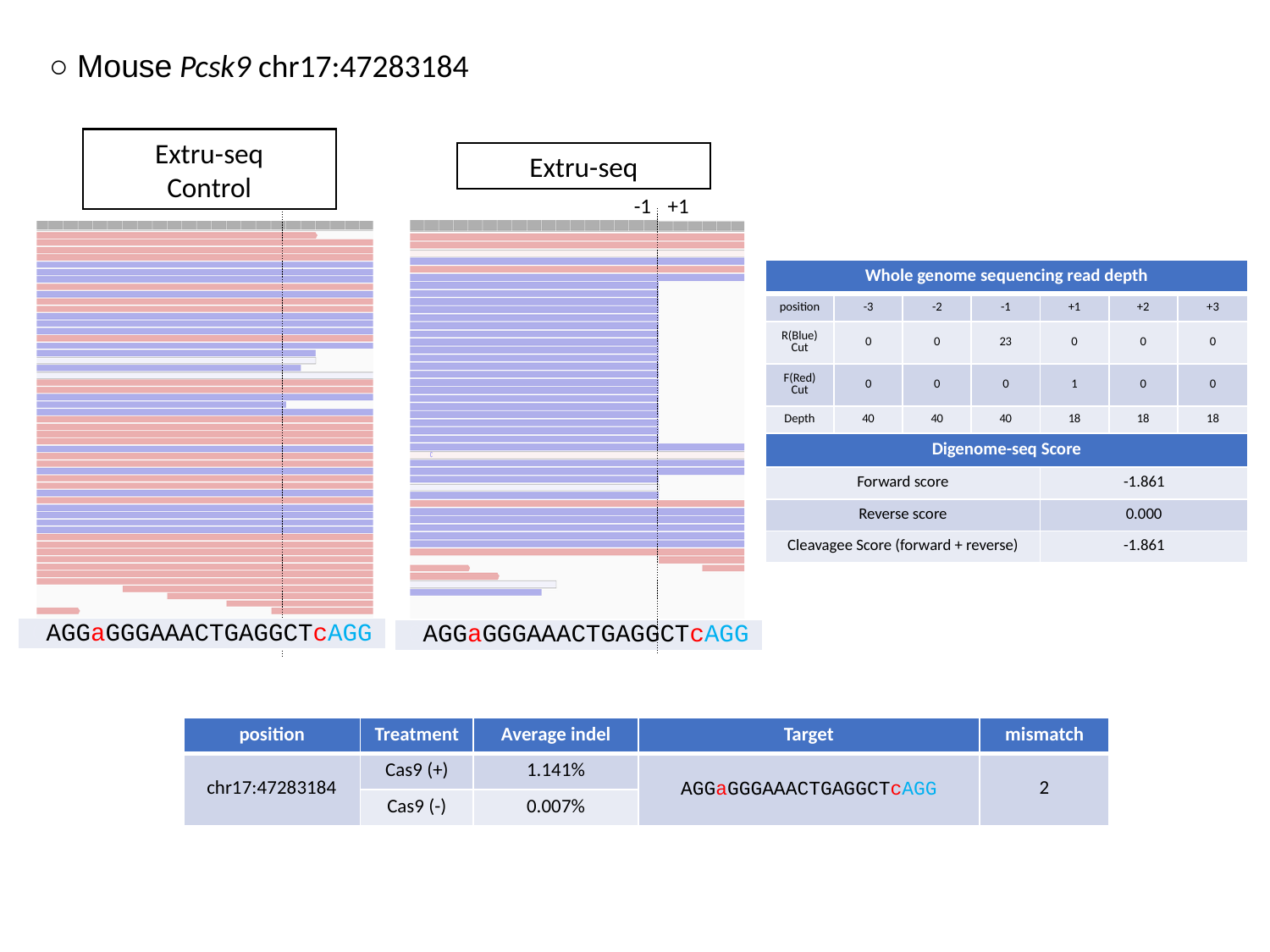

○ Mouse Pcsk9 chr17:47283184
Extru-seq
Control
Extru-seq
+1
-1
| Whole genome sequencing read depth | | | | | | |
| --- | --- | --- | --- | --- | --- | --- |
| position | -3 | -2 | -1 | +1 | +2 | +3 |
| R(Blue) Cut | 0 | 0 | 23 | 0 | 0 | 0 |
| F(Red) Cut | 0 | 0 | 0 | 1 | 0 | 0 |
| Depth | 40 | 40 | 40 | 18 | 18 | 18 |
| Digenome-seq Score | | | | | | |
| Forward score | | | | -1.861 | | |
| Reverse score | | | | 0.000 | | |
| Cleavagee Score (forward + reverse) | | | | -1.861 | | |
| CAGGaGGGAAACTGAGGCTcAGG |
| --- |
| CAGGaGGGAAACTGAGGCTcAGG |
| --- |
| CAGGaGGGAAACTGAGGCTcAGG |
| --- |
| position | Treatment | Average indel | Target | mismatch |
| --- | --- | --- | --- | --- |
| chr17:47283184 | Cas9 (+) | 1.141% | AGGaGGGAAACTGAGGCTcAGG | 2 |
| | Cas9 (-) | 0.007% | | |

## Slide 14
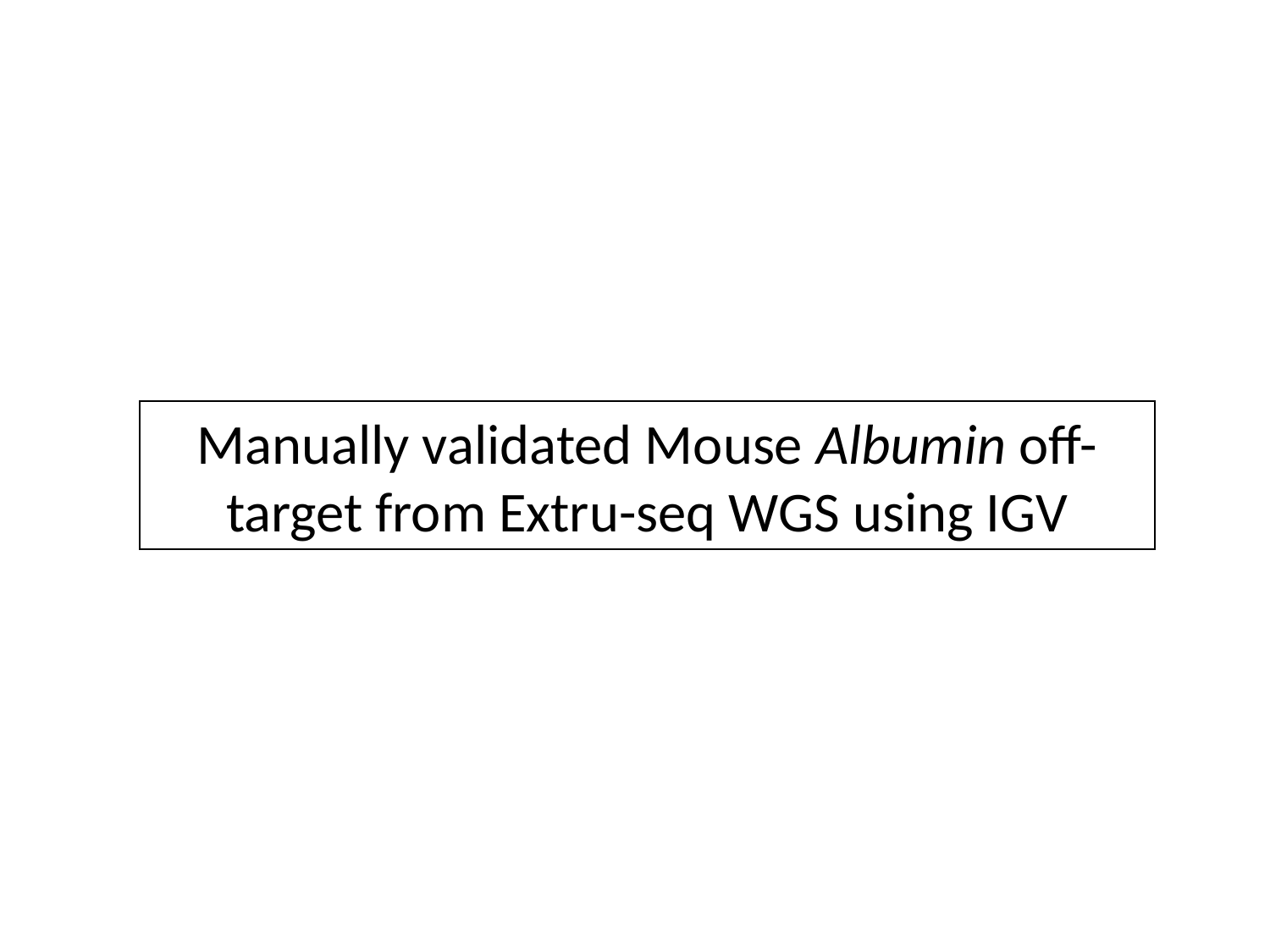

Manually validated Mouse Albumin off-target from Extru-seq WGS using IGV

## Slide 15
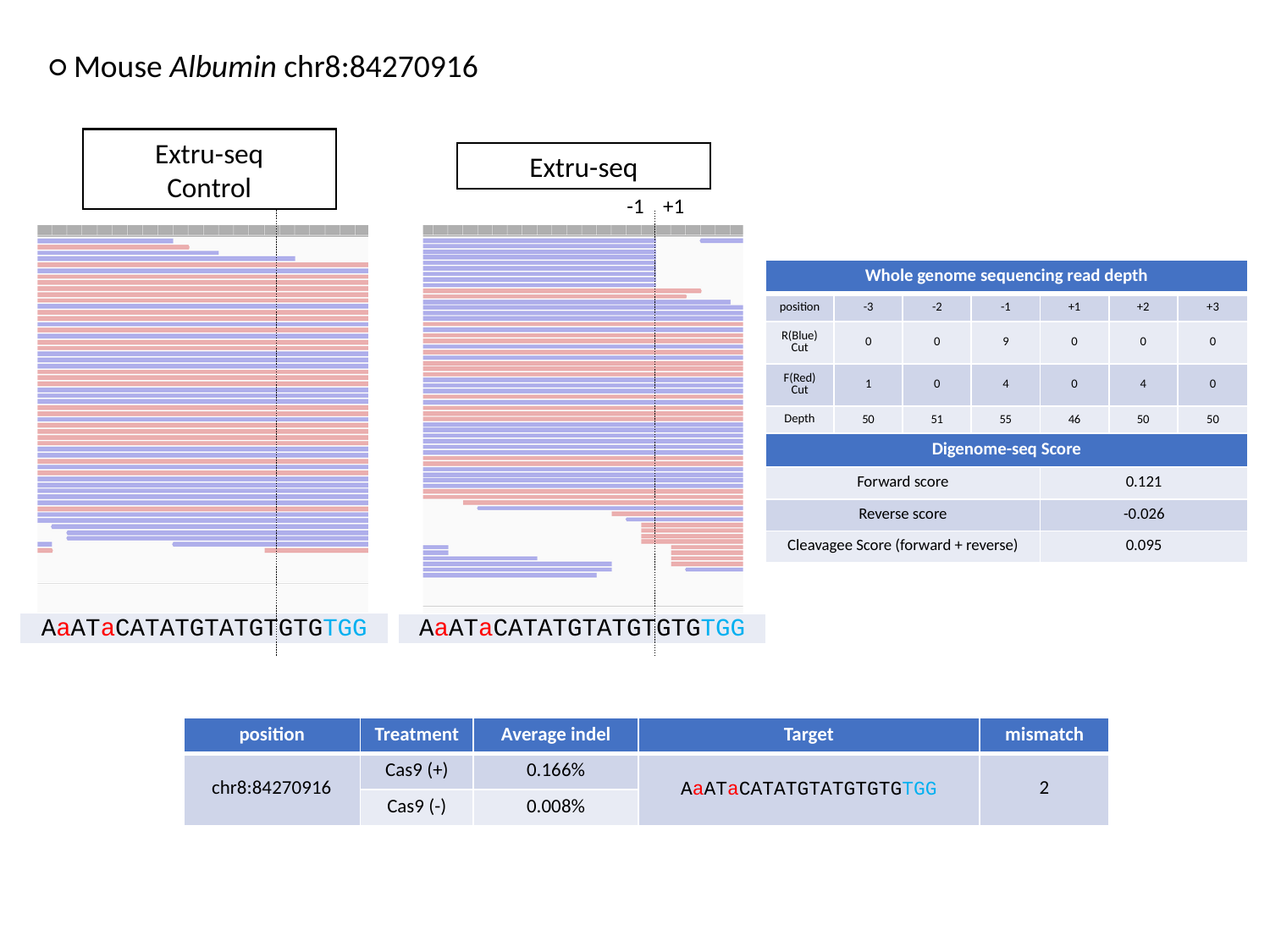

○ Mouse Albumin chr8:84270916
Extru-seq
Control
Extru-seq
+1
-1
| Whole genome sequencing read depth | | | | | | |
| --- | --- | --- | --- | --- | --- | --- |
| position | -3 | -2 | -1 | +1 | +2 | +3 |
| R(Blue) Cut | 0 | 0 | 9 | 0 | 0 | 0 |
| F(Red) Cut | 1 | 0 | 4 | 0 | 4 | 0 |
| Depth | 50 | 51 | 55 | 46 | 50 | 50 |
| Digenome-seq Score | | | | | | |
| Forward score | | | | 0.121 | | |
| Reverse score | | | | -0.026 | | |
| Cleavagee Score (forward + reverse) | | | | 0.095 | | |
| AaATaCATATGTATGTGTGTGG |
| --- |
| AaATaCATATGTATGTGTGTGG |
| --- |
| position | Treatment | Average indel | Target | mismatch |
| --- | --- | --- | --- | --- |
| chr8:84270916 | Cas9 (+) | 0.166% | AaATaCATATGTATGTGTGTGG | 2 |
| | Cas9 (-) | 0.008% | | |

## Slide 16
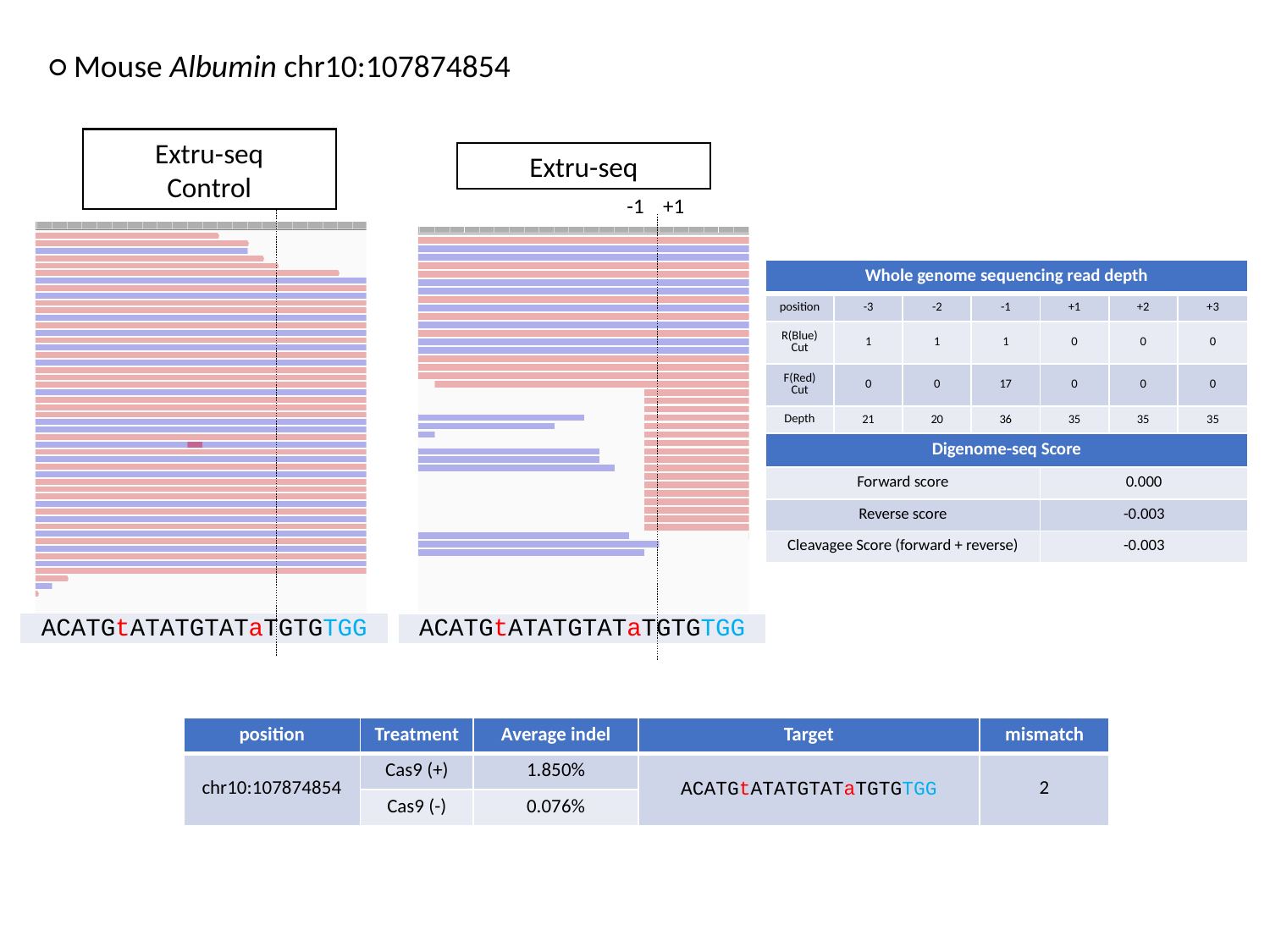

○ Mouse Albumin chr10:107874854
Extru-seq
Control
Extru-seq
+1
-1
| Whole genome sequencing read depth | | | | | | |
| --- | --- | --- | --- | --- | --- | --- |
| position | -3 | -2 | -1 | +1 | +2 | +3 |
| R(Blue) Cut | 1 | 1 | 1 | 0 | 0 | 0 |
| F(Red) Cut | 0 | 0 | 17 | 0 | 0 | 0 |
| Depth | 21 | 20 | 36 | 35 | 35 | 35 |
| Digenome-seq Score | | | | | | |
| Forward score | | | | 0.000 | | |
| Reverse score | | | | -0.003 | | |
| Cleavagee Score (forward + reverse) | | | | -0.003 | | |
| ACATGtATATGTATaTGTGTGG |
| --- |
| ACATGtATATGTATaTGTGTGG |
| --- |
| position | Treatment | Average indel | Target | mismatch |
| --- | --- | --- | --- | --- |
| chr10:107874854 | Cas9 (+) | 1.850% | ACATGtATATGTATaTGTGTGG | 2 |
| | Cas9 (-) | 0.076% | | |

## Slide 17
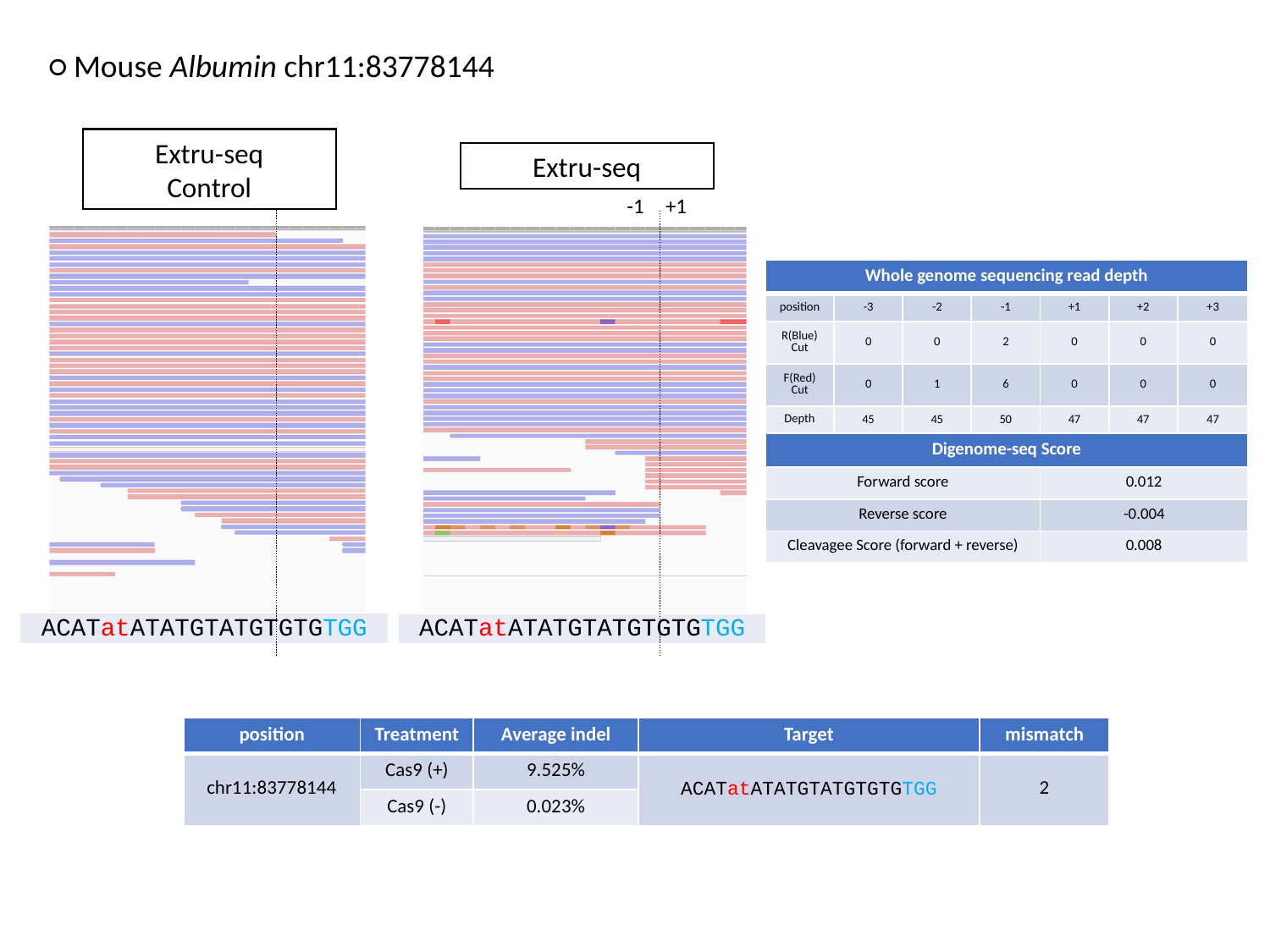

○ Mouse Albumin chr11:83778144
Extru-seq
Control
Extru-seq
+1
-1
| Whole genome sequencing read depth | | | | | | |
| --- | --- | --- | --- | --- | --- | --- |
| position | -3 | -2 | -1 | +1 | +2 | +3 |
| R(Blue) Cut | 0 | 0 | 2 | 0 | 0 | 0 |
| F(Red) Cut | 0 | 1 | 6 | 0 | 0 | 0 |
| Depth | 45 | 45 | 50 | 47 | 47 | 47 |
| Digenome-seq Score | | | | | | |
| Forward score | | | | 0.012 | | |
| Reverse score | | | | -0.004 | | |
| Cleavagee Score (forward + reverse) | | | | 0.008 | | |
| ACATatATATGTATGTGTGTGG |
| --- |
| ACATatATATGTATGTGTGTGG |
| --- |
| position | Treatment | Average indel | Target | mismatch |
| --- | --- | --- | --- | --- |
| chr11:83778144 | Cas9 (+) | 9.525% | ACATatATATGTATGTGTGTGG | 2 |
| | Cas9 (-) | 0.023% | | |

## Slide 18
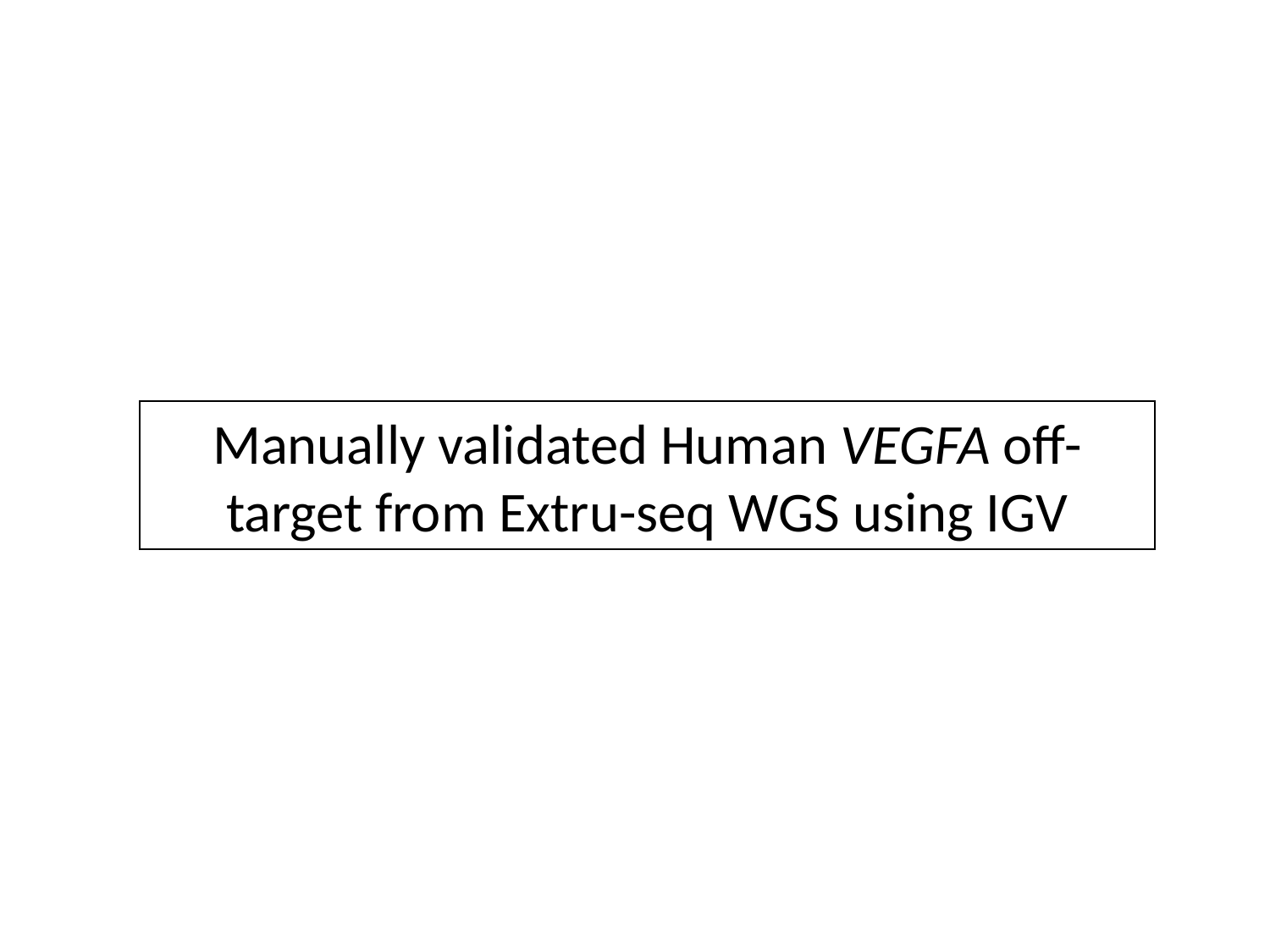

Manually validated Human VEGFA off-target from Extru-seq WGS using IGV

## Slide 19
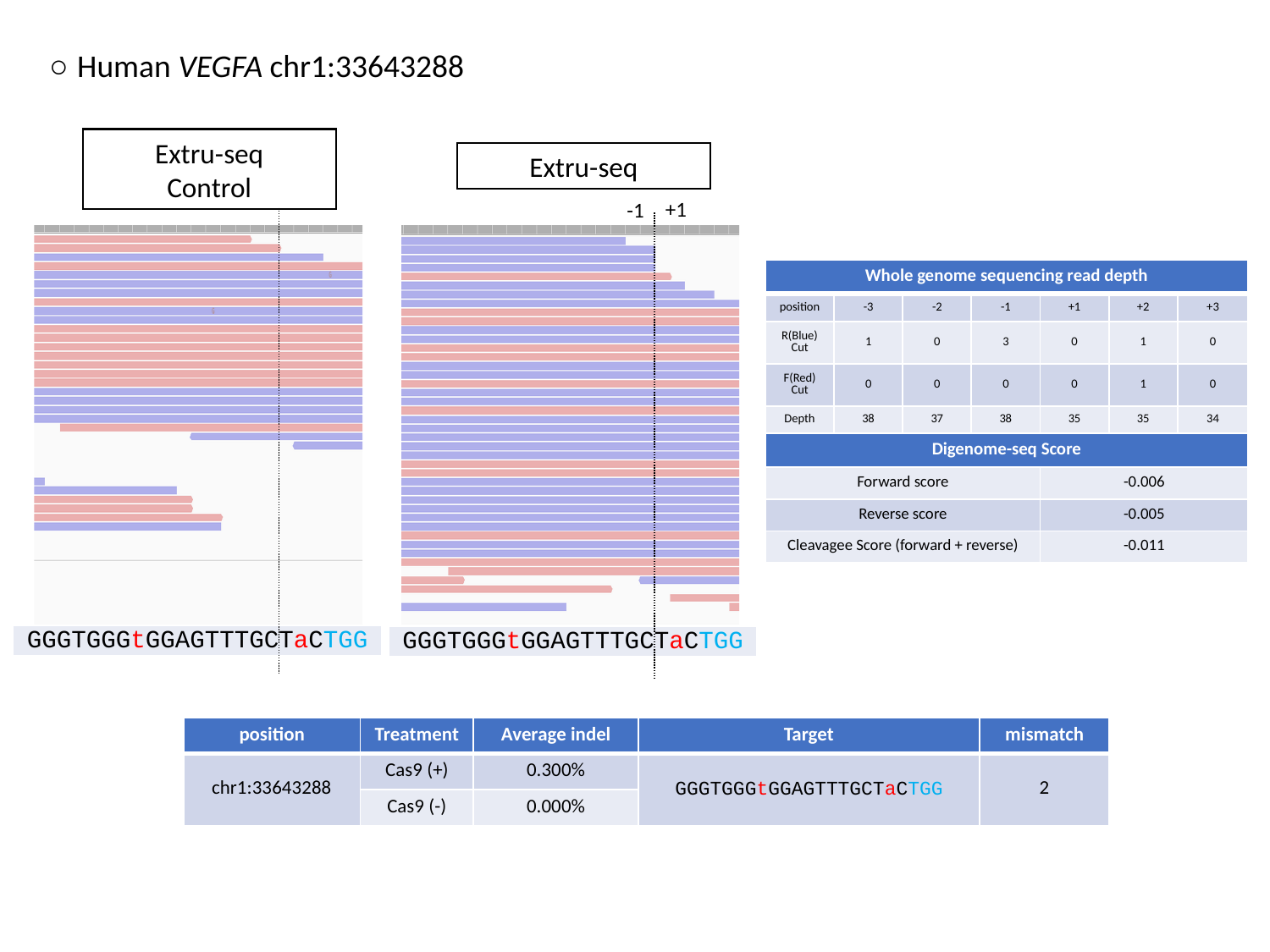

○ Human VEGFA chr1:33643288
Extru-seq
Control
Extru-seq
+1
-1
| Whole genome sequencing read depth | | | | | | |
| --- | --- | --- | --- | --- | --- | --- |
| position | -3 | -2 | -1 | +1 | +2 | +3 |
| R(Blue) Cut | 1 | 0 | 3 | 0 | 1 | 0 |
| F(Red) Cut | 0 | 0 | 0 | 0 | 1 | 0 |
| Depth | 38 | 37 | 38 | 35 | 35 | 34 |
| Digenome-seq Score | | | | | | |
| Forward score | | | | -0.006 | | |
| Reverse score | | | | -0.005 | | |
| Cleavagee Score (forward + reverse) | | | | -0.011 | | |
| GGGTGGGtGGAGTTTGCTaCTGG |
| --- |
| GGGTGGGtGGAGTTTGCTaCTGG |
| --- |
| position | Treatment | Average indel | Target | mismatch |
| --- | --- | --- | --- | --- |
| chr1:33643288 | Cas9 (+) | 0.300% | GGGTGGGtGGAGTTTGCTaCTGG | 2 |
| | Cas9 (-) | 0.000% | | |

## Slide 20
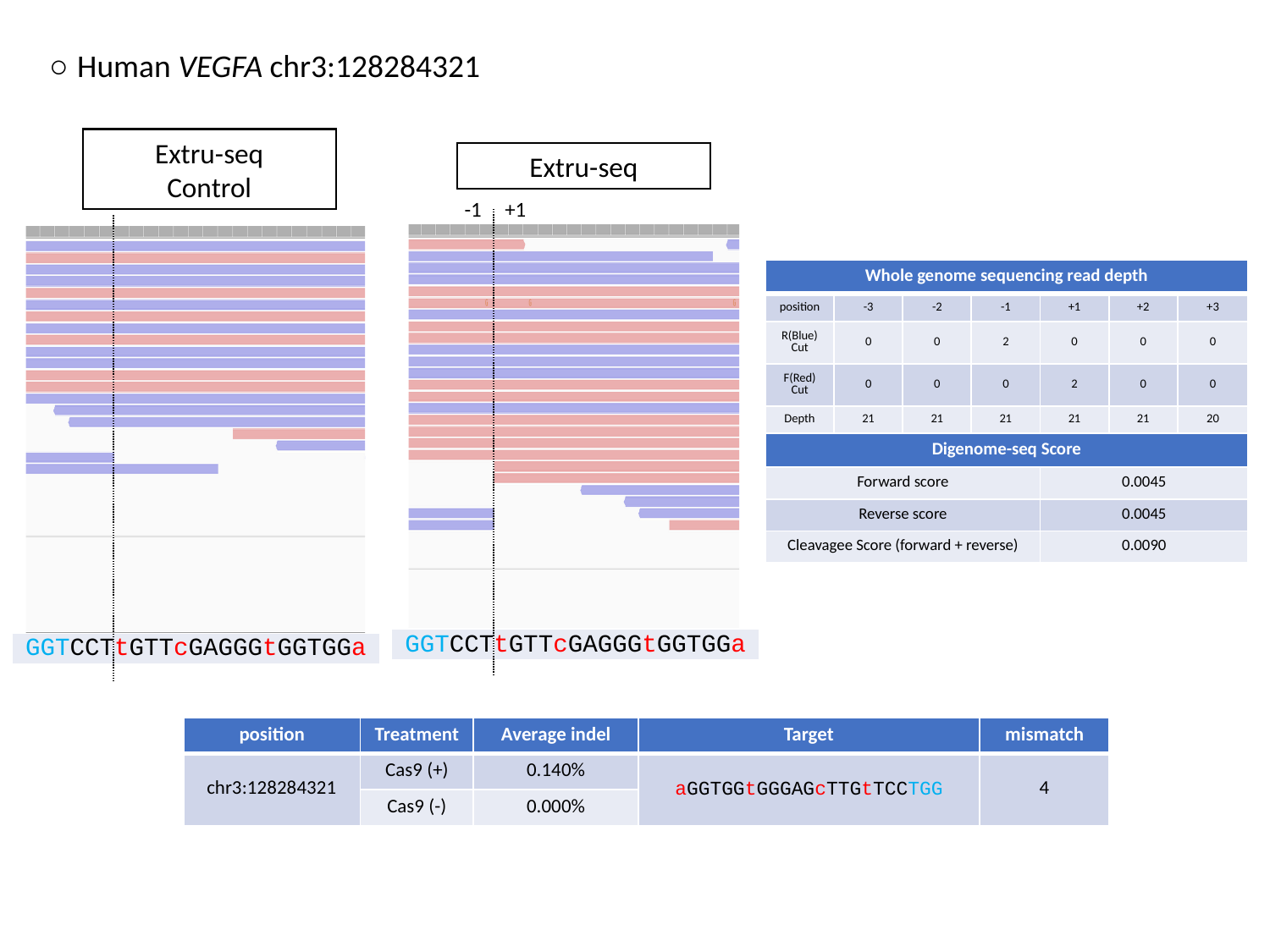

○ Human VEGFA chr3:128284321
Extru-seq
Control
Extru-seq
-1
+1
| Whole genome sequencing read depth | | | | | | |
| --- | --- | --- | --- | --- | --- | --- |
| position | -3 | -2 | -1 | +1 | +2 | +3 |
| R(Blue) Cut | 0 | 0 | 2 | 0 | 0 | 0 |
| F(Red) Cut | 0 | 0 | 0 | 2 | 0 | 0 |
| Depth | 21 | 21 | 21 | 21 | 21 | 20 |
| Digenome-seq Score | | | | | | |
| Forward score | | | | 0.0045 | | |
| Reverse score | | | | 0.0045 | | |
| Cleavagee Score (forward + reverse) | | | | 0.0090 | | |
| GGTCCTtGTTcGAGGGtGGTGGa |
| --- |
| GGTCCTtGTTcGAGGGtGGTGGa |
| --- |
| position | Treatment | Average indel | Target | mismatch |
| --- | --- | --- | --- | --- |
| chr3:128284321 | Cas9 (+) | 0.140% | aGGTGGtGGGAGcTTGtTCCTGG | 4 |
| | Cas9 (-) | 0.000% | | |

## Slide 21
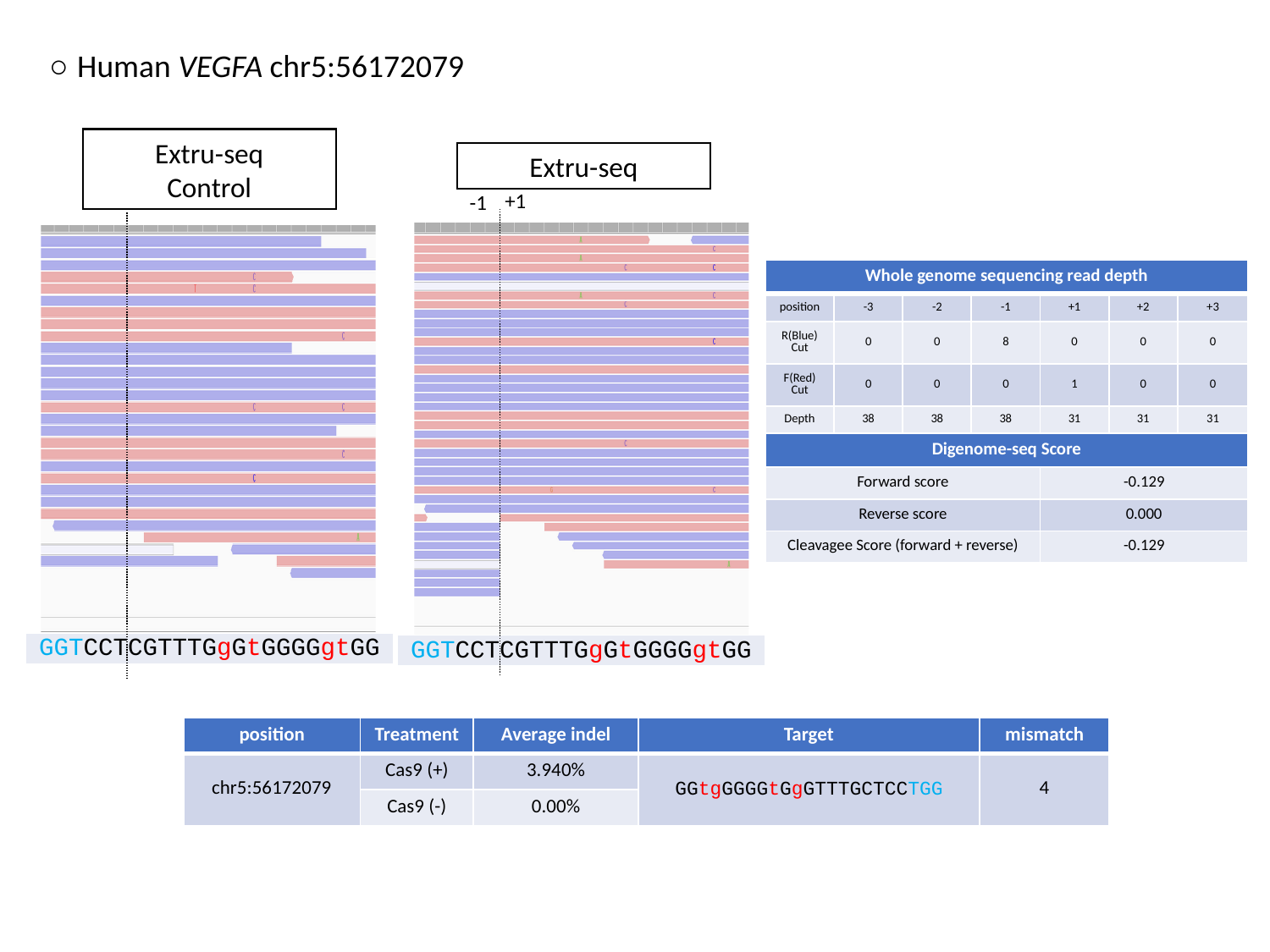

○ Human VEGFA chr5:56172079
Extru-seq
Control
Extru-seq
+1
-1
| Whole genome sequencing read depth | | | | | | |
| --- | --- | --- | --- | --- | --- | --- |
| position | -3 | -2 | -1 | +1 | +2 | +3 |
| R(Blue) Cut | 0 | 0 | 8 | 0 | 0 | 0 |
| F(Red) Cut | 0 | 0 | 0 | 1 | 0 | 0 |
| Depth | 38 | 38 | 38 | 31 | 31 | 31 |
| Digenome-seq Score | | | | | | |
| Forward score | | | | -0.129 | | |
| Reverse score | | | | 0.000 | | |
| Cleavagee Score (forward + reverse) | | | | -0.129 | | |
| GGTCCTCGTTTGgGtGGGGgtGG |
| --- |
| GGTCCTCGTTTGgGtGGGGgtGG |
| --- |
| position | Treatment | Average indel | Target | mismatch |
| --- | --- | --- | --- | --- |
| chr5:56172079 | Cas9 (+) | 3.940% | GGtgGGGGtGgGTTTGCTCCTGG | 4 |
| | Cas9 (-) | 0.00% | | |

## Slide 22
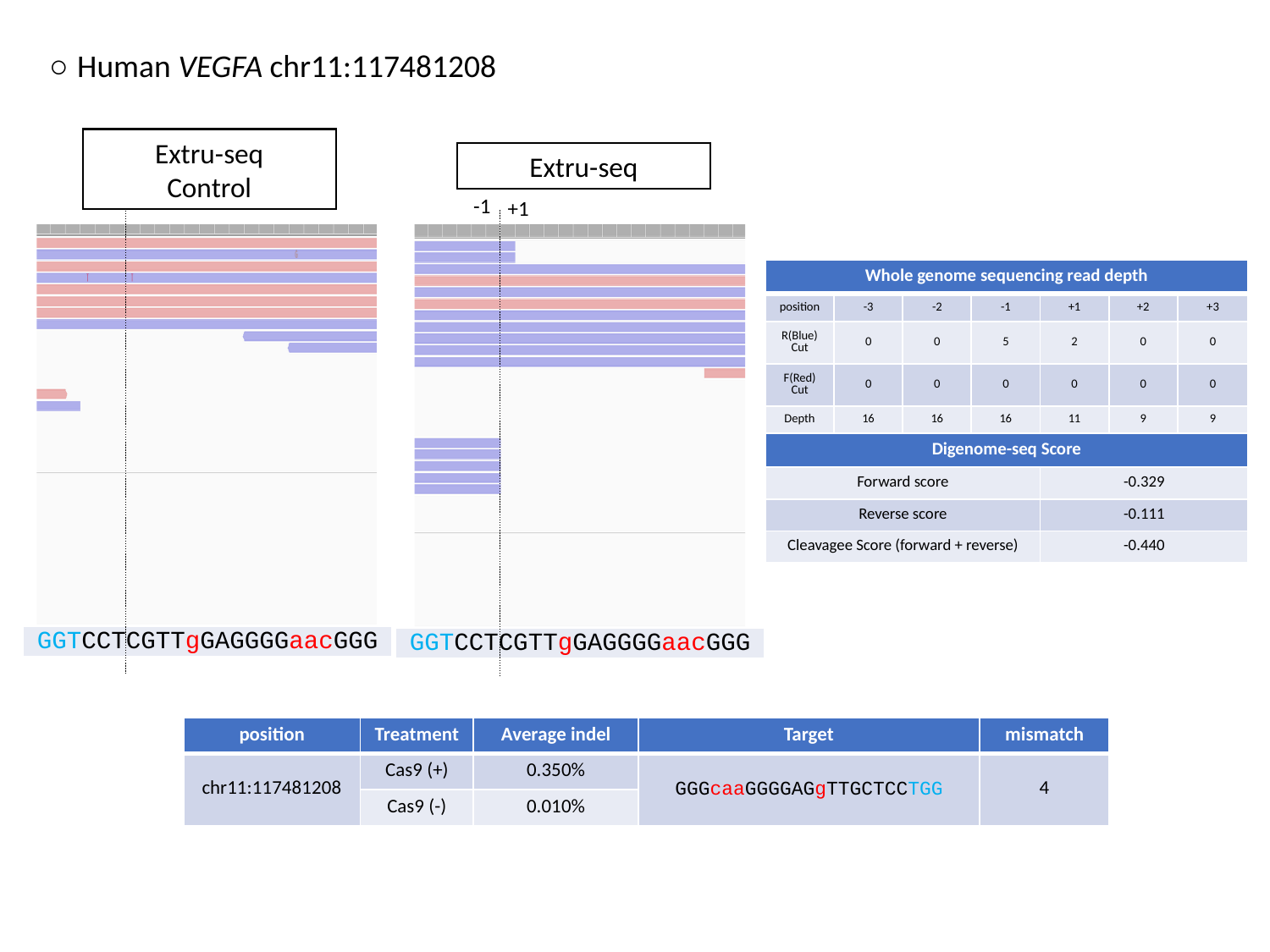

○ Human VEGFA chr11:117481208
Extru-seq
Control
Extru-seq
-1
+1
| Whole genome sequencing read depth | | | | | | |
| --- | --- | --- | --- | --- | --- | --- |
| position | -3 | -2 | -1 | +1 | +2 | +3 |
| R(Blue) Cut | 0 | 0 | 5 | 2 | 0 | 0 |
| F(Red) Cut | 0 | 0 | 0 | 0 | 0 | 0 |
| Depth | 16 | 16 | 16 | 11 | 9 | 9 |
| Digenome-seq Score | | | | | | |
| Forward score | | | | -0.329 | | |
| Reverse score | | | | -0.111 | | |
| Cleavagee Score (forward + reverse) | | | | -0.440 | | |
| GGTCCTCGTTgGAGGGGaacGGG |
| --- |
| GGTCCTCGTTgGAGGGGaacGGG |
| --- |
| position | Treatment | Average indel | Target | mismatch |
| --- | --- | --- | --- | --- |
| chr11:117481208 | Cas9 (+) | 0.350% | GGGcaaGGGGAGgTTGCTCCTGG | 4 |
| | Cas9 (-) | 0.010% | | |

## Slide 23
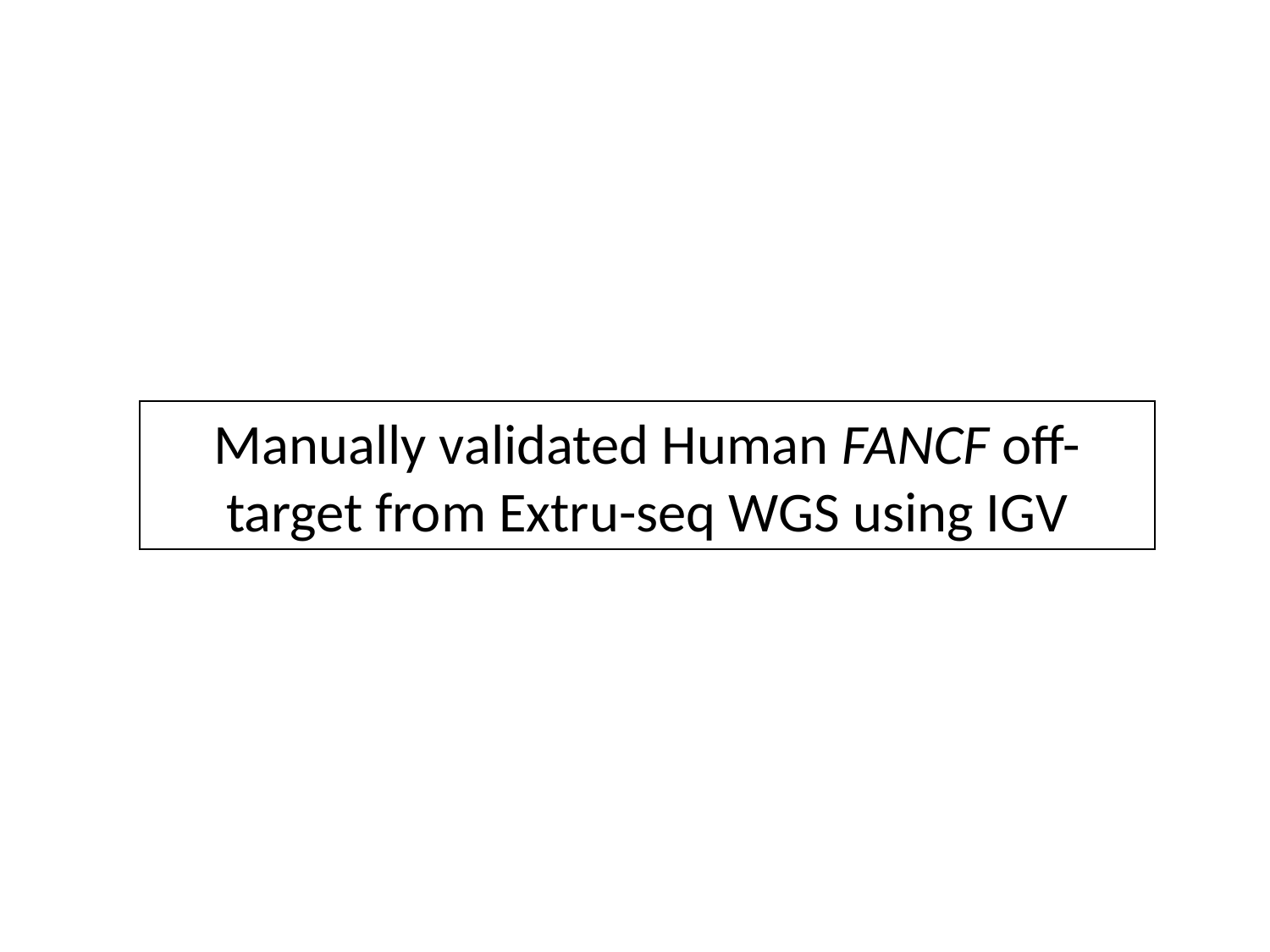

Manually validated Human FANCF off-target from Extru-seq WGS using IGV

## Slide 24
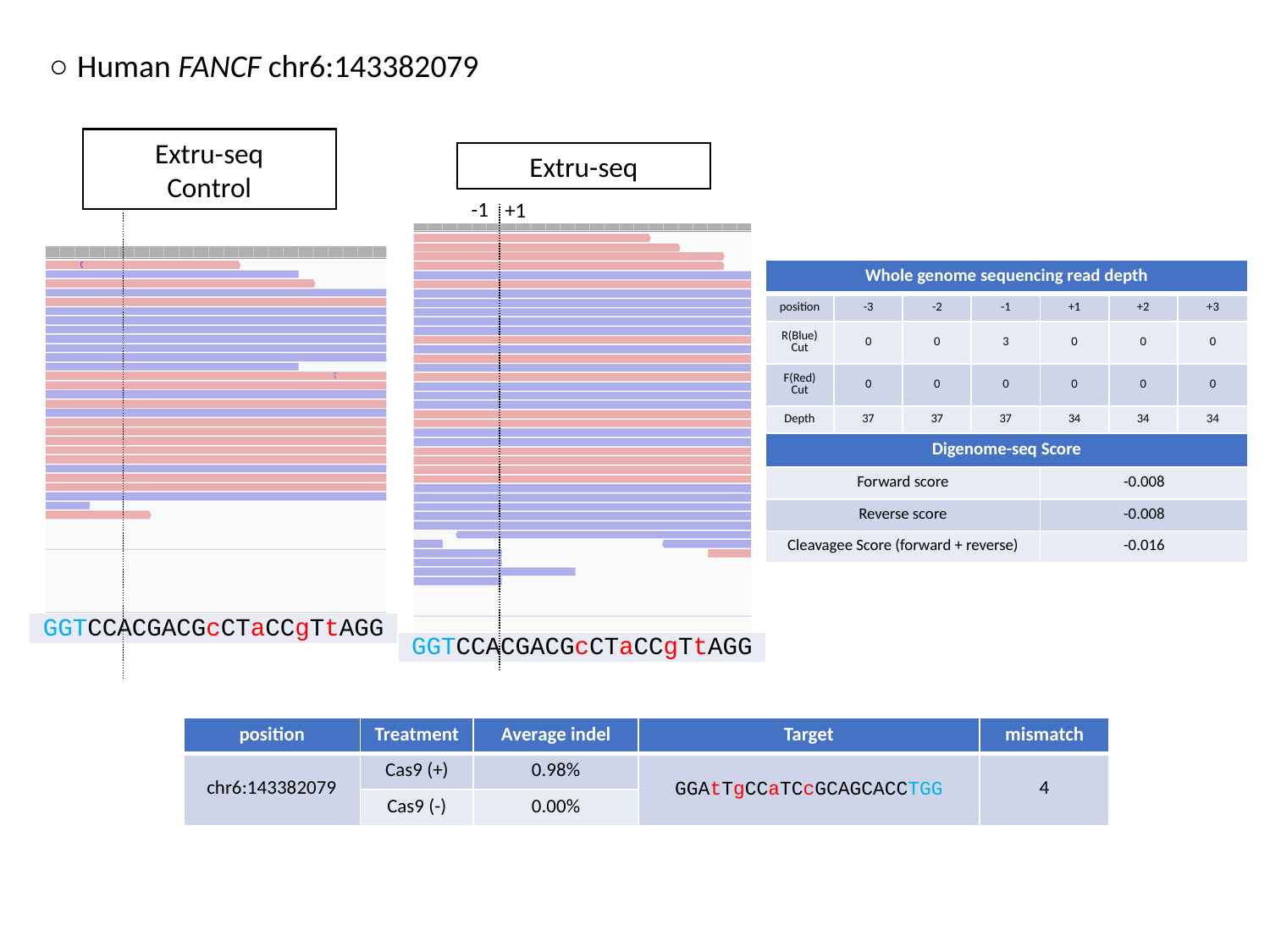

○ Human FANCF chr6:143382079
Extru-seq
Control
Extru-seq
-1
+1
| Whole genome sequencing read depth | | | | | | |
| --- | --- | --- | --- | --- | --- | --- |
| position | -3 | -2 | -1 | +1 | +2 | +3 |
| R(Blue) Cut | 0 | 0 | 3 | 0 | 0 | 0 |
| F(Red) Cut | 0 | 0 | 0 | 0 | 0 | 0 |
| Depth | 37 | 37 | 37 | 34 | 34 | 34 |
| Digenome-seq Score | | | | | | |
| Forward score | | | | -0.008 | | |
| Reverse score | | | | -0.008 | | |
| Cleavagee Score (forward + reverse) | | | | -0.016 | | |
| GGTCCACGACGcCTaCCgTtAGG |
| --- |
| GGTCCACGACGcCTaCCgTtAGG |
| --- |
| position | Treatment | Average indel | Target | mismatch |
| --- | --- | --- | --- | --- |
| chr6:143382079 | Cas9 (+) | 0.98% | GGAtTgCCaTCcGCAGCACCTGG | 4 |
| | Cas9 (-) | 0.00% | | |

## Slide 25
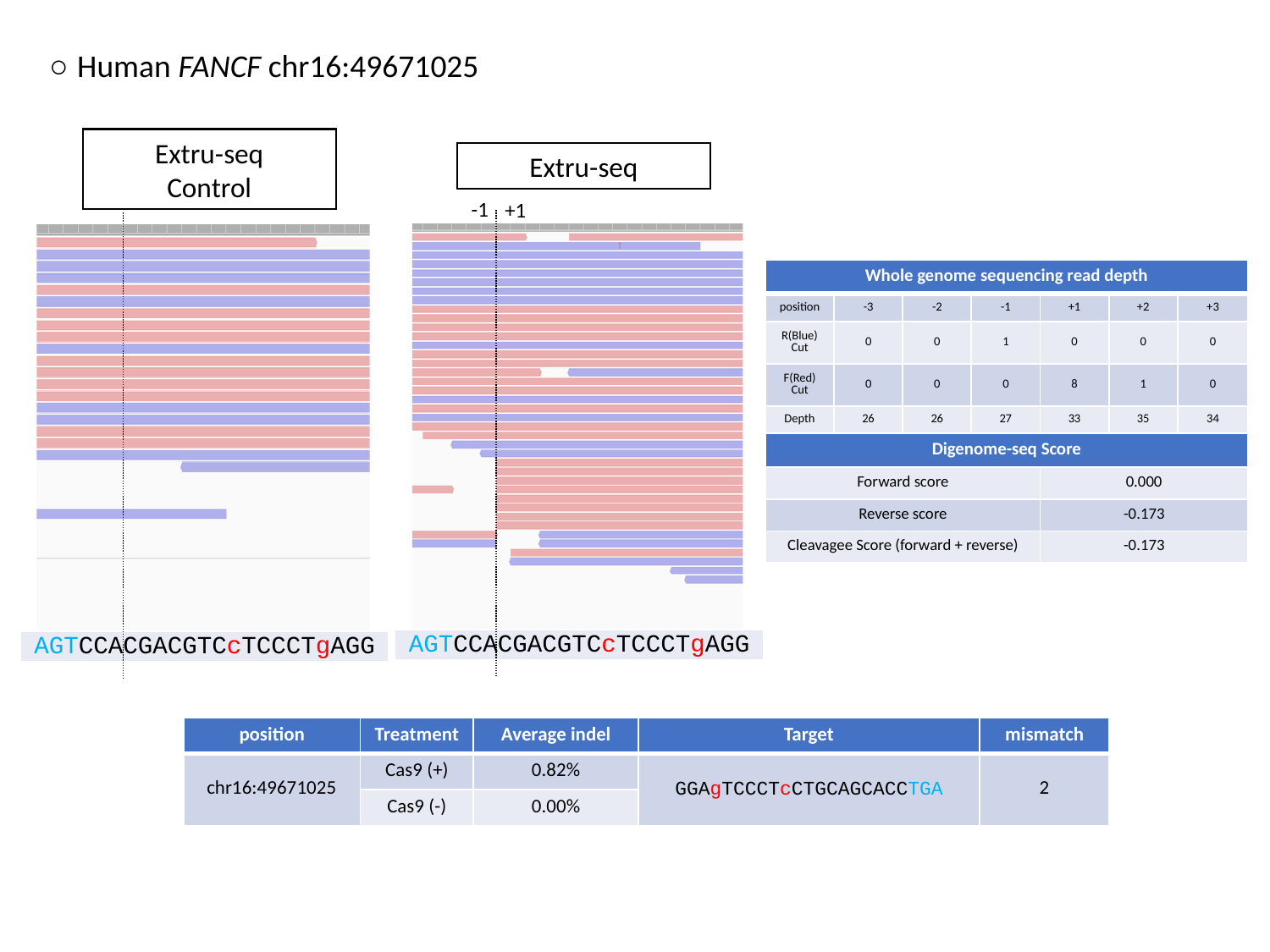

○ Human FANCF chr16:49671025
Extru-seq
Control
Extru-seq
-1
+1
| Whole genome sequencing read depth | | | | | | |
| --- | --- | --- | --- | --- | --- | --- |
| position | -3 | -2 | -1 | +1 | +2 | +3 |
| R(Blue) Cut | 0 | 0 | 1 | 0 | 0 | 0 |
| F(Red) Cut | 0 | 0 | 0 | 8 | 1 | 0 |
| Depth | 26 | 26 | 27 | 33 | 35 | 34 |
| Digenome-seq Score | | | | | | |
| Forward score | | | | 0.000 | | |
| Reverse score | | | | -0.173 | | |
| Cleavagee Score (forward + reverse) | | | | -0.173 | | |
| AGTCCACGACGTCcTCCCTgAGG |
| --- |
| AGTCCACGACGTCcTCCCTgAGG |
| --- |
| position | Treatment | Average indel | Target | mismatch |
| --- | --- | --- | --- | --- |
| chr16:49671025 | Cas9 (+) | 0.82% | GGAgTCCCTcCTGCAGCACCTGA | 2 |
| | Cas9 (-) | 0.00% | | |

## Slide 26
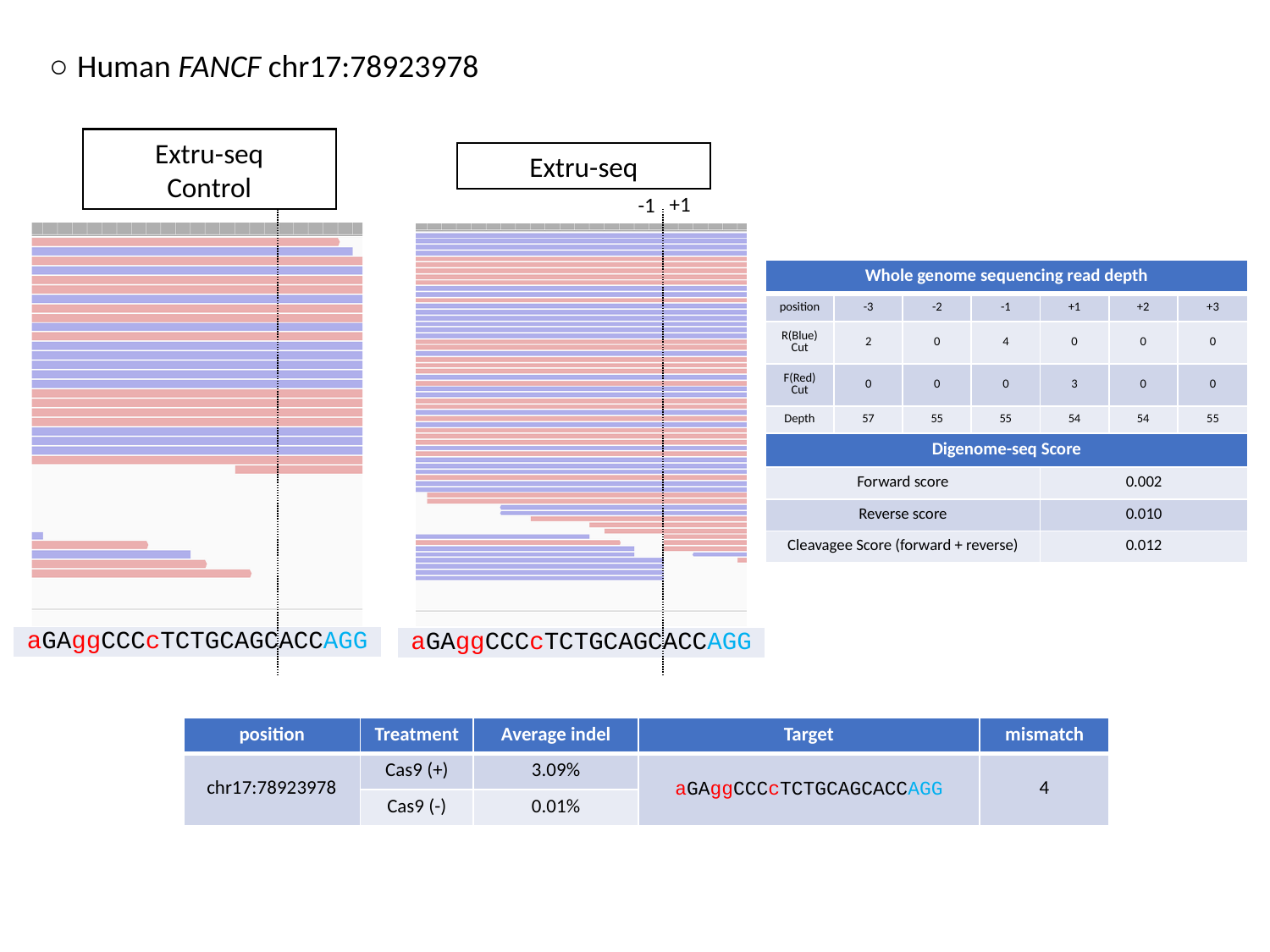

○ Human FANCF chr17:78923978
Extru-seq
Control
Extru-seq
+1
-1
| Whole genome sequencing read depth | | | | | | |
| --- | --- | --- | --- | --- | --- | --- |
| position | -3 | -2 | -1 | +1 | +2 | +3 |
| R(Blue) Cut | 2 | 0 | 4 | 0 | 0 | 0 |
| F(Red) Cut | 0 | 0 | 0 | 3 | 0 | 0 |
| Depth | 57 | 55 | 55 | 54 | 54 | 55 |
| Digenome-seq Score | | | | | | |
| Forward score | | | | 0.002 | | |
| Reverse score | | | | 0.010 | | |
| Cleavagee Score (forward + reverse) | | | | 0.012 | | |
| aGAggCCCcTCTGCAGCACCAGG |
| --- |
| aGAggCCCcTCTGCAGCACCAGG |
| --- |
| position | Treatment | Average indel | Target | mismatch |
| --- | --- | --- | --- | --- |
| chr17:78923978 | Cas9 (+) | 3.09% | aGAggCCCcTCTGCAGCACCAGG | 4 |
| | Cas9 (-) | 0.01% | | |

## Slide 27
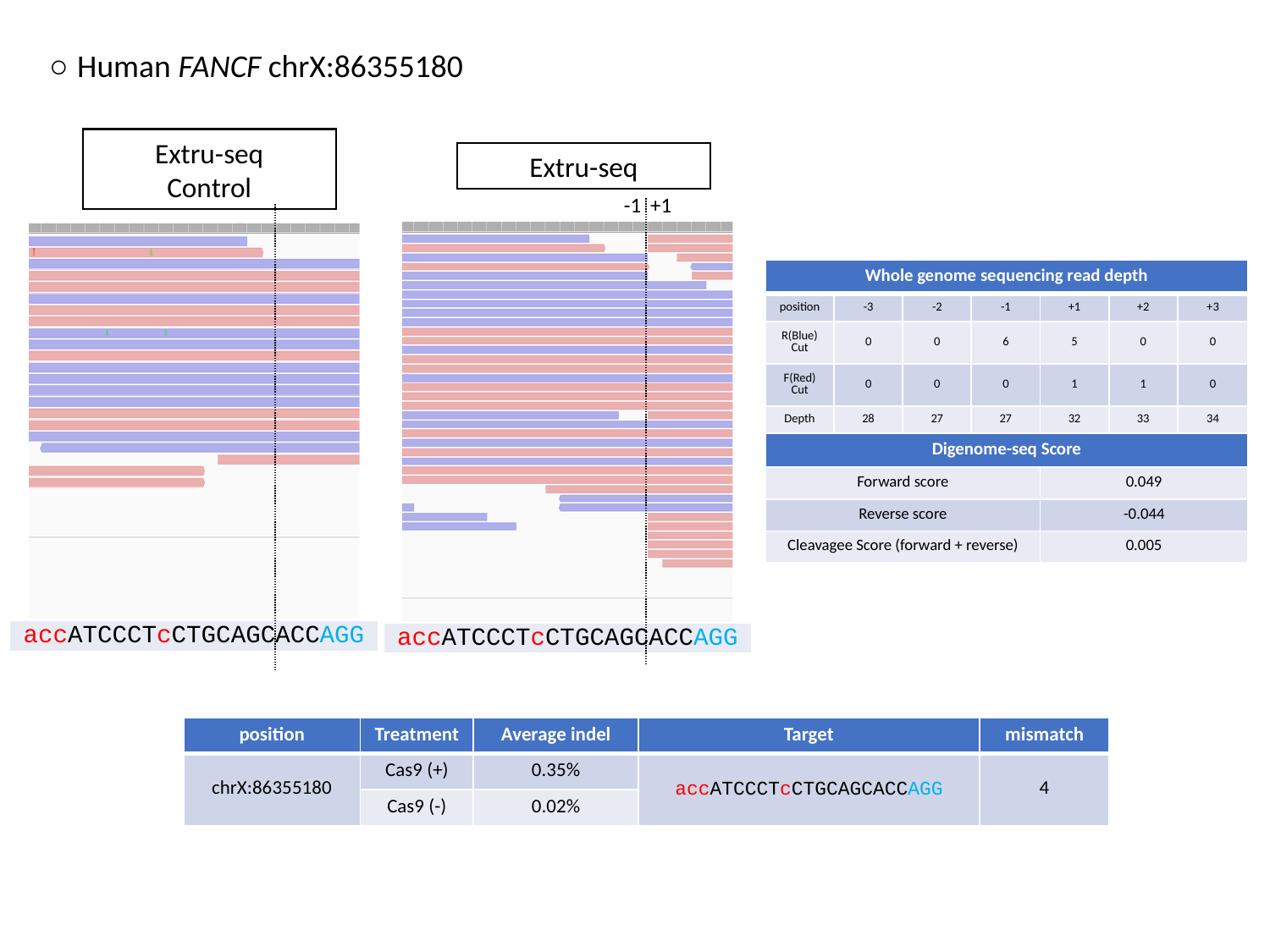

○ Human FANCF chrX:86355180
Extru-seq
Control
Extru-seq
-1
+1
| Whole genome sequencing read depth | | | | | | |
| --- | --- | --- | --- | --- | --- | --- |
| position | -3 | -2 | -1 | +1 | +2 | +3 |
| R(Blue) Cut | 0 | 0 | 6 | 5 | 0 | 0 |
| F(Red) Cut | 0 | 0 | 0 | 1 | 1 | 0 |
| Depth | 28 | 27 | 27 | 32 | 33 | 34 |
| Digenome-seq Score | | | | | | |
| Forward score | | | | 0.049 | | |
| Reverse score | | | | -0.044 | | |
| Cleavagee Score (forward + reverse) | | | | 0.005 | | |
| accATCCCTcCTGCAGCACCAGG |
| --- |
| accATCCCTcCTGCAGCACCAGG |
| --- |
| position | Treatment | Average indel | Target | mismatch |
| --- | --- | --- | --- | --- |
| chrX:86355180 | Cas9 (+) | 0.35% | accATCCCTcCTGCAGCACCAGG | 4 |
| | Cas9 (-) | 0.02% | | |
