# Supplementary material for: Extru-seq: a method for predicting genome-wide Cas9 off-target sites with advantages of both cell-based and in vitro approaches
Source: Genome Biol. 2023 Jan 10;24:4. doi: 10.1186/s13059-022-02842-4 (PMC9832775; doi:10.1186/s13059-022-02842-4)
Supplement: Supplementary file 4 — Additional file 4: Table S3. Manually excluded false positive off-target sites from Digenome-seq and Extru-seq WGS data visualized using IGV. [file 13059_2022_2842_MOESM4_ESM.pptx]

## Slide 1
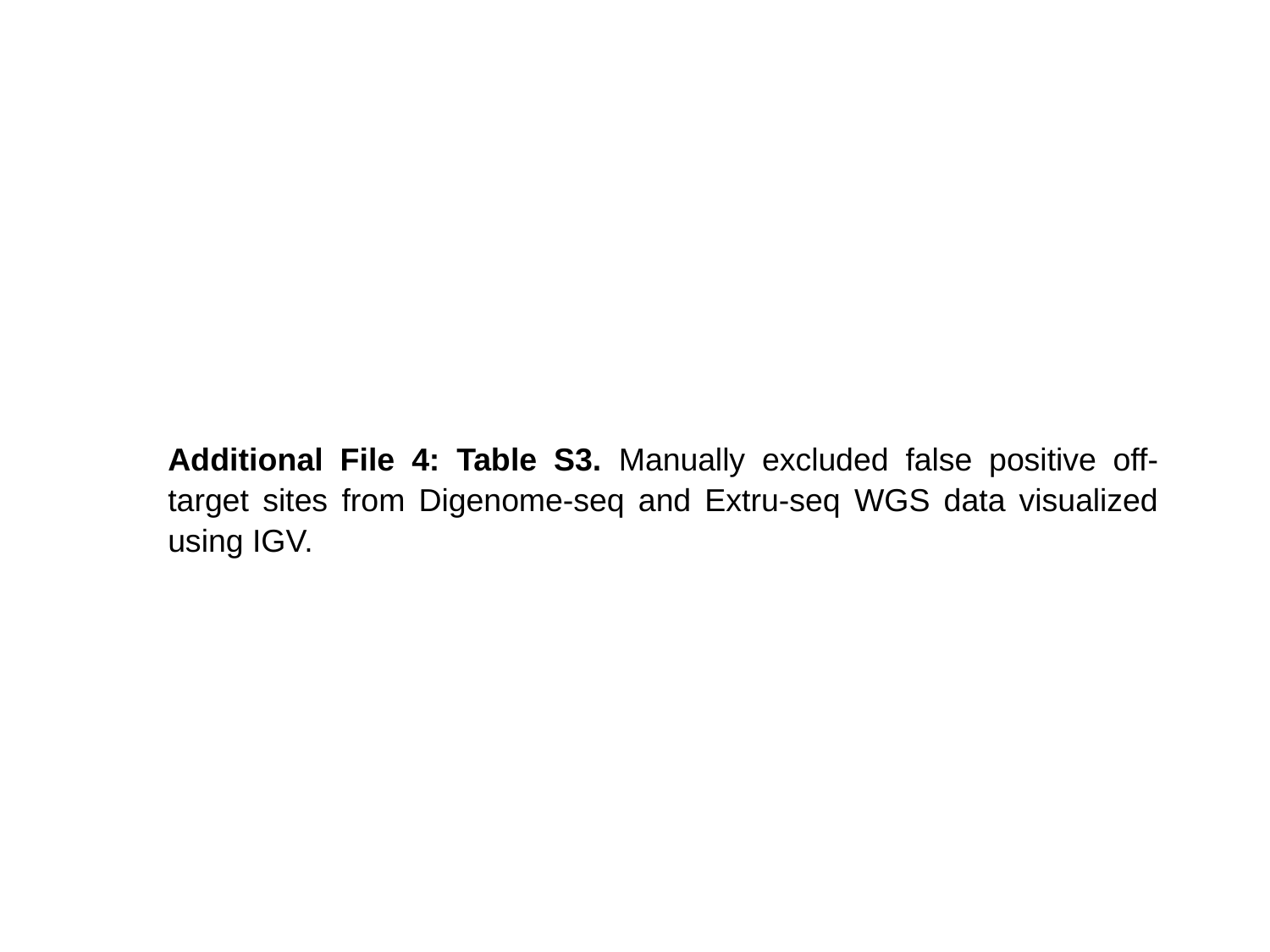

Additional File 4: Table S3. Manually excluded false positive off-target sites from Digenome-seq and Extru-seq WGS data visualized using IGV.

## Slide 2
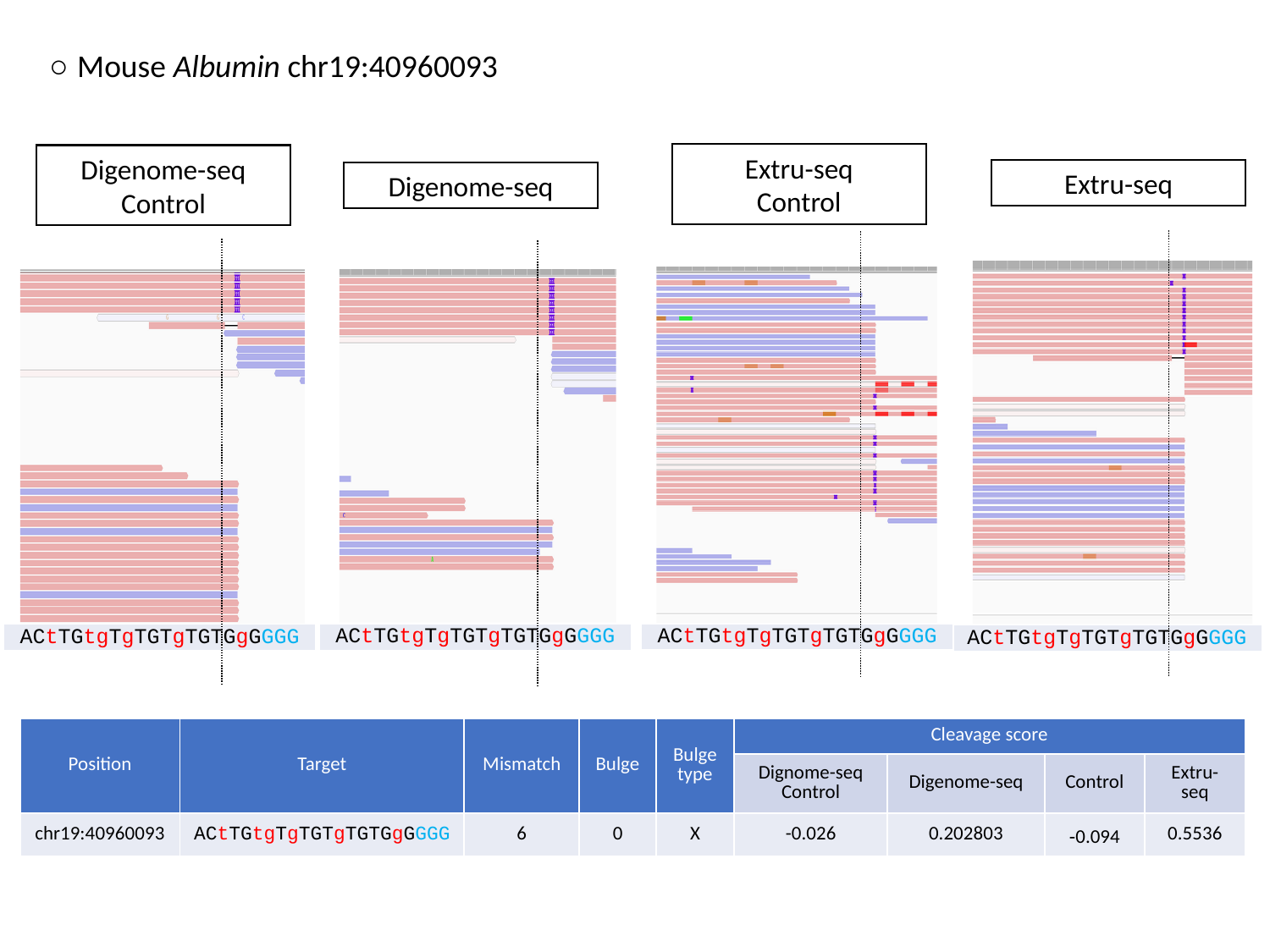

○ Mouse Albumin chr19:40960093
Extru-seq
Control
Digenome-seq
Control
Extru-seq
Digenome-seq
| ACtTGtgTgTGTgTGTGgGGGG |
| --- |
| ACtTGtgTgTGTgTGTGgGGGG |
| --- |
| ACtTGtgTgTGTgTGTGgGGGG |
| --- |
| ACtTGtgTgTGTgTGTGgGGGG |
| --- |
| Position | Target | Mismatch | Bulge | Bulge type | Cleavage score | | | |
| --- | --- | --- | --- | --- | --- | --- | --- | --- |
| | | | | | Dignome-seq Control | Digenome-seq | Control | Extru-seq |
| chr19:40960093 | ACtTGtgTgTGTgTGTGgGGGG | 6 | 0 | X | -0.026 | 0.202803 | -0.094 | 0.5536 |

## Slide 3
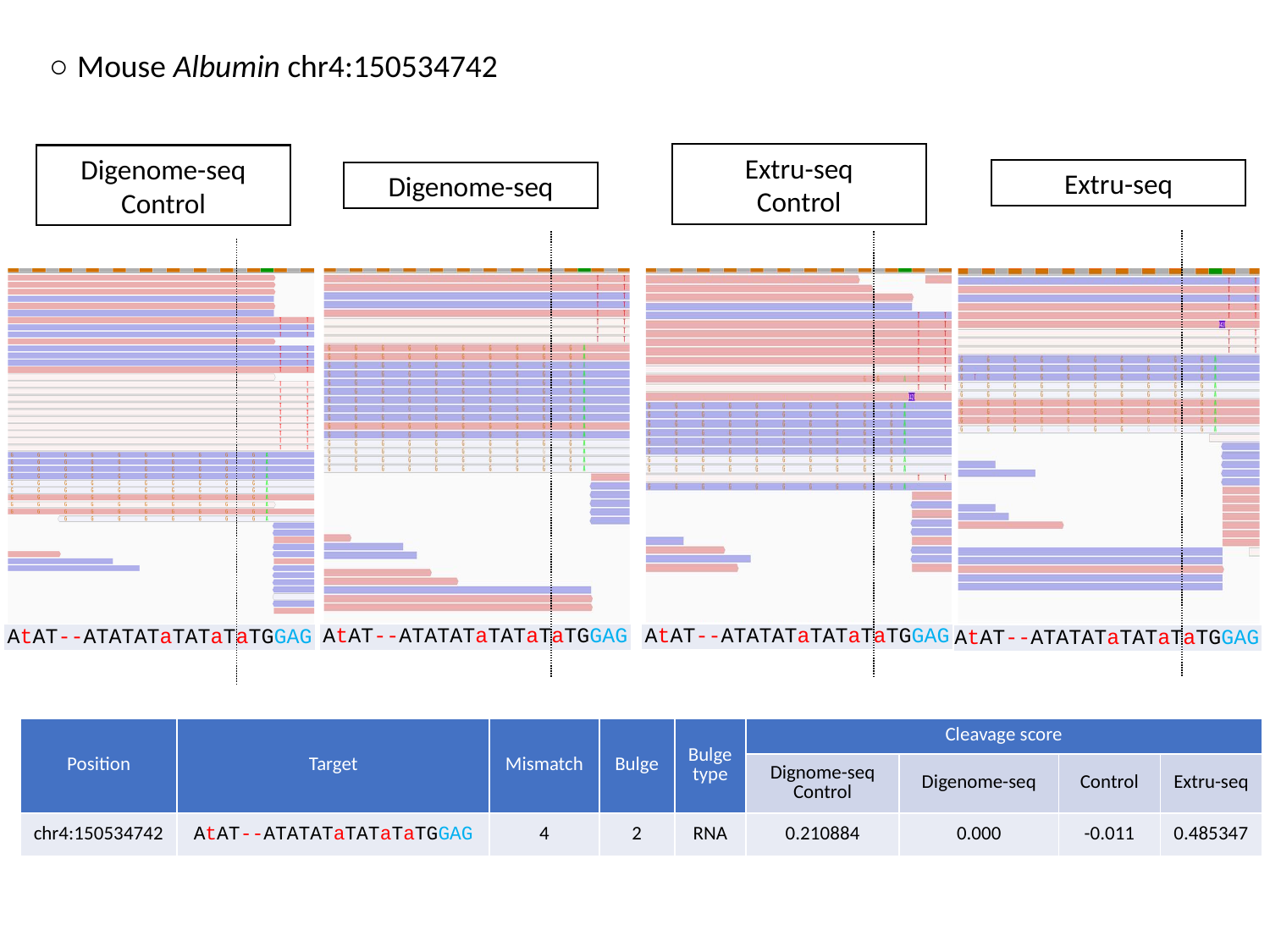

○ Mouse Albumin chr4:150534742
Extru-seq
Control
Digenome-seq
Control
Extru-seq
Digenome-seq
| AtAT--ATATATaTATaTaTGGAG |
| --- |
| AtAT--ATATATaTATaTaTGGAG |
| --- |
| AtAT--ATATATaTATaTaTGGAG |
| --- |
| AtAT--ATATATaTATaTaTGGAG |
| --- |
| Position | Target | Mismatch | Bulge | Bulge type | Cleavage score | | | |
| --- | --- | --- | --- | --- | --- | --- | --- | --- |
| | | | | | Dignome-seq Control | Digenome-seq | Control | Extru-seq |
| chr4:150534742 | AtAT--ATATATaTATaTaTGGAG | 4 | 2 | RNA | 0.210884 | 0.000 | -0.011 | 0.485347 |

## Slide 4
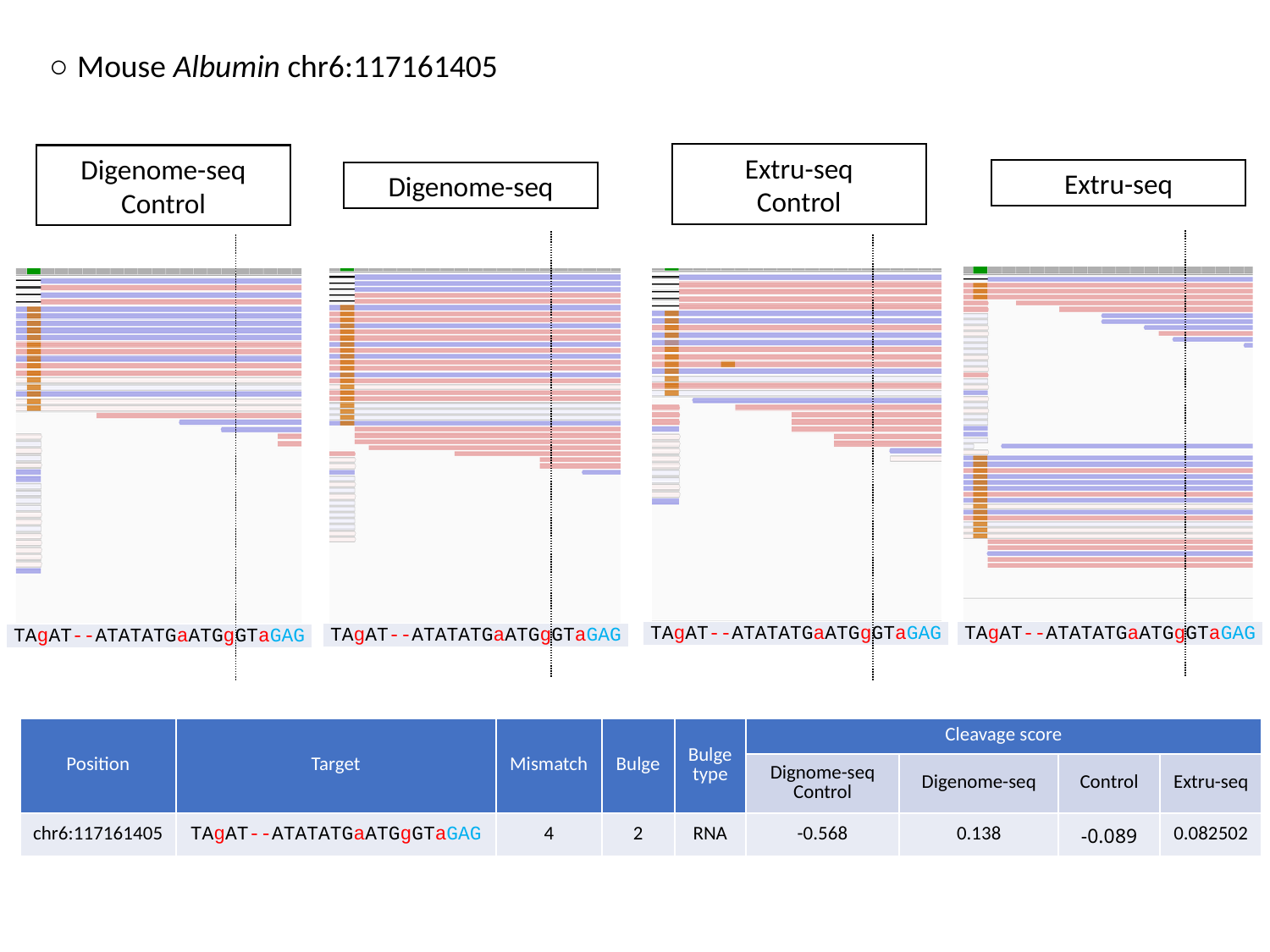

○ Mouse Albumin chr6:117161405
Extru-seq
Control
Digenome-seq
Control
Extru-seq
Digenome-seq
| TAgAT--ATATATGaATGgGTaGAG |
| --- |
| TAgAT--ATATATGaATGgGTaGAG |
| --- |
| TAgAT--ATATATGaATGgGTaGAG |
| --- |
| TAgAT--ATATATGaATGgGTaGAG |
| --- |
| Position | Target | Mismatch | Bulge | Bulge type | Cleavage score | | | |
| --- | --- | --- | --- | --- | --- | --- | --- | --- |
| | | | | | Dignome-seq Control | Digenome-seq | Control | Extru-seq |
| chr6:117161405 | TAgAT--ATATATGaATGgGTaGAG | 4 | 2 | RNA | -0.568 | 0.138 | -0.089 | 0.082502 |
