# Supplementary material for: Extru-seq: a method for predicting genome-wide Cas9 off-target sites with advantages of both cell-based and in vitro approaches
Source: Genome Biol. 2023 Jan 10;24:4. doi: 10.1186/s13059-022-02842-4 (PMC9832775; doi:10.1186/s13059-022-02842-4)
Supplement: Supplementary file 6 — Additional file 6. Review history. [file 13059_2022_2842_MOESM6_ESM.docx]

Review History

**First round of review**

**Reviewer 1**

**Were you able to assess all statistics in the manuscript, including the appropriateness of statistical tests used?** Yes: The statistical analysis for Fig.3d needs to be checked.

**Were you able to directly test the methods?** No.

**Comments to author:**

Summary

This manuscript delivers the utility of Extru-seq for off-target analysis, which shows advantageous aspects for both in vitro and cell-based off-target analysis methods. The authors developed an extruder-assisted off-target identification method, named Extru-seq, for which RNP comprising Cas9 and gRNA is pre-incubated with cells and the pre-treated cells are lysed through the pores of an extruder. This strategy is suggested to identify off-targets under cellular conditions with lower false-positive rates and higher sensitivity, compared to cell-based assays. The overall strategy is attractive and thus is believed to provide a complement assay to the previous methods. Nonetheless, several issues need to be addressed before Extru-seq is introduced to many researchers, which could help scientific community fully adopt this technology.

Major points:
1.This manuscript relies on a very weighty hypothesis that the NHEJ process would die in the filtered vesicles. However, the degree of cell death are dependent on the duration after cell rupture and it is highly likely that the NHEJ machinery is alive for some time following rupture. Could you consider this time-dependency on the manuscript? For the time being, it is likely that a fraction of sites are repaired by NHEJ process.
2.In relation to the comment above, the cut rate for Extru-seq is lower than that for Digenome-seq. This discrepancy may imply that some portion of cutting sites are repaired by NHEJ process. The relevant data needs to be provided through deep-sequencing analysis, which should include a time (duration after rupture)-dependent indel frequency.
3.Figure 3e indicates that GUIDE-seq and Extru-seq is likely to produce similar off-target prediction results, and also that DIG and Extru-seq show somehow similar results. But, how about DIG and GUIDE-seq? I can't find the relevant data in Figure 3e.
4.The reliability and utility of an analytical method can be evaluated in terms of sensitivity and specificity. Sensitivity refers to the predictability of true positive off-targets and specificity is related to the correct prediction of true negative off-targets. In this study, the authors used filtering options that have been used for Digenome-seq as a default value without varying the options and monitoring the results. That is, the alteration of the filtering options may affect the overall sensitivity and specificity. Thus, the authors may need to monitor the changes in specificity and sensitivity by altering the filtering options and find the optimized filtering options specific to Extru-seq. This attempt may improve the utility of Extru-seq in a variety of applications.
5. In spite of the technical advantage of Extru-seq over other off-target methods, the chromatin and epigenetic status are highly likely to differ between dividing cell lines in vitro and most non-dividing cells in vivo. This limitation needs to be described in discussion section, particularly by mentioning the primary cell data.
6. Line 34: "It is also somewhat redundant, unless these methods are proven to generate mutually exclusive results". This sentence is not persuasive because the aim for adopting multiple methods is to ensure that the multiple methods show similar results and thus the derived results are convincing. Data used for clinical trials requires to submit convincing, not non-redundant data. Plus, the FDA guideline also requires to use multiple off-target assay methods, rather than to rely on a single method. By keeping this in mind, I cautiously ask that the overall tone of argument throughout the manuscript needs to be revised.


Minor points:

1. The terminology used for GUIDE-seq, "copy number" may be misleading readers. I suggest to replace with a more adequate terminology like "sequence read counts".

2. In Figure 3d, all the comparisons were not significant irrespective of variables used. Is it correct? I don't understand why the GUIDE-seq shows non-significant results with that of Dig-seq.

3. The deep sequencing analysis data need to be provided to evaluate how the off-target analysis was conducted. How could you interpret if a certain loci has an indel mutation frequency less than 1.0%? Is it an off-target site or not? And how about less than 0.1% or 0.05%. This issue need to be dealt with in a more quantitative manner.

4. The off-target activity should be evaluated in terms of the on-target level vs off-target level in certain cellular conditions. Merely the number of off-target sites does not provide sufficient information on off-target analysis. The authors need to consider this fact in the development of Extru-seq.

**Reviewer 2**

**Were you able to assess all statistics in the manuscript, including the appropriateness of statistical tests used?** No.

**Were you able to directly test the methods?** No.

**Comments to author:**

Kwon et al present a new assay, Extruseq, to find off-target sites of CRISPR guides. They write "predict", which I like, unlike other paper, they do not seem to overly hype the method. I'm a computational biologist, so can say less about the wet-lab aspects, but if the method even vaguely works as advertised, it will be a useful method, as Guide-seq doesn't work well in many cell types und Digenome seq is known to miss a lot of off-targets. The paper shows that Extruseq, like similar assays, misses some off-targets and finds too many, but that is not different from the other assays. The method has the pontential to be of broad utility, off-targets are a big issue with CRISPR, especially for therapeutic applications. I'll mostly comment on the data-analysis aspects of the paper. The authors have compared their methods against three other methods, which earns my respect. I think it's the first cross-assay off-target comparison that I've seen in the literature, apart from Anderson et al by Genentech, but with more and different assays.

Major issues:

- On page 12 you describe how you determined Extru-seq off-target sites. This seems crucial for the paper and to reproduce this assay by other groups. But I don't understand this section at all, even though I should be an expert on this. You write that "due to unknown technical reasons, false positive candidates (non-cleavage sites according to IGV) were not effectively filtered by Digenome-seq standalone program". Well, that would not be surprising, I imagine that what works for Digenome-seq doesn't necessarily work for Extru-Seq? There may be special filtering cutoffs in the software that are causing trouble with your sequencing files? "non-cleavage sites according to IGV", this is not a description of the problem. IGV is just a viewer for genome reads. What was the problem? At the least you could attach a screenshot from IGV that shows what you think the problem is. For something that could possibly become be a crucial problem of Extruseq, it would be nice to know more about how you determined its final set of off-targets. If you were able to do something manually, I'm sure that you can write software to automate it or at least describe what you did.

You go on to write that used the "GUIDE-seq program" (which one? Which version?). "Off-target information from Cas-OFFinder was used for filtering" = So now you're adding an in-silico filtering step, after ExtruSeq? "Final off-target candidates were determined by IGV analysis" = IGV is a genome browser, not an analysis. This sentence is like writing "we looked at the data and manually determined for every data point, out of possibly thousands of data points, what looked good and what didn't look good". How did you do this filtering? Do you have a table of all the off-targets and how you classified them manually? How many were there initially and how many did you remove? Can you attach this table as a supplement? I don't want to annoy you, but if you attach this table to the paper, then later some computer science people, or AI fanatics, can use your table to automate this method and no more manual filtering would be necessary anymore.


- Fig 4a: any hypothesis what went wrong with ExtruSeq on PCSK9 in human? Why does it overpredict so much here? In the intro, you write that Site-seq overpredicts a lot, but here, Extruseq doesn't look much better.

- "Results from Extru-seq showed high p-Values only when compared with results from cell-based GUIDE-seq" - did you mean to write "low p-Values" here? "High P-Values" means "little similarity". But Guide-Seq is quite similar to Extru-Seq (as you write yourself and show in the figures)


- Can you say why you used the CROP scores to rank in-silico predicted off-targets? I've never heard of them before. Off-targets are usually ranked by other scores, e.g. the CFD scores of Doench et al. Do you think the CROP scores are better? If so, that would be very good to know (and Doench et al I bet would be interested). From my reading of the CROP software, it doesn't actually output its own scores, but both Hsu and CFD scores. If so, which ones did you use? Also, as far as I know what the software outputs are not "probabilities" (the word in your text), but just heuristic scores, they are good for ranking (so what you need), but they don't sum up to 1 and are not probabilities.


Minor issues:

- I find the Schroedinger's cat analogy strange in a Genome Biology paper. It has a taste of a math/physics paper or a computer science paper where analogies and references/jokes are more common than in life sciences. OK, Extruseq has properties of both cell based and in-vitro methods, I agree, but to me that is very different from Schroedinger's cat, because that cat has the special property that it gets killed, whereas Extraseq is not in a state and certainly cannot get killed. The analogy seems rather stretched.

- typos and weird English in some places of the manuscript, e.g. page 7, second paragraph: "GUIDE-seq missed out valid off-target", "IND study that (...) would risk missing out valid off-target"

- "However only the genomic sites with <= 3 mismatches or <= 2 mismatches and 1 DNA/RNA bulge were identified computationally that valid off-targets with >3 mismatches would be missed out" - I cannot parse this English sentence, sorry. What does it mean?

**Authors Response**

**Point-by-point responses to the reviewers’ comments:**

Reviewer #1

*This manuscript delivers the utility of Extru-seq for off-target analysis, which shows advantageous aspects for both in vitro and cell-based off-target analysis methods. The authors developed an extruder-assisted off-target identification method, named Extru-seq, for which RNP comprising Cas9 and gRNA is pre-incubated with cells and the pre-treated cells are lysed through the pores of an extruder. This strategy is suggested to identify off-targets under cellular conditions with lower false-positive rates and higher sensitivity, compared to cell-based assays. The overall strategy is attractive and thus is believed to provide a complement assay to the previous methods. Nonetheless, several issues need to be addressed before Extru-seq is introduced to many researchers, which could help scientific community fully adopt this technology.*

**Response**: We greatly appreciate the reviewer’s positive general statements (“identify off-targets under cellular conditions with lower false-positive rates and higher sensitivity, compared to cell-based assays.”, and “attractive and thus is believed to provide a complement assay to the previous methods.”).

Major points:

*1.This manuscript relies on a very weighty hypothesis that the NHEJ process would die in the filtered vesicles. However, the degree of cell death are dependent on the duration after cell rupture and it is highly likely that the NHEJ machinery is alive for some time following rupture. Could you consider this time-dependency on the manuscript? For the time being, it is likely that a fraction of sites are repaired by NHEJ process.*

**Response**: We thank the reviewer for bringing this important issue to our attention. As suggested, we have performed additional experiments to measure the NHEJ mediated repair process. “Second, using the protocol from multiplex Digenome-seq[28], we performed multiplex Extru-seq to measure the change in cleavage rates at five different on-target sites in the presence or absence of 1 μM SCR7, a chemical DNA ligase IV (or NHEJ) inhibitor [29]. If NHEJ were occurring, then the presence of SCR7 should result in an increase in the cleavage rate, an effect that would also accumulate during the incubation step. However, the difference in the average cleavage rates at five on-target sites in the presence or absence of SCR7 was not significant (Supplementary Figure 3j), further indicating that NHEJ does not significantly affect on-target cleavage rates.”

(j)

Supplementary Figure 3 (j) Cleavage rates, % measured using qPCR, at five on-target sites. Results were obtained using multiplex Extru-seq in the absence (left) or presence (right) of 1 μM SCR7. The horizontal line represents the average (n=5); unpaired student t-test.

*2.In relation to the comment above, the cut rate for Extru-seq is lower than that for Digenome-seq. This discrepancy may imply that some portion of cutting sites are repaired by NHEJ process. The relevant data needs to be provided through deep-sequencing analysis, which should include a time (duration after rupture)-dependent indel frequency.*

**Response:** Thank you for the additional suggestion. “We further analyzed both cut and un-cut populations of the on-target sites to investigate the degree to which NHEJ occurs after the extrusion step. First, the un-cut population in the Extru-seq sample was analyzed via deep-sequencing, given that indel mutations would accumulate in the un-cut population if the NHEJ process were intact during the incubation period after the extrusion step. However, when the deep sequencing results for the Extru-seq samples treated with Cas9 RNP complexes were compared with those for the untreated control samples, the differences were not significant (Supplementary Figure 3i), suggesting that the level of NHEJ after the extrusion step is not significant.”

(i)

Supplementary Figure 3 (i) Indel frequencies measured using targeted deep sequencing data from the Extru-seq samples shown in (a) through (g), with (orange) and without (blue) Cas9 treatment; unpaired student t-test. Error bars indicate standard deviation. (n = 3).

*3.Figure 3e indicates that GUIDE-seq and Extru-seq is likely to produce similar off-target prediction results, and also that DIG and Extru-seq show somehow similar results. But, how about DIG and GUIDE-seq? I can't find the relevant data in Figure 3e.*

**Response:** A sample number of at least 16 was needed for us to perform Wilcoxon rank tests “Because a sample size of at least 16 is required for using asymptotic nonparametric Wilcoxon rank tests[35, 36], intersections with fewer than 16 samples were not included in this analysis (Supplementary Figure 14).”

The original quote from the publication (Dwivedi AK, Mallawaarachchi I, Alvarado LA: Analysis of small sample size studies using nonparametric bootstrap test with pooled resampling method. Stat Med 2017, 36(14):2187-2205.) is as below: “When using asymptotic nonparametric tests, a sample size of at least 16 is required for using Wilcoxon rank and signed-rank tests”

We tabulated number of samples in the intersections of the Venn-diagram in Supplementary Figure 14. All of the sample numbers in the intersections between GUIDE-seq and DIG-seq were below 16. As a result, we could not perform Wilcoxon rank sum test in this case. (We believe that the use of promiscuous sgRNAs in this study proved its value by providing enough number of off-targets to be analyzed via Wicoxon rank sum test, which is not possible when using targets from other studies.)

(a)

|  | h*PCSK9* | h*Albumin* | m*PCSK9* | m*Albumin* |
| --- | --- | --- | --- | --- |
| GUIDE-seq : Digenome-seq | 137 | 43 | 70 | 12 |
| GUIDE-seq : *in silico* | 119 | 55 | 126 | 25 |
| GUIDE-seq : Extru-seq | 128 | 28 | 93 | 22 |
| Digenome-seq : *in silico* | 3311 | 222 | 128 | 54 |
| Digenome-seq : Extru-seq | 1288 | 30 | 110 | 17 |
| *in silico* : Extru-seq | 1133 | 35 | 189 | 54 |

(b)

|  | h*HBB* | h*VEGFA* | h*FANCF* |
| --- | --- | --- | --- |
| DIG-seq : Digenome-seq | 17 | 29 | 24 |
| DIG-seq : Extru-seq | 12 | 28 | 11 |
| DIG-seq : GUIDE-seq | 4 | 10 | 3 |
| Digenome-seq : Extru-seq | 10 | 38 | 10 |
| Digenome-seq : GUIDE-seq | 4 | 11 | 3 |
| GUIDE-seq : Extru-seq | 4 | 10 | 2 |

Supplementary Figure 14. Number of samples found in the intersections of Venn diagrams showing the overlap between off-target sites predicted by different methods for sgRNAs targeting (a) human *PCSK9*, human *Albuimin*, mouse *PCSK9*, and mouse *Albumin* and (b) human *FANCF*, human *VEGFA*, and human *HBB*. Cases in which n ≥ 16 (the minimum number of samples required for asymptotic nonparametric Wilcoxon rank tests) are colored red.

*4.The reliability and utility of an analytical method can be evaluated in terms of sensitivity and specificity. Sensitivity refers to the predictability of true positive off-targets and specificity is related to the correct prediction of true negative off-targets. In this study, the authors used filtering options that have been used for Digenome-seq as a default value without varying the options and monitoring the results. That is, the alteration of the filtering options may affect the overall sensitivity and specificity. Thus, the authors may need to monitor the changes in specificity and sensitivity by altering the filtering options and find the optimized filtering options specific to Extru-seq. This attempt may improve the utility of Extru-seq in a variety of applications.*

**Response:** Thank you for your suggestion. We dedicated a new section on this issue as below: “**Extru-seq shows the highest area under receiver operating characteristic (ROC) curves than other methods.** One powerful tool for assessing prediction models is the ROC curve, which shows sensitivity and specificity in the y-axis and x-axis, respectively. We constructed ROC curves using sequence read counts (GUIDE-seq), DNA cleavage scores (Digenome-seq, DIG-seq, and Extru-seq), CFD scores (CFD), or CROP scores (CROP) as the metric to predict validation results as a binary classification (Figure 5). When the area under the ROC curves were calculated, Extru-seq showed the highest value (0.83) compared to other methods [GUIDE-seq (0.81), DIG-seq (0.80), Digenome-seq (0.72), CROP (0.69), and CFD (0.68) as shown in Figure 5h]. It is considered that the closer the area under curve is to one, the better the model in predicting validation results. Therefore, the highest value of area under ROC curve of Extru-seq suggests the high performance of the DNA cleavage score of Extru-seq as the binary classifier of the validation results. In addition, the use of different thresholds or cut-off values could affect the number of off-target sites predicted by each method. High area under ROC curve suggest that the chance of finding a meaningful threshold for Extru-seq is higher than that for the other methods. ”

Figure 5. (a-d) ROC curves for GUIDE-seq (purple), Digenome-seq (black), Extru-seq (blue), CROP (green), and CFD (red) for the (a) human *PCSK9*, (b) human *Albumin*, (c) mouse *PCSK9*, and (d) mouse *Albumin* sites. (e-g) ROC curves for GUIDE-seq (purple), Digenome-seq (black), Extru-seq (blue), and DIG-seq (orange) for the (e) human *FANCF*, (f) human *VEGFA*, and (g) human *HBB* sites. (h) Area under ROC curves. The bars represent the mean. Error bars indicate standard deviation.

The meaning of ROC curve was further discussed in the conclusion section “Finally, the strong performance of the DNA cleavage score of Extru-seq as the binary classifier of the validation results was supported by the highest value of area under ROC curves for Extru-seq when compared to the other methods.”

*5. In spite of the technical advantage of Extru-seq over other off-target methods,* *the chromatin and epigenetic status are highly likely to differ between dividing cell lines in vitro and most non-dividing cells in vivo. This limitation needs to be described in discussion section, particularly by mentioning the primary cell data.*

**Response:** Thank you for your input. We now described the limitation in the discussion section as below: “Most of the cell-based methods use ‘surrogate’ cell lines to predict genome-wide off-target sites for human clinical samples. However, a difference might exist in the chromatin and epigenetic status of dividing cell lines *in vitro* and that of most non-dividing cells *in vivo*, as was the case when we compared Extru-seq results from HEK293T cells and MSCs. Therefore, it is suggested that Extru-seq should be performed with clinically more relevant cells instead of surrogate cell lines.”

*6. Line 34: "It is also somewhat redundant, unless these methods are proven to generate mutually exclusive results". This sentence is not persuasive because the aim for adopting multiple methods is to ensure that the multiple methods show similar results and thus the derived results are convincing. Data used for clinical trials requires to submit convincing, not non-redundant data. Plus, the FDA guideline also requires to use multiple off-target assay methods, rather than to rely on a single method. By keeping this in mind, I cautiously ask that the overall tone of argument throughout the manuscript needs to be revised.*

**Response:** Thank you for you suggestions. We now changed the paragraph as below to make the argument sound less aggressive: “Additionally, the use of additional methods may not result in the detection of more off-target candidates. For example, the seven valid off-target sites identified by SITE-seq for NTLA-2001 included all of the valid off-target sites found by GUIDE-seq (which found three valid off-target sites) and Cas-OFFinder (which found three valid off-target sites). In this case, the output from just one *in vitro* method that does not miss any valid off-target sites would be the same as the output from the three methods combined.”

Minor points:

*1.The terminology used for GUIDE-seq, "copy number" may be misleading readers. I suggest to replace with a more adequate terminology like "sequence read counts".*

**Response:** Thank you for your suggestion. We now changed “copy number” to “sequence read counts” throughout the manuscript.

*2. In Figure 3d, all the comparisons were not significant irrespective of variables used. Is it correct? I don't understand why the GUIDE-seq shows non-significant results with that of Dig-seq.*

**Response:** This is the table we used for the analysis.

|  | *FANCF* | *VEGFA* | *HBB* |
| --- | --- | --- | --- |
| DIG-seq | 24.24 | 54.84 | 9.09 |
| Digenome-seq | 32 | 48.94 | 23.53 |
| Extru-seq | 69.23 | 53.49 | 33.33 |
| GUIDE-seq | 75 | 91.67 | 100 |

Here is the result we obtained using Prism 9.1.4.

| Table Analyzed | Data 1 |
| --- | --- |
|  |  |
| Column D | GUIDE-seq |
| vs. | vs. |
| Column A | DIG-seq |
|  |  |
| Mann Whitney test |  |
| P value | 0.1000 |
| Exact or approximate P value? | Exact |
| P value summary | Ns |
| Significantly different (P < 0.05)? | No |
| One- or two-tailed P value? | Two-tailed |
| Sum of ranks in column A,D | 6 , 15 |
| Mann-Whitney U | 0 |
|  |  |
| Difference between medians |  |
| Median of column A | 24.24, n=3 |
| Median of column D | 91.67, n=3 |
| Difference: Actual | 67.43 |
| Difference: Hodges-Lehmann | 65.91 |

P value was 0.1, narrowly missing 0.05. As the biggest difference was observed between DIG-seq and GUIDE-seq, it was no surprise that the rest of the analysis gave ‘ns’ results.

*3. The deep sequencing analysis data need to be provided to evaluate how the off-target analysis was conducted. How could you interpret if a certain loci has an indel mutation frequency less than 1.0%? Is it an off-target site or not? And how about less than 0.1% or 0.05%. This issue need to be dealt with in a more quantitative manner.*

**Response:** Thank you for spotting this. We used the same criteria as the one used by EDITAS Medicine when performing validation experiments for EDIT-101 (Maeder ML, Stefanidakis M, Wilson CJ, Baral R, Barrera LA, Bounoutas GS, Bumcrot D, Chao H, Ciulla DM, DaSilva JA et al: Development of a gene-editing approach to restore vision loss in Leber congenital amaurosis type 10. Nat Med 2019, 25(2):229-233.), which preclinical studies were approved by USFDA. [We also checked similar data from other companies (Intellia and CRISPR TX), but the one by EDITAS Medicine was the most detailed and stringent]. We have mentioned the selection criteria and the table requested (Supplementary Table 4) in the method section below.

“We used the following criteria used by EDITAS Medicine[23] to determine whether the target was validated or false (Supplementary Table 4). First, the indel of the sample must be higher than 0.1% for the sample to be validated. Second, the treated / control ratio must be higher than 2.”.

*4. The off-target activity should be evaluated in terms of the on-target level vs off-target level in certain cellular conditions. Merely the number of off-target sites does not provide sufficient information on off-target analysis. The authors need to consider this fact in the development of Extru-seq.*

**Response:** Thank you for pointing this out. The off-target level would become higher if the on-target activities were higher. One could use specificity ratio, which is the ratio between on-target and off-target activities, instead of off-target indel ratio to compare off-target results from different assays or cell lines. Nevertheless, this issue of standardization is a big problem when assembling a large number of off-target data in the published literature to perform machine learning studies to make *in silico* off-target predictions.

In this study, instead of assembling a large number of off-target studies in the previous literature, we used promiscuous sgRNAs to obtain a large number of off-target loci which were used to compare the performance of different off-target prediction tools. We believe that higher on-target activities of the promiscuous sgRNA would result in higher off-target activities in a large number of off-target loci. The data could be used in a machine learning study to make a better prediction model that accounts for ‘on-target effect’ on off-targets, which the referee has pointed out.

*Reviewer #2: Kwon et al present a new assay, Extruseq, to find off-target sites of CRISPR guides. They write "predict", which I like, unlike other paper, they do not seem to overly hype the method. I'm a computational biologist, so can say less about the wet-lab aspects, but if the method even vaguely works as advertised, it will be a useful method, as Guide-seq doesn't work well in many cell types und Digenome seq is known to miss a lot of off-targets. The paper shows that Extruseq, like similar assays, misses some off-targets and finds too many, but that is not different from the other assays. The method has the pontential to be of broad utility, off-targets are a big issue with CRISPR, especially for therapeutic applications. I'll mostly comment on the data-analysis aspects of the paper. The authors have compared their methods against three other methods, which earns my respect. I think it's the first cross-assay off-target comparison that I've seen in the literature, apart from Anderson et al by Genentech, but with more and different assays.*

**Response**: We thank the reviewer’s positive general statements (“*they do not seem to overly hype the method*”, “*if the method even vaguely works as advertised, it will be a useful method*”, “*The method has the pontential to be of broad utility, off-targets are a big issue with CRISPR”,* and “*The authors have compared their methods against three other methods, which earns my respect. I think it's the first cross-assay off-target comparison that I've seen in the literature”*).

*Major issues:*

*- On page 12 you describe how you determined Extru-seq off-target sites. This seems crucial for the paper and to reproduce this assay by other groups. But I don't understand this section at all, even though I should be an expert on this. You write that "due to unknown technical reasons, false positive candidates (non-cleavage sites according to IGV) were not effectively filtered by Digenome-seq standalone program". Well, that would not be surprising, I imagine that what works for Digenome-seq doesn't necessarily work for Extru-Seq? There may be special filtering cutoffs in the software that are causing trouble with your sequencing files? "non-cleavage sites according to IGV", this is not a description of the problem. IGV is just a viewer for genome reads. What was the problem? At the least you could attach a screenshot from IGV that shows what you think the problem is. For something that could possibly become be a crucial problem of Extruseq, it would be nice to know more about how you determined its final set of off-targets. If you were able to do something manually, I'm sure that you can write software to automate it or at least describe what you did.*

**Response:** Thank you for bringing this important issue to our attention. We attached ‘Supplementary Table 3. Manually excluded false positive off-target sites from Digenome-seq and Extru-seq WGS data visualized using IGV.’ in the revised manuscript as suggested. As the table legend implies, we checked the Digenome-seq site and confirmed that the problem is not specific to Extru-seq and is also found in the Digenome-seq result. Since the topic of this paper is not about Digenome-seq standalone program we just added “[As the developer of a new tool, we checked all the sites identified by Extru-seq with the Integrative Genomics Viewer (IGV). Some of the loci look as if they are false positive candidates (that is, non-cleavage sites according to IGV (Supplementary Table 3)). These false positives were also observed in Digenome-seq. The relevant bam files are available at the NCBI Bioproject (https://www.ncbi.nlm.nih.gov/bioproject/) under accession number PRJNA796642.]” in the method section.

*You go on to write that used the "GUIDE-seq program" (which one? Which version?). "Off-target information from Cas-OFFinder was used for filtering" = So now you're adding an in-silico filtering step, after ExtruSeq? "Final off-target candidates were determined by IGV analysis" = IGV is a genome browser, not an analysis. This sentence is like writing "we looked at the data and manually determined for every data point, out of possibly thousands of data points, what looked good and what didn't look good". How did you do this filtering? Do you have a table of all the off-targets and how you classified them manually? How many were there initially and how many did you remove? Can you attach this table as a supplement? I don't want to annoy you, but if you attach this table to the paper, then later some computer science people, or AI fanatics, can use your table to automate this method and no more manual filtering would be necessary anymore.*

**Response:**

1. We added the version of GUIDE-seq program used in the method section. “For data analysis, GUIDE-seq (1.0.2; https://pypi.org/project/guide-seq/) was used which is compatible with Python 3.”

2. We provided Supplementary Table 3. and relevant files as we answered in the section above. 3. We modified the method section as below “**Assignment of off-target results of Digenome-seq and Extru-seq to CAS-OFFinder results**

Unlike GUIDE-seq and CAS-OFFinder, the standalone Digenome-seq program does not have a sgRNA:off-target alignment function that provides information about the number of mismatches and type of bulge (DNA or RNA) between the guide and off-target site. [The web version of the Digenome-seq analysis tool ([http://www.rgenome.net/digenome-js/#](http://www.rgenome.net/digenome-js/)!) has an optional alignment function with an alignment score that does not provide any information about the number of mismatches or type of bulge.] Instead, we used CAS-OFFinder to identify off-target sites with up to seven mismatches and two bulges relative to the target sequence. The positions of the off-target candidates identified by Digenome-seq and Extru-seq were then compared with those identified by CAS-OFFinder so that the information about mismatches and bulge type from CAS-OFFinder could be assigned to the loci identified by Digenome-seq and Extru-seq.”

4. The mismatch numbers for each off-target locus are provided by the GUIDE-seq analysis program and *in silico* programs like CasOFFinder. For web-version Digenome-seq analysis program, they have an option which be selected to provide alignment information in the format below.

However, it only provides an alignment score and a direction of the target strand, which are not translatable to the number of mismatches. Since we needed mismatch number information labeled to a large number of samples, we could not assign mismatches manually. Instead, we performed Cas-OFFinder to make reference information which we used to label Digenome-seq and Extru-seq results.

*- Fig 4a: any hypothesis what went wrong with ExtruSeq on PCSK9 in human? Why does it overpredict so much here? In the intro, you write that Site-seq overpredicts a lot, but here, Extruseq doesn't look much better.*

**Response:** We have listed the number of candidate off-target sites found in Supplementary Figure 10. The number was 7778 for Digenome-seq and 1383 for Extru-seq that Extru-seq still found at least 5 times lower candidates compared to Digenome-seq.

(a)

Supplementary Figure 10. Validation results from targeted deep sequencing of the top 10 predicted off-target sites for sgRNAs targeting (a) human *PCSK9* For validation, the frequency of indels at the off-target site must be higher than 0.1% and the equation (Indel frequency at the off-target locus)/(Indel frequency in the control with no Cas9 treatment) > 2 should be satisfied^9^. A red * indicates that the target was confirmed manually (Supplementary Table 2).

In addition, we performed ROC curve analysis in response to reviewer 1’s request which were described as below. “**Extru-seq shows the highest area under receiver operating characteristic (ROC) curves than other methods.** One powerful tool for assessing prediction models is the ROC curve, which shows sensitivity and specificity in the y-axis and x-axis, respectively. We constructed ROC curves using sequence read counts (GUIDE-seq), DNA cleavage scores (Digenome-seq, DIG-seq, and Extru-seq), CFD scores (CFD), or CROP scores (CROP) as the metric to predict validation results as a binary classification (Figure 5). When the area under the ROC curves were calculated, Extru-seq showed the highest value (0.83) compared to other methods [GUIDE-seq (0.81), DIG-seq (0.80), Digenome-seq (0.72), CROP (0.69), and CFD (0.68) as shown in Figure 5h]. It is considered that the closer the area under curve is to one, the better the model in predicting validation results. Therefore, the highest value of area under ROC curve of Extru-seq suggests the high performance of the DNA cleavage score of Extru-seq as the binary classifier of the validation results. In addition, the use of different thresholds or cut-off values could affect the number of off-target sites predicted by each method. High area under ROC curve suggest that the chance of finding a meaningful threshold for Extru-seq is higher than that for the other methods.”

For human *PCSK9*, a clear-cut threshold (>1.18) was found at which point high sensitivity (95%) and high specificity (72%) were obtained. If we removed points under the threshold, the sample number decreased from 1384 to 791 for Extru-seq.

Reviewer’s only Figure: Figure 5a with red circle which indicate the clear cut threshold at which point high sensitivity (95%) and high specificity (72%) were obtained.

|  | Sensitivity% | 95% CI | Specificity% | 95% CI | Likelihood ratio |
| --- | --- | --- | --- | --- | --- |
| > 0.04300 | 100 | 91.62% to 100.0% | 66.67 | 43.75% to 83.72% | 3 |
| > 0.3203 | 97.62 | 87.68% to 99.88% | 66.67 | 43.75% to 83.72% | 2.929 |
| > 0.5976 | 95.24 | 84.21% to 99.15% | 66.67 | 43.75% to 83.72% | 2.857 |
| > 1.183 | 95.24 | 84.21% to 99.15% | 72.22 | 49.13% to 87.50% | 3.429 |
| > 2.100 | 92.86 | 80.99% to 97.54% | 72.22 | 49.13% to 87.50% | 3.343 |

Reviewer’s only Table: red circle point from the h*PCSK9* ROC curve corresponds to DNA cleavage score 1.183 at which point high sensitivity (95%) and high specificity (72%) were obtained.

*- "Results from Extru-seq showed high p-Values only when compared with results from cell-based GUIDE-seq" - did you mean to write "low p-Values" here? "High P-Values" means "little similarity". But Guide-Seq is quite similar to Extru-Seq (as you write yourself and show in the figures)*

**Response:** In Wilcoxon rank sum test, null hypothesis is two populations have the same continuous distribution. We tabulated p-values for Wilcoxon rank sum tests in Figure 3 (e). Here high p-values mean high similarity, not little similarity. I understand that usually p values (< 0.05) are more frequently used to test whether the difference is statistically meaningful. In figure 3(e), most of the p values were under 0.05 except for GUIDE-Extru, DIG-Digenome, and DIG-Extru pairs, suggesting that most of the pairs were different to each other except the aforementioned three pairs.

**Figure 3.** Venn diagrams showing the number of predicted off-target sites for sgRNAs targeting (a) human *FANCF*, (b) human *VEGFA*, and (c) human *HBB*, determined by the indicated methods. (d) Validation rates of the off-target sites predicted by DIG-seq, Digenome-seq, Extru-seq, and GUIDE-seq for sgRNAs targeting the *FANCF*, *VEGFA*, and *HBB* genes in HeLa cells. (ns, no significance in two-sided unpaired Mann-Whitney test) (e) p-values obtained by the normalized rank sum test for each pair of off-target prediction methods for sgRNAs targeting *PCSK9* and *Albumin* in human and mouse cells in addition to *FANCF*, *VEGFA*, and *HBB* in HeLa cells. (n ≥ 16 were selected to be analyzed.) Dotted line represents p = 0.05.

*- Can you say why you used the CROP scores to rank in-silico predicted off-targets? I've never heard of them before. Off-targets are usually ranked by other scores, e.g. the CFD scores of Doench et al. Do you think the CROP scores are better? If so, that would be very good to know (and Doench et al I bet would be interested). From my reading of the CROP software, it doesn't actually output its own scores, but both Hsu and CFD scores. If so, which ones did you use? Also, as far as I know what the software outputs are not "probabilities" (the word in your text), but just heuristic scores, they are good for ranking (so what you need), but they don't sum up to 1 and are not probabilities.*

**Response:** Thank you for your suggestion. We used CROP scores since it was from the most recently published paper at the time of the study. According to the paper, CROP scores performed better than CFD scores in predicting off-targets. However, we took the reviewer’s comment seriously, and now we added results for CFD scores as well as CROP scores to compare each other side by side throughout the text.

Minor issues:

*- I find the Schroedinger's cat analogy strange in a Genome Biology paper. It has a taste of a math/physics paper or a computer science paper where analogies and references/jokes are more common than in life sciences. OK, Extruseq has properties of both cell based and in-vitro methods, I agree, but to me that is very different from Schroedinger's cat, because that cat has the special property that it gets killed, whereas Extraseq is not in a state and certainly cannot get killed. The analogy seems rather stretched.*

**Response**: Thank you for your suggestion. Our intention was to use the analogy to express the state (dead-live cat or Extru-seq) that is the superposition of two orthogonal states (states that are clearly distinct to each other; (dead vs live) or (*in vitro* vs cell-based). However, we agree that it could look out of place for some readers. We now changed the cat figure in Figure 1 (c) to cell and DNA in a test tube each representing cell-based and *in vitro.*

**Figure 1.** (a) Genome-wide off-target prediction methods can be classified into three different categories: cell-based, *in vitro*, and *in silico*. (b) Schematic of the Extru-seq method. (c) Hypothesis about the dual properties of Extru-seq, which shares characteristics with cell-based and *in vitro* methods.

*- typos and weird English in some places of the manuscript, e.g. page 7, second paragraph: "GUIDE-seq missed out valid off-target", "IND study that (...) would risk missing out valid off-target"*

*- "However only the genomic sites with <= 3 mismatches or <= 2 mismatches and 1 DNA/RNA bulge were identified computationally that valid off-targets with >3 mismatches would be missed out" - I cannot parse this English sentence, sorry. What does it mean?*

**Response:** Thank you for spotting this. The problematic paragraph has been modified as below. “Close inspection revealed that GUIDE-seq overlooked valid off-target sites that contained one to six mismatches (Figure 4i). As such, an IND study that solely depended on GUIDE-seq[26] would risk overlooking valid off-target candidates. In the case of CTX001*,* an *in silico* method was used to complement GUIDE-seq[35]. However, only genomic sites with either three or fewer mismatches, or two or fewer mismatches and a single DNA or RNA bulge, were identified computationally, such that valid off-target sites with more than three mismatches would be overlooked.”

**Second round of review**

**Reviewer 1**

The authors revised most of the concerns raised in the previous manuscript. In particular, the ROC curve analysis against several methods would be tremendously informative. One of the remaining point is regarding the statistic analysis in Fig. 3d. The reason why DIG-seq and GUIDE-seq appear non-significant would be that the data points are too scattered. In other words, more data points would make this statistical results presented differently. This limitations regarding the wide data range and the low number of data points need to be mentioned in the result section.

Overall, most of the concerns were addressed except one minor point. After the remaining point is cleared, I'd like to recommend that this manuscript be accepted for publication in Genome Biology.

**Reviewer 2**

I thank the authors for their diligent work in addressing all comments. The manuscript looks good to me. Sorry for my confusion the Wilcoxon test, I should have known this.
